# Supplementary material for: Towards a generic prototyping approach for therapeutically-relevant peptides and proteins in a cell-free translation system
Source: Nat Commun. 2022 Jan 11;13:260. doi: 10.1038/s41467-021-27854-9 (PMC8752827; doi:10.1038/s41467-021-27854-9)
Supplement: Supplementary file 1 — Supplementary Information [file 41467_2021_27854_MOESM1_ESM.docx]

Supplementary information

Towards a generic prototyping approach for therapeutically-relevant peptides and proteins in a cell-free translation system

Yue Wu, Zhenling Cui, Yen-Hua Huang, Simon J. de Veer, Andrey V. Aralov, Zhong Guo, Shayli V. Moradi, Alexandra O. Hinton, Jennifer R. Deuis, Shaodong Guo, Kai-En Chen, Brett M. Collins, Irina Vetter, Volker Herzig, Alun Jones, Matthew A. Cooper, Glenn F. King, David J. Craik, Kirill Alexandrov and Sergey Mureev

| 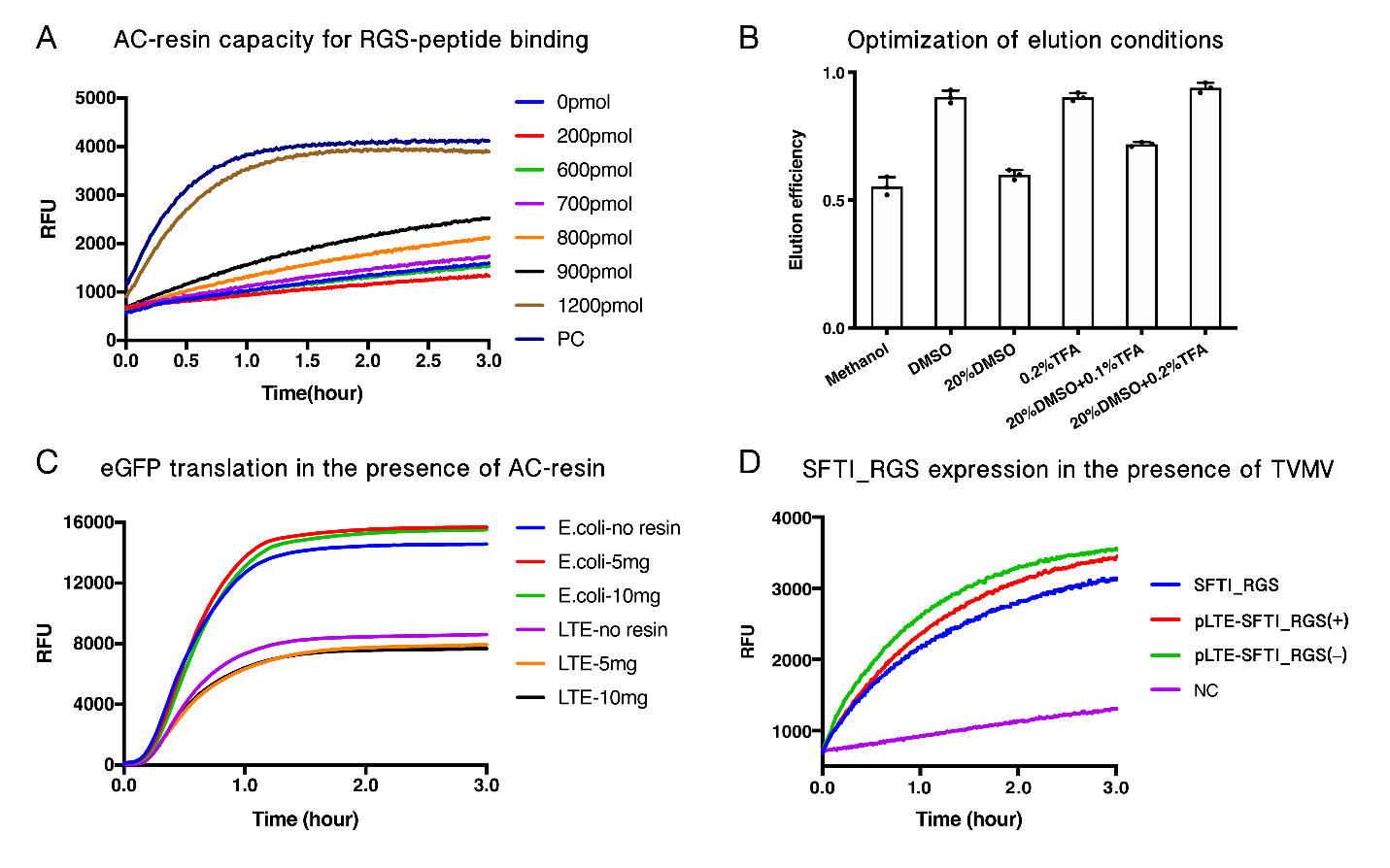 |
| --- |
| **Supplementary Fig. 1** Optimization of various parameters for resin-assisted cell-free peptide production  (A) Estimation of AC-resin binding capacity by monitoring the unbound peptide with AC-assay following incubation of AC-resin amount corresponding to 50 µL of 50% (vl/vl) AC-resin suspension with indicated peptide amounts. Binding capacity was found to be 27 nmol per ml of 50% AC-resin suspension.  (B) Normalized elution efficiencies of different organic solvents. Elution efficiencies are plotted as initial rates of AC-assays performed with RGS peptides following their elution from 10 mg of AC-resin. The graph represents the results as means ± s.d. of n=3 independent AC-assays.  (C) Effect of co-translational addition of indicated resin amounts into 20 μl of reaction mixture on GFP expression efficiency from pLTE-GFP in *E. coli* S30 (Ec CFS) and *Leishmania* (LTE) cell-free translation systems.  (D) Quantification of SFTI-RGS peptide fusion expressed in Ec CFS from pLTE-SFTI-RGS harboring (TVMV-SFTI-RGS) or not (SFTI-RGS) the tvmv-cleavage site in the absence (-) or presence (+) of 50µg/mL tobacco vein mottling virus (TVMV) protease. The mass corresponding to oxidized SFTI-RGS product eluted with DMSO and lacking translation leader sequence was confirmed by LC-MS (data not shown). AC-assay reactions primed either with synthetic SFTI-RGS at 1 µM final concentration or with translation reaction lacking template were used as positive (PC) and negative (NC) controls, respectively. |

| 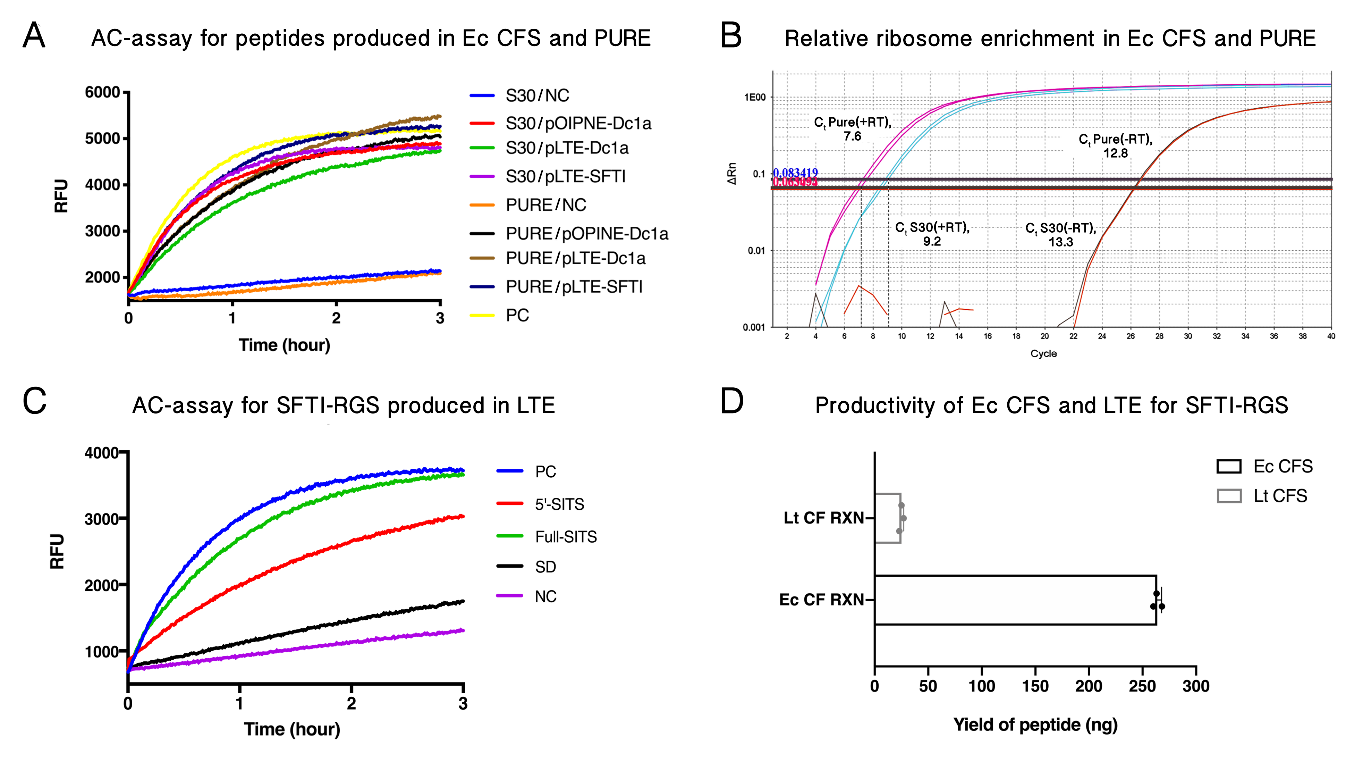 |
| --- |
| **Supplementary Fig. 2** *In vitro* expression of peptide-RGS fusions under the control of various 5’-leader sequences. (A) Quantification by AC-assay of SFTI and Dc1a peptides fused to RGS-tag produced in *E. coli* S30 extract-based (S30) or reconstituted (PURE) translation systems under control of SITS or Shine-Dalgarno (SD) in the context of pLTE or pOPINE vector plasmids, respectively (Table S3). NC and PC denote AC-assay reactions either primed with the reaction lacking template or primed by synthetic MDDRGS-peptide added to 1 μM final concentration, respectively. (B) Comparative reverse-transcription coupled quantitative PCR analysis of 16S rRNA and ribosome particle enrichment between S30 extract-based (Ec CFS) and PURE-based *in vitro* translation reactions. The average threshold cycles (Ct) from the amplification of cDNA following reverse transcription (+RT) of 16S rRNA are indicated. Controls lacking reverse transcriptase (-RT) to account for genomic DNA contamination are shown. (C) Comparative performance of full SITS, 5’-unstructured UTR (5’-SITS) and SD in translation of SFTI-RGS fusion (Table 3) in Leishmania-based CFS (LTE). NC denotes the AC-assay primed with the translation reaction lacking a template. (D) AC-assay quantification of peptide product obtained from 10 µL of AC-resin-assisted Ec CFS and LTE. The graph represents the results as means ± s.d. of n=3 independent AC-assays. |

| 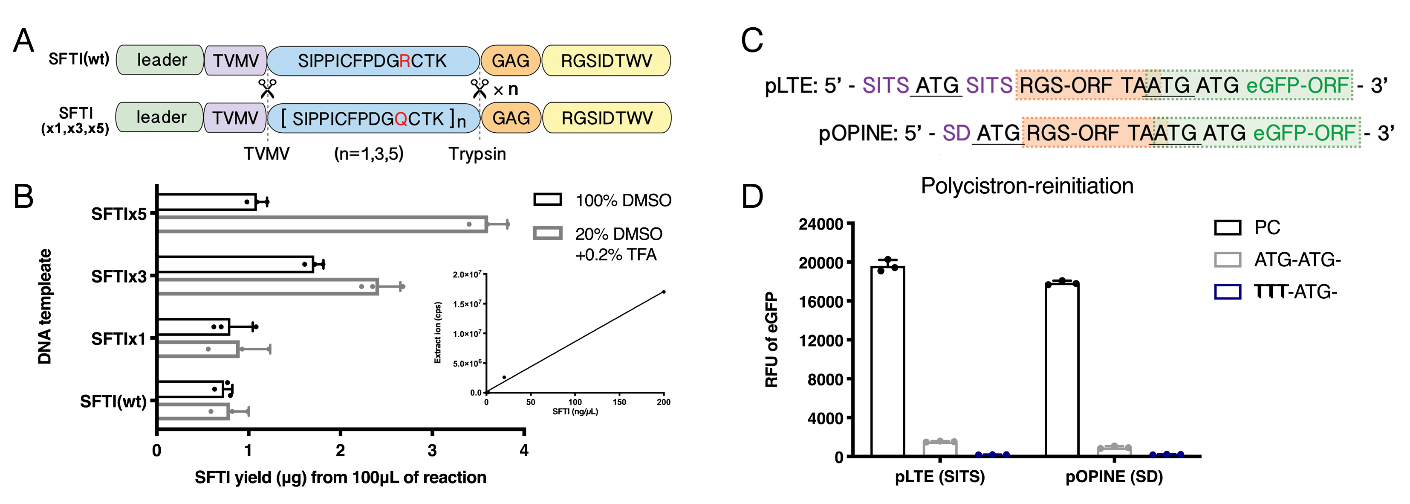 |
| --- |
| **Supplementary Fig. 3** Analysis of peptide yield in resin-assisted Ec CFS as a function of sequential or overlapping ORF arrangement (A) Schematic representation of peptide precursors harboring wt SFTI (top) or SFTI comprising R2Q mutation (in red) arranged as one (x1), three (x3) or five (x5) head-to-tail units (Table 3). Translation leader, TVMV-cleavage site, GAG-spacer and RGS-peptide tag are indicated and color coded. Cleavage positions for TVMV and trypsin are indicated as cutting scissors. (B) Quantitative analysis of monomeric SFTI obtained following purification and tryptic digestion of polymeric peptide-precursors produced in AC-assisted Ec CFS using calibration of LC-MS signal intensity (cps) to the known concentrations of synthetic peptide as shown in the inset. Elution of tri- and pentameric SFTI precursors with absolute DMSO resulted in aggregation while 20% DMSO containing 0.2% TFA supported the efficient elution by decreasing the rate of thiol oxidation. The graph represents the results as means ± s.d. of n=3 independent trypsin treatment reactions. (C) Schematic representation of overlapping ORF arrangements based on pLTE (top) and pOPINE (bottom) constructs (Table 3). Upstream and downstream start-codons are underlined, 5’ and 3’ SITS-parts are shown in purple. SD denotes Shine-Dalgarno motif. RGS-peptide and eGFP-coding sequences are highlighted in orange and green, respectively. (D) Evaluation of reinitiation efficiency at the downstream eGFP-coding ORF. Parental GFP-expressing pLTE and pOPINE constructs (Table 3) were used as positive controls (PC) to indicate the maximal fluorescence yield of eGFP obtainable in the system. ATG-ATG- and TTT-ATG indicate the tested bicistronic construct and the control construct where upstream start-codon is replaced with TTT to account for direct eGFP initiation from the downstream start codon, respectively. The graph represents the results as means ± s.d. of n=3 independent translation experiments. |

| 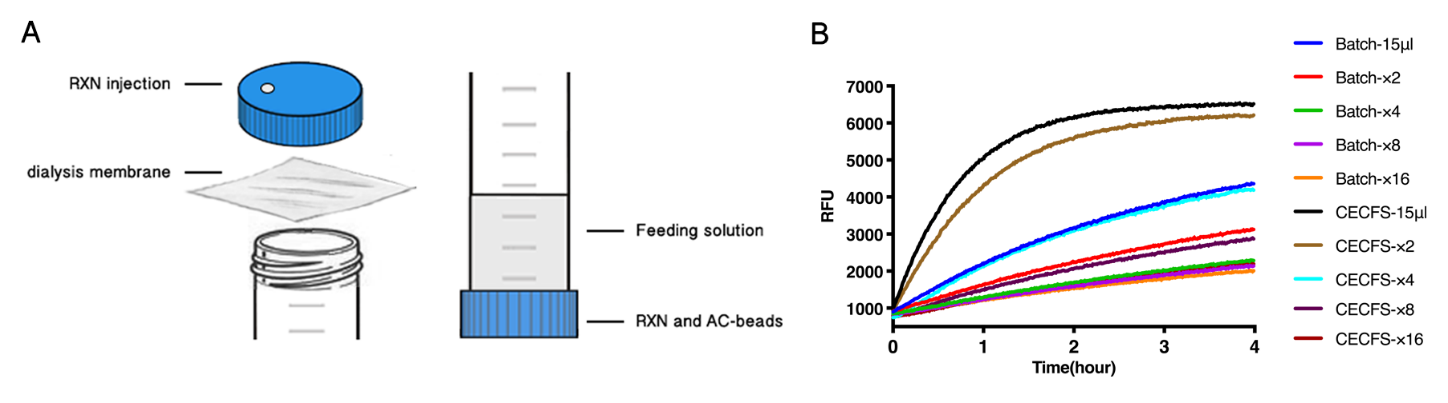 |
| --- |
| **Supplementary Fig. 4** *In vitro* peptide translation in the continuous exchange AC-assisted Ec CFS (A) The dialysis setup is assembled using 50 ml flask harboring the ~1.2 mL reaction compartment bounded by the inner lid rim and dialysis membrane (cutoff 12 kD MW). The sample is injected through the 3 mm opening in the lid which is sealed subsequently with a paper sticker. (B) Comparative analysis of peptide yields by AC-assay from batch and continuous exchange setup using four consecutive two-fold dilutions of original AA139-RGS peptide amount translated from pLTE-AA139-RGS (Table 3) in 15 µL of each reaction. |

| 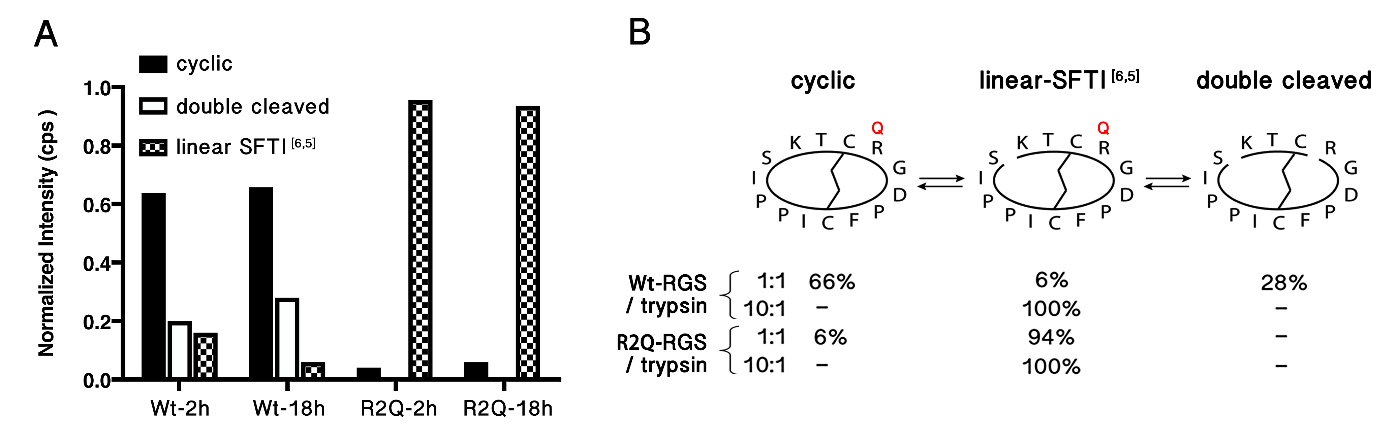 |
| --- |
| **Supplementary Fig. 5** Cleavage and cyclization of SFTI-RGS precursor by trypsin (A) Normalized LC-MS peak intensities of different SFTI forms after extension of trypsin treatment from 2 h to 18 h. (B) Schematic representation of SFTI forms following treatment of SFTI-RGS fusions with different amounts of immobilized trypsin for 18 h. Wt-RGS denotes wild type SFTI-RGS, R2Q denotes SFTI-RGS mutant with Arg-2 replaced by Gln. For details see Supplementary note 4. |

| 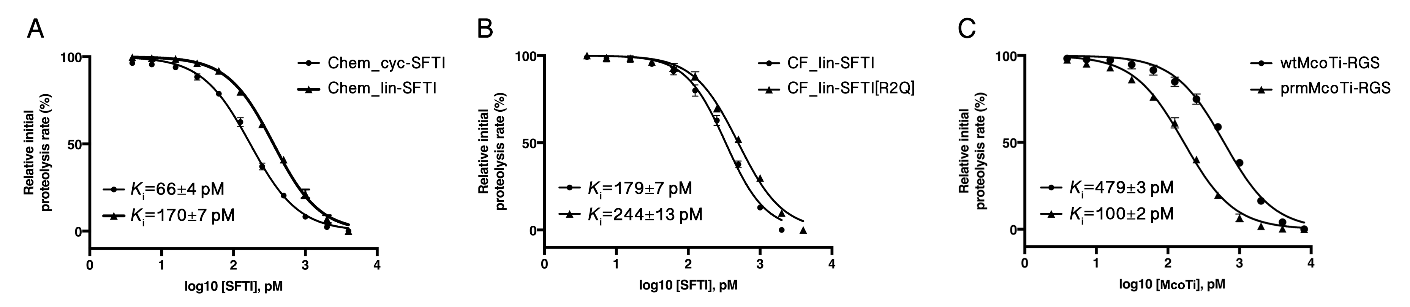 |
| --- |
| **Supplementary Fig. 6** Inhibition of trypsin activity by various forms of SFTI  (A) Inhibition of trypsin activity by chemically synthesized and oxidized linear prmSFTI[6,5] and its cyclic form. The linear prmSFTI form possesses slightly weaker trypsin inhibitory activity than the cyclic form due to its higher susceptibility to secondary cleavage at Arg-2. The graph represents the normalized results as means ± s.d. of n=3 independent assays.  (B) Inhibition of trypsin activity by cell-free (CF) produced linear wt and mutant [R2Q] forms of prmSFTI[6,5]. Cell-free produced wt and mutant forms of prmSFTI-RGS were subjected to trypsin cleavage at 10-fold excess of the peptide over trypsin in order to remove the RGS-tag followed by reaction termination with TFA, quantification of the prmSFTI forms by LC/MS using calibration curve, lyophilization and resuspension in the assay buffer to the appropriate concentration for the trypsin inhibitory assay. The graph represents the normalized results as means ± s.d. of n=3 independent assays. For more details see Supplementary note 4.  (C) Inhibition of trypsin activity by cell-free produced McoTI-II cyclotide. Cell-free produced linear McoTI-II-RGS fusion with wt N/C-termini arrangement (wtMcoTI-RGS) or its circularly permuted linear form (prmMcoTI-RGS) with backbone opened at the scissile bond between Lys-6 and Ile-7 (Fig. 3F) were directly used in the trypsin inhibition assay. Although the apparent K_i_ of prmMcoTI appeared to be slightly higher than the previously reported Ki of 30 pM for cyclic wt McoTI-II^1^, this discrepancy may stem from the incomplete cleavage of the fusion peptide as trypsin is being progressively inhibited with the released prmMcoTI leading to overestimation of inhibitory peptide concentration. Despite the presence of the intact trypsin inhibitory loop in wtMCoTI-RGS, it displayed almost 4-fold higher Ki for trypsin compared to prmMcoTI due to increased entropy of the open backbone as reported previously^2^. The graph represents the normalized results as means ± s.d. of n=3 independent assays. For more details see Supplementary note 5. |

| **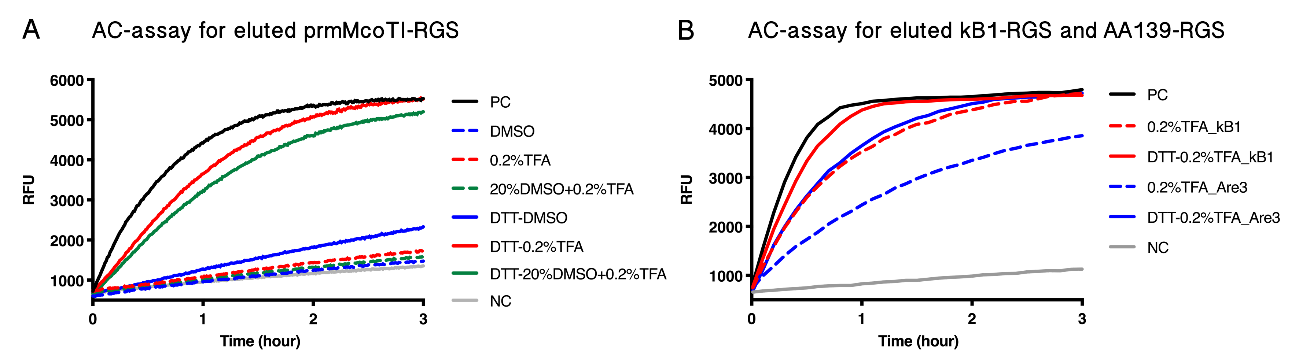** |
| --- |
| **Supplementary Fig. 7** Assessment for cysteine-rich RGS-tagged peptides recovery from AC-resin (A) AC-assay quantification of McoTI-RGS eluted from the resin directly (dashed curves) or following full on-resin reduction with 50 mM DTT (solid curves) by eluents with higher (100% DMSO) or lower (0.2% TFA +/- 20% DMSO) oxidation potential. Synthetic MDDRGS-peptide was used in AC-assay at 1 µM final concentration as positive control (PC). Elution from the resin supplemented into the *in vitro* translation reaction lacking template was used as the negative control for the AC-assay (NC).  (B) AC-assay quantification as in (A) of eluted kalataB1-RGS (kB1) and Arenicin-3 AA139-RGS analogue (Are3) with 0.2% TFA before or after full on-resin reduction. |

| 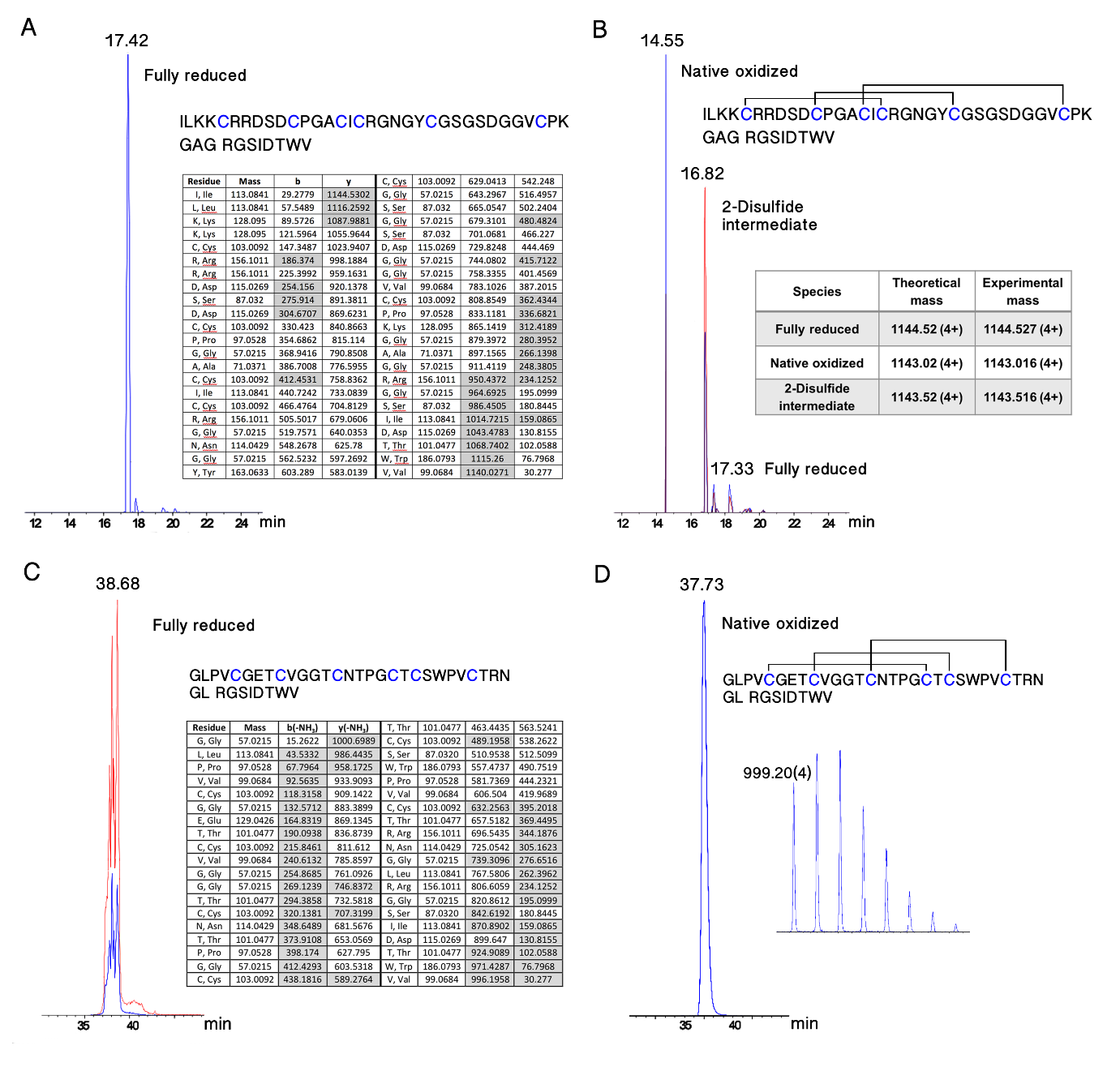 |
| --- |
| **Supplementary Fig. 8** LC-MS/MS characterization of reduced and oxidized forms of McoTI-RGS (A, B) and Kalata B1-RGS (C, D) (A) LC-MS trace and MS/MS spectra of fully reduced prmMcoTI-RGS following 30 min incubation of resin-bound peptide in reducing buffer (50 mM DTT, 50 mM ammonium acetate, pH 8.5) at 25°C followed by elution with 0.2% TFA. (B) Progress of McoTI oxidative folding monitored by LC-MS profiling of intermediates following incubation of resin-bound McoTI in oxidation buffer containing 10 mM glutathione in 0.1 M ammonium acetate, pH 8.5 for 12 h at 25°C. Theoretical and experimentally determined mass (charge=4) of three potential species with different number of disulfide bonds are shown in the inset table. For details see Supplementary note 5. (C) MS/MS-spectra confirming the presence of fully reduced Kalata B1 eluted with 0.2% TFA. (D) Monoisotopic mass peak of oxidized kalata B1 (m/z^4+^ = 999.189) compared to fully reduced theoretical m/z^4+^=1000.69. |

| 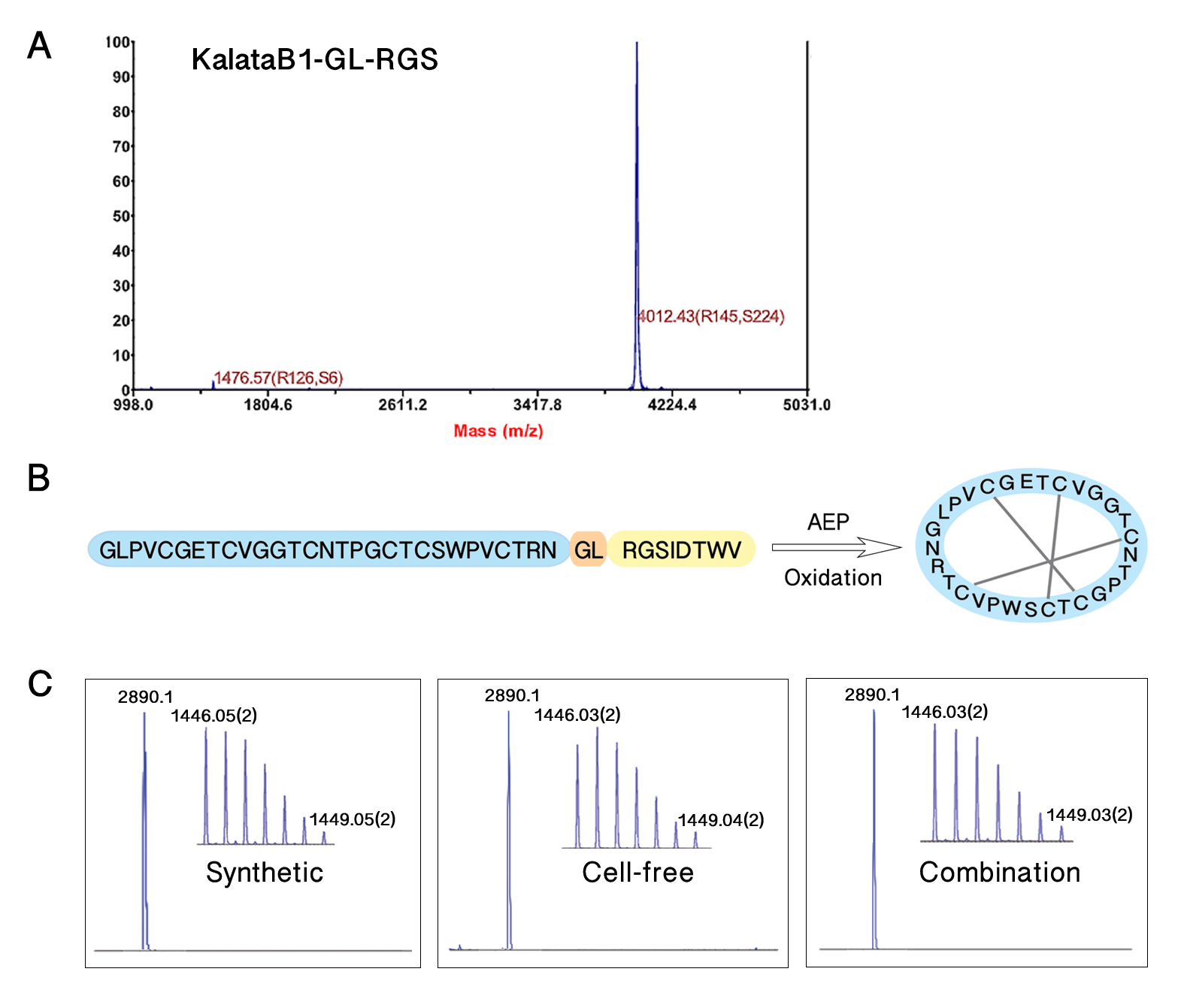 |
| --- |
| **Supplementary Fig. 9** Resin-assisted production and structural characterization of KalataB1 (A) MALDI-MS profile for the eluted KalataB1-GL-RGS peptide precursor; the mass detection range was set from 998 Da to 5031 Da in order to track possible N-terminal truncation products. (B) Schematic for the conversion of reduced KalataB1-GL-RGS precursor to the native KalataB1 following treatment with asparagine endopeptidase (AEP) and subsequent oxidation. (C) HPLC elution traces and mass spectrometry for the cyclic, oxidized synthetic and cell-free produced kalata B1 as well as their overlap combination are shown. The insets with monoisotopic mass peaks at given m/z^2+^correspond to masses of 2890.1 Da for oxidized kalata B1. For details see Supplementary note 6. |

| 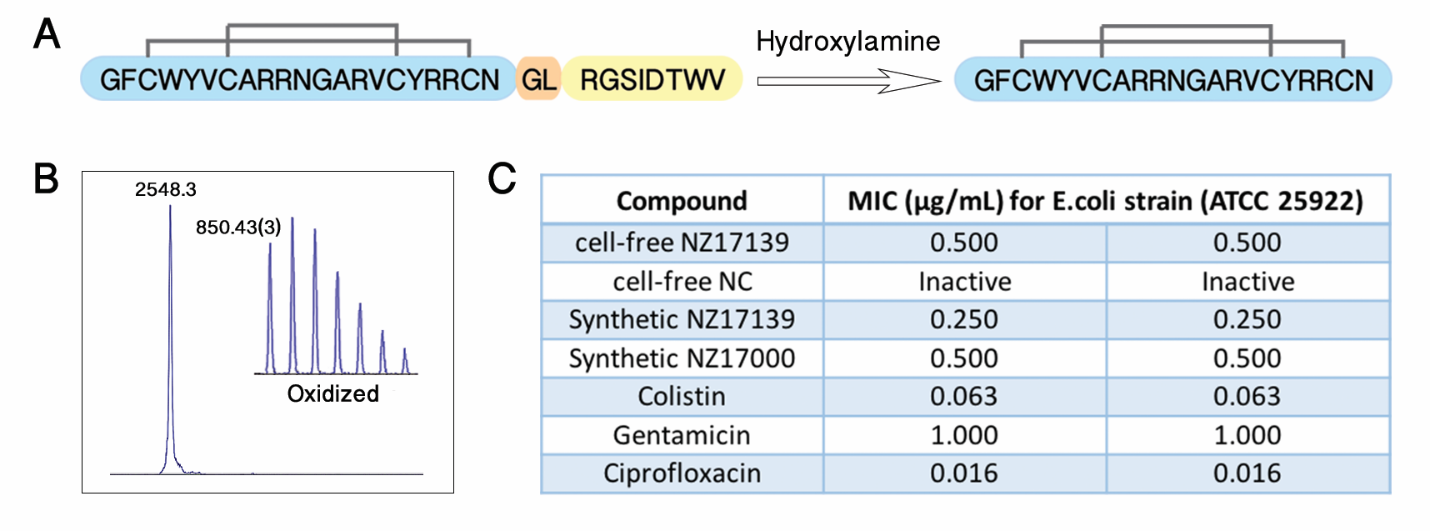 |
| --- |
| **Supplementary Fig. 10** Processing and analysis of Arenicin-3 analogue AA139 (A) Schematic representation of hydroxylamine-mediated RGS-tag removal from AA139-RGS-fusion. (B) HPLC combined with mass spectrometry trace corresponding to eluted peptide containing two disulfide bonds. The inset with monoisotopic mass peaks at given m/z3+ indicates the mass of 2548.3 Da for the oxidized AA139. (C) Table summarizes the Minimal Inhibitory Concentrations (MIC) obtained in antimicrobial activity assay with cell-free produced and synthetic AA139 peptides as well as original Arenicin-3 (NZ17000) precursor and antibiotics used as positive controls. NC corresponds to the eluat of *in vitro* translation reaction lacking the template. The table represents the minimal inhibitory concentrations of n=2 independent biological tests. For details refer to Supplementary note 7 and the Source Data file. |

| 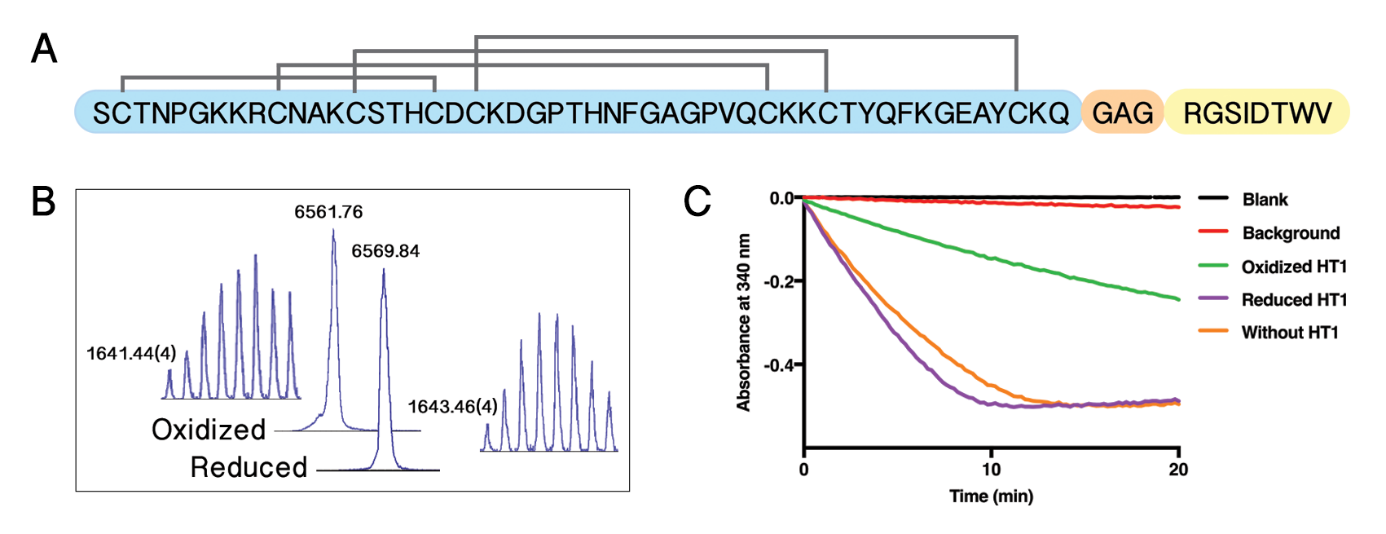 |
| --- |
| **Supplementary Fig. 11** Analysis of oxidized Holocyclotoxin-1 (HT-1) (A) Schematic representation of the oxidized HT-1-RGS peptide. (B) HPLC combined with mass spectrometry traces of both fully oxidized and reduced forms of HT-1. The insets with monoisotopic mass peaks at given m/z4+ correspond to masses of 6561.76 Da and 6569.84 Da for oxidized and reduced HT-1, respectively. (C) Colorimetric assay of dihydrofolate reductase (DHFR) activity of DHFR-Calmodulin chimeric sensor with M13 calmodulin binding peptide. Addition of the oxidized HT-1 peptide to the reaction interferes with the sensor activation. For details see Supplementary note 8. |

| 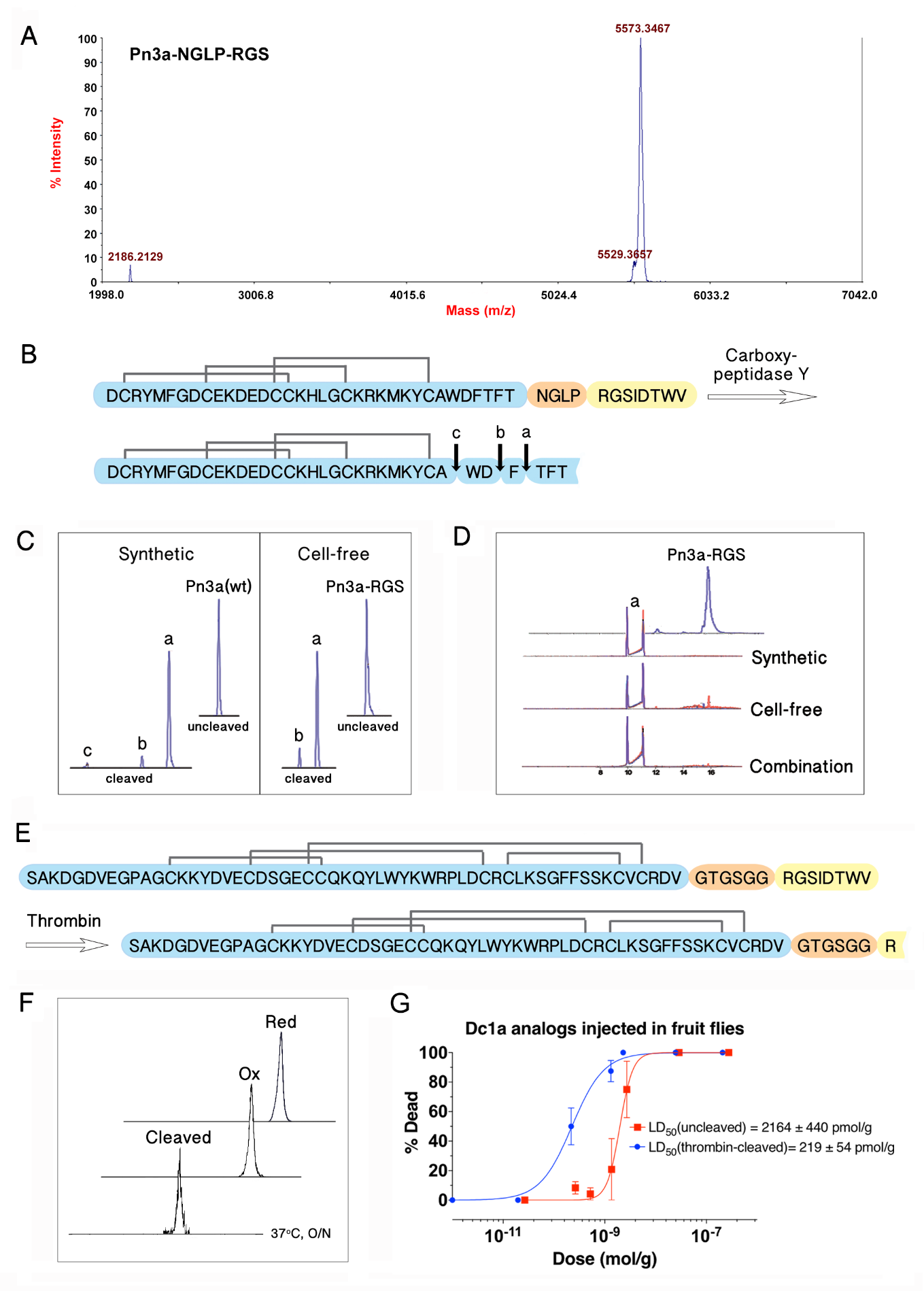 |
| --- |
| **Supplementary Fig. 12** Structural/functional characterization of neurotoxins Pn3a and Dc1a (A) MALDI-MS profile for the eluted Pn3a-NGLP-RGS peptide precursor; the mass detection range was set from 1998 Da to 7042 Da in order to track possible N-terminal truncation products. (B) Pn3A cleavage scheme by Carboxypeptidase Y. Major cleavage site positions are indicated by arrows and the respective truncated peptide fragments are denoted as ‘a’, ‘b’ and ‘c’. (C) MALDI-MS profiles for synthetic and cell-free produced Pn3A before and after cleavage with Carboxypeptidase Y. (D) Co-elution profile analysis of the main cleavage product ‘a’ derived from synthetic wild type Pn3a (wt) and cell-free produced Pn3a-RGS. (E) Schematic representation of RGS tag removal from *in vitro* synthetized Dc1a by thrombin. (F) MALDI-MS profiles of reduced (Red), oxidized (Ox) and thrombin cleaved forms of cell-free produced Dc1a. (G) The median lethal dose (LD_50_) comparison for full-length and C-terminally truncated variants of Dc1a following their injection into female *Drosophila melanogaster* as described previously ^3^. Three repeats of seven doses (each in n=8 fruit flies) were used for each Dc1a analogue to monitor the lethal effects at 24 h after the injection and determine the respective LD_50_ ^4^. The results are plotted as means ± SEM of n=8 independent injection experiments performed in triplicate. For details see Supplementary note 9. |

| 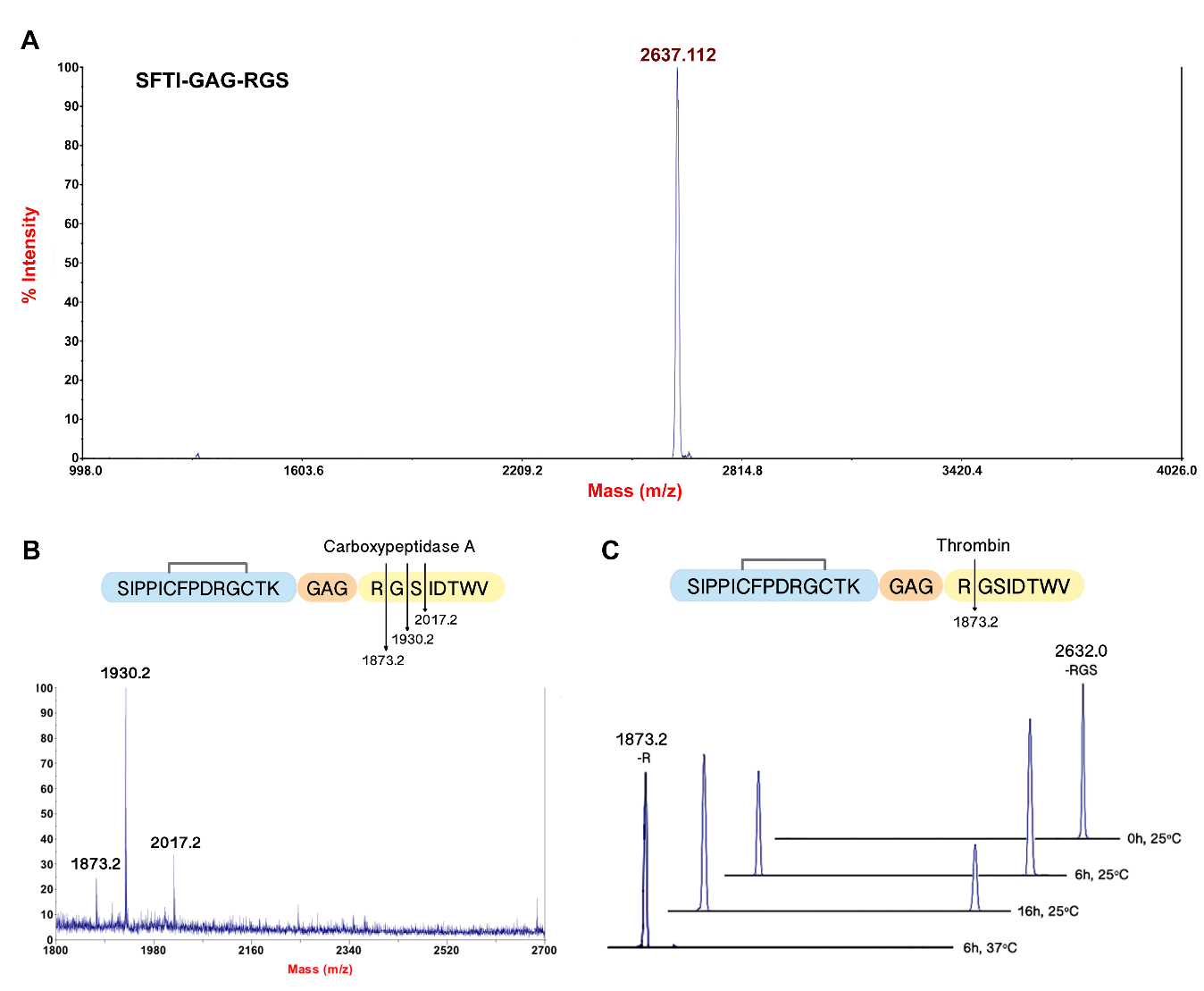 |
| --- |
| **Supplementary Fig. 13** Analysis of SFTI-RGS cleavage with Carboxypeptidase A or Thrombin (A) MALDI-MS profile of SFTI-RGS peptide precursor following its elution from affinity-resin; the mass detection range was set from 998 Da to 4026 Da in order to track possible N-terminal truncation products. (B) MALDI-MS analysis of Carboxypeptidase A cleavage cleavage products. Cleavage reaction was performed with 20 pmol of peptide in a cleavage buffer (25 mM Tris-HCl, 500 mM NaCl, pH 7.5) at 37°C for 16 h with 0.2 U immobilized CPDA-resin (Sigma-Aldrich). (C) MALDI-MS analysis of Thrombin cleavage products. Cleavage reaction was performed with 20 pmol of peptide and 2-unit of thrombin (Sigma-Aldrich) in cleavage buffer (50 mM Tris-HCl, 10 mM CaCl2, pH 8.0) at 37°C for 6-12 h. For details see Supplementary note 10. |

| 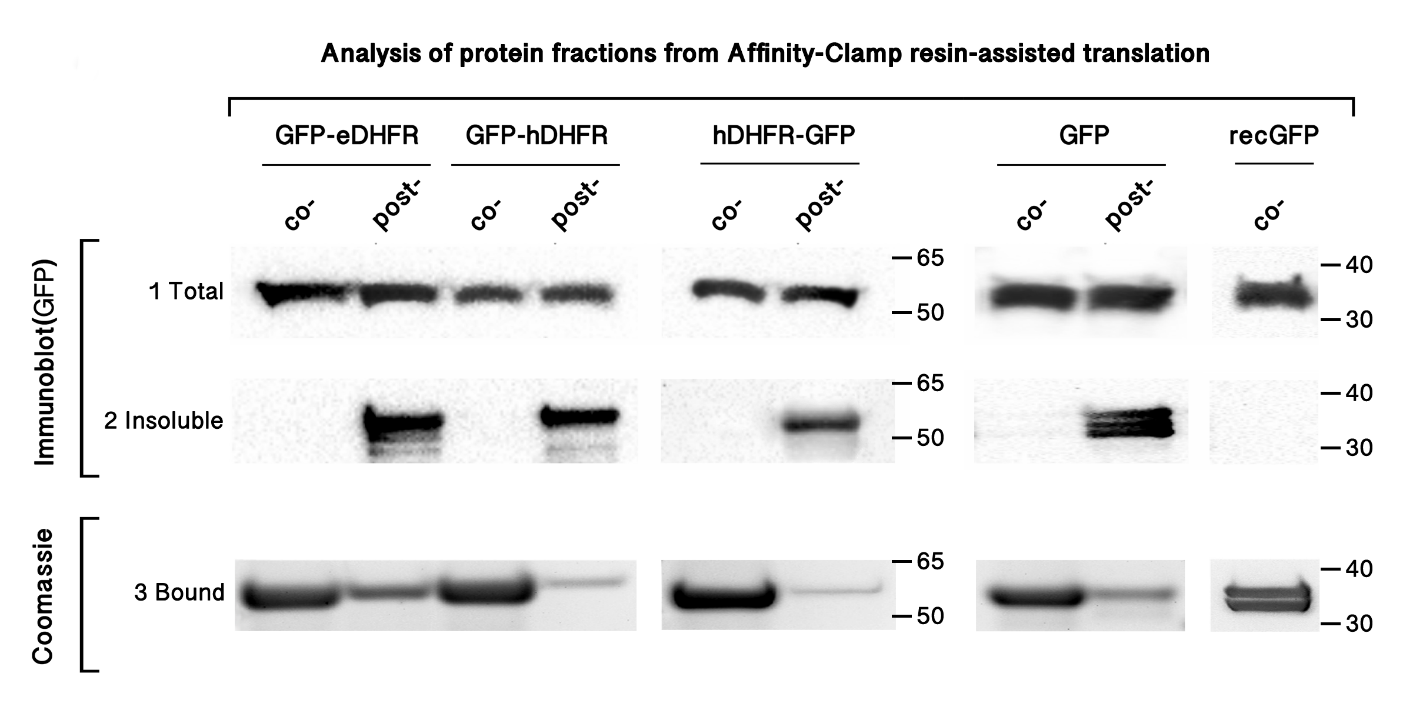 |
| --- |
| **Supplementary Fig. 14** Analysis of protein fractions following co- or post-translational (post-) addition of Affinity-Clamp-coated resin in Ec CFS. Protein fractions corresponding to 5 µL of translation reaction were resolved on SDS PAGE and blotted with anti-GFP antibodies. Panel 1: Unfractionated aliquots of resin-supplemented reactions are shown as total. Panel 2: Respective flow-throughs separated into supernatant and insoluble fractions following centrifugation at 20000 g for 30 min at 4°C. Panel 3: SDS PAGE analysis of resin-bound protein fractions, corresponding to 20 µL reaction eluted by heat-denaturation at 95°C for 5 min with 2xLDS buffer, bands were stained with Coomassie Brilliant Blue. recGFP denotes recombinantly purified GFP carrying both RGS-tag and strep-tag and supplemented into the idle resin-assisted translation reactions at 1/4^th^ of maximal binding capacities, protein fractions corresponding to 0.5 µL reaction were analyzed. |

| 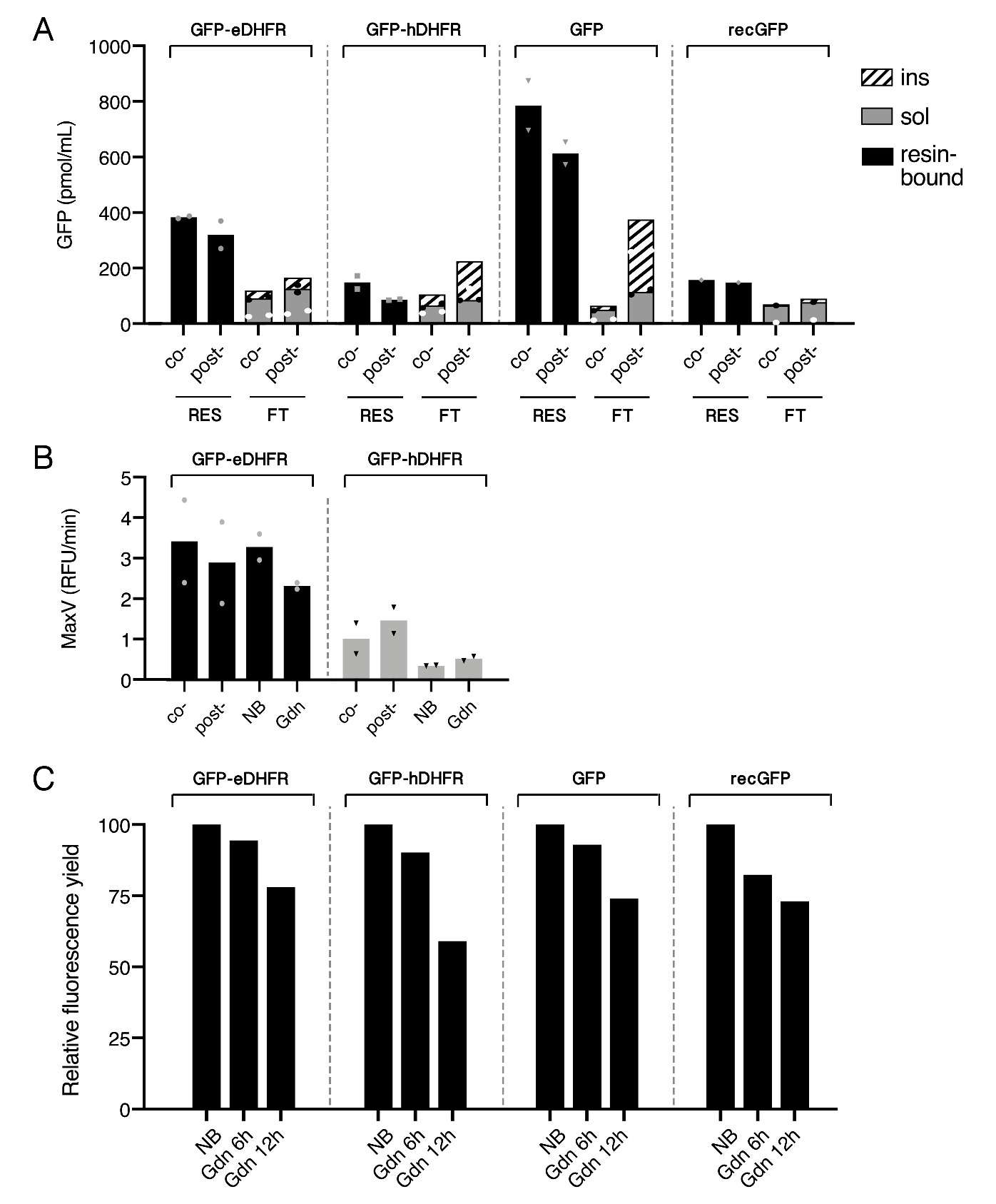 |
| --- |
| **Supplementary Fig. 15** Analysis of folding reporters translated in Affinity-clamp-resin-assisted Ec CFS. (A) Comparison of resin-bound protein fractions (RES) and proportions of soluble (grey) and insoluble (diagonally striped) material in unbound fractions (FT) as a result of co- or post-translational product capture. Fluorescence of insoluble fractions does not account for misfolded (“dark”) protein therefore providing only a relative estimation of protein aggregation degree. Fluorescence units were converted to picomoles of GFP per 1 ml of translation reaction using the respective calibrations of fluorescence against known amounts of free or immobilized GFP (Fig. 16A, D). Folding reporters are indicated above each relevant group of bars. Reactions lacking the template and supplemented with recombinantly purified GFP carrying C-terminal RGS-tag were assembled to control for proteolytic stability of RGS-tag. GFP fluorescence yield corresponds to 50μl reaction. The recGFP remaining in the flow-through (FT) represents the excess of protein over the ligand binding capacity of a given batch of AC-coated resin. The results are plotted as means of n=2 independent translation experiments. (B) Direct analysis of DHFR activity of resin-bound protein fractions following co- or post-translational product capture as described in Methods. Co-translationally captured protein fractions were treated with buffers containing either 2 M guanidine hydrochloride (Gnd) or no denaturant (NB) for 12 h at RT. The differences in activities measured on immobilized protein fractions can be underrepresented. The results are plotted as means of n=2 independent translation experiments. (C) Effect of denaturant treatment on fluorescence activity and retention of AC-resin-bound proteins following incubation of co-translationally immobilized protein samples with 2 M guanidine hydrochloride for 6 h (Gdn 6h) or 12 h (Gdn 12h) compared to protein sample incubated for 12 h in a buffer lacking denaturant (NB). recGFP denotes the purified GFP immobilized onto AC-resin at half maximal ligand binding capacity to indicate the degree of protein dissociation following the incubation with denaturant. |

| 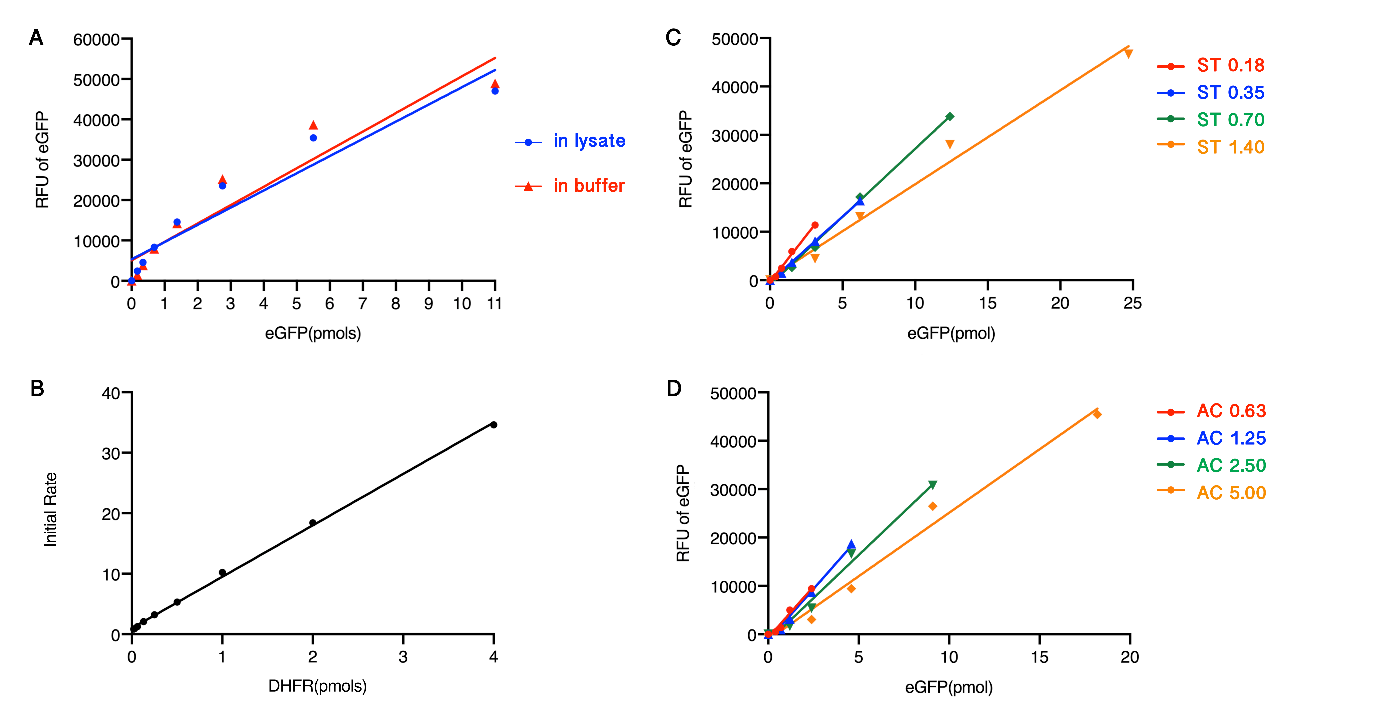 |
| --- |
| **Supplementary Fig. 16** Calibration curves for eGFP and DHFR obtained by assaying activities against known protein concentrations. (A) Calibration curve for free eGFP obtained by supplementing different picomolar amounts of recombinant eGFP into 10 µL of PBS (red) or lysate (blue) and recording fluorescence (Ex 485 nm, Em 525 nm) in white round-bottom 384-well plate using Tecan Spark spectrophotometer with the mirror cut-off set to 50% and the gain set to 52. (B) Calibration curve of DHFR activity obtained by plotting initial rates of NADPH to NADP^+^ conversion against picomoles of bacterial enzyme added into 200 µL of the assay reaction. (C), (D) Calibration curves to establish the relationship between the fluorescence level and pmol amount of resin-immobilized eGFP. Different picomolar amounts of recombinant eGFP carrying both strep-tag and RGS-tag were immobilized to indicated amounts of Strep-Tactin-coated resin (ST) or Affinity-Clamp-coated resin (AC) corresponding to microliters of 50% (vl/vl) suspensions. For all calibrations averaged data of duplicate measurements were fitted using regression coefficients (R^2^). |

| 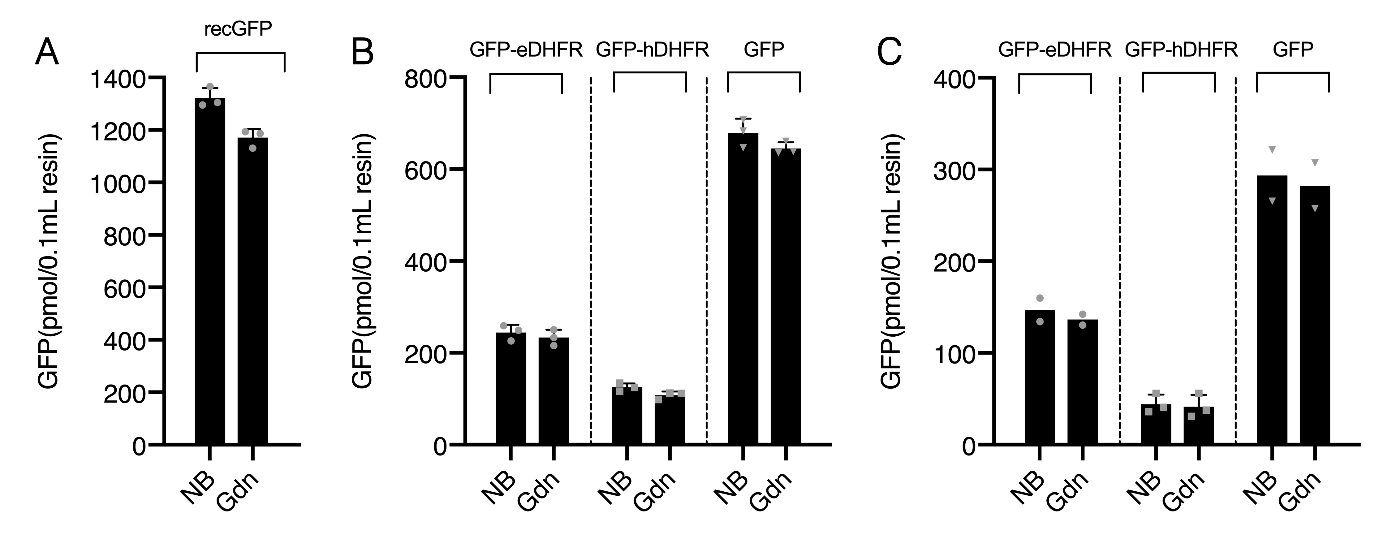 |
| --- |
| **Supplementary Fig. 17** Effect of denaturant treatment on protein retention and fluorescence activity. (A) Immobilization of purified eGFP onto Strep-Tactin-coated resin at half maximal ligand binding capacity (2500 pmol/0.1 ml of 50% (v/v) suspension) and its on-resin retention following treatment with the buffer containing 1.6 M guanidine hydrochloride (GdnHCl) for 2 h at RT with constant agitation at 1400rpm. The graph represents the results as means ± s.d. of n=3 independent translation experiments. (B) Strep-Tactin-immobilized proteins following translation in resin-assisted Ec CFS were incubated with 1.6 M GdnHCl-containing buffer or neutral buffer lacking the denaturant (NB) for 2 h at RT with constant 1400 rpm agitation. The graph represents the results as means ± s.d. of n=3 independent translation experiments. (C) as in (B) but folding reporters were translated in Strep-Tactin-assisted LTE. The results for GFP-hDHFR are represented as means ± s.d. of n=3 independent translation experiments while for the rest of protein samples the results of n=2 independent reactions are displayed. |

| 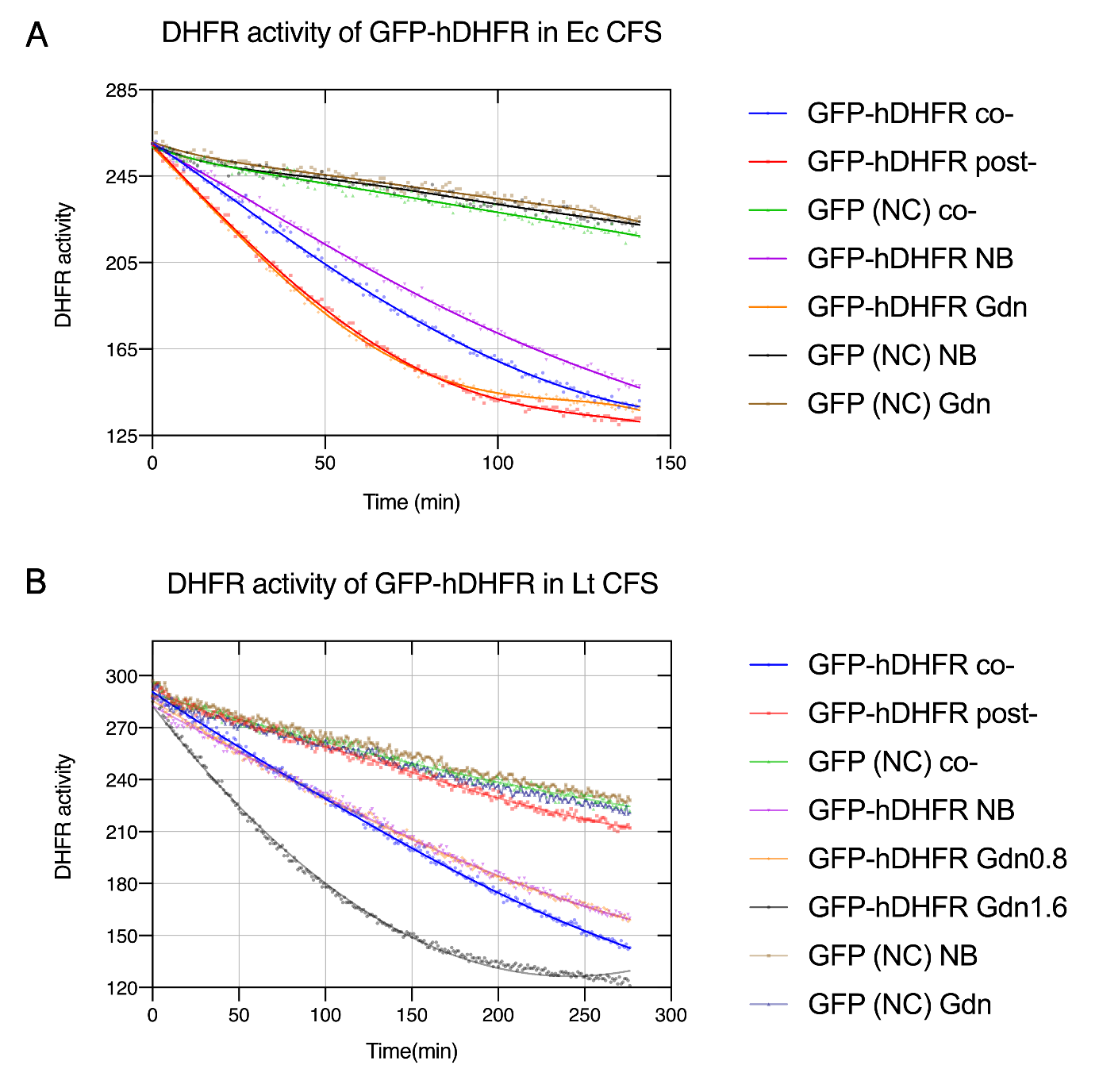 |
| --- |
| **Supplementary Fig. 18** DHFR activity of eluted GFP-hDHFR fractions before or after refolding (A) Fractions of GFP-hDHFR translated in Ec CFS were eluted from Strep-Tactin-resin, amounting to 6 μl of initial 50% (vl/vl) resin suspension, following either co- or post-translational capture (co-/post-) as well as following treatment of co-translationally captured protein either with 1.6 M guanidine hydrochloride (Gdn)-containing buffer or with neutral buffer (NB) free of denaturant. Elutions corresponding to reactions primed with GFP-coding template and processed respectively were used as negative controls: GFP (NC) co-, GFP (NC) Gdn, GFP (NC) NB. (B) as in (A) but GFP-hDHFR was translated in LTE followed by the same downstream treatment. DHFR activity was monitored by a change in extrinsic fluorescence of NADPH upon its conversion to NADP^+^ (Ex 340 nm, Em 375 nm). Curves were fitted with linear or polynomial functions using Excel. |

| 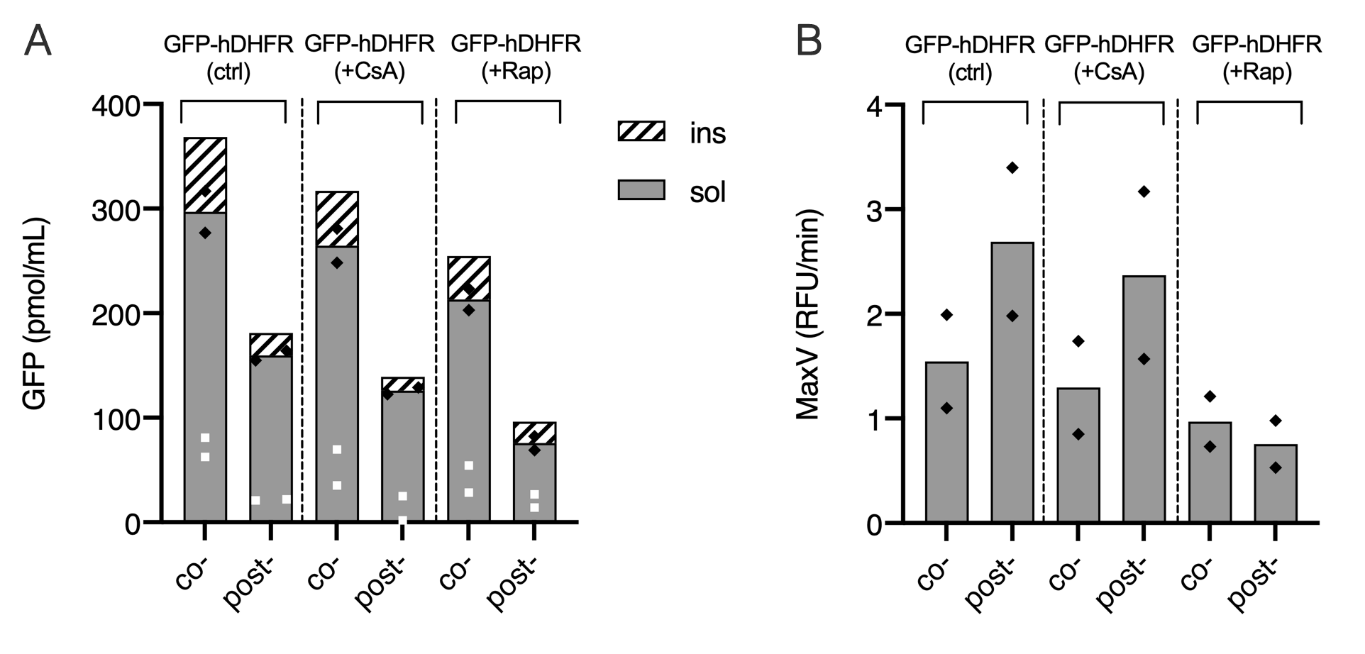 |
| --- |
| **Supplementary Fig. 19** Effect of cyclosporine A and Rapamycin on activity of GFP-hDHFR folding reporter. (A) Fluorescence analysis of resin-bound (RES) fractions following co- and post-translational protein capture. Translation reactions in Ec CFS contained either no added inhibitors of peptidyl-prolyl isomerase (ctrl) or 10 μM of either cyclosporine A (+CsA) or Rapamycin (+Rap). Grey defines soluble fractions either eluted with biotin from the resin or remaining in the flow-through after centrifugation at 20 kg for 30 min at 4°C. Black defines insoluble fractions remaining on resin or removed from flow-through following centrifugation, respectively. Fluorescence units were converted to picomoles of GFP per 1 ml of translation reaction using the respective calibration curves (Supplementary Fig. 16A, C). The results are plotted as means of n=2 independent translation experiments. (B) DHFR activity analysis of eluted GFP-hDHFR fractions following co- or post-translational capture before (co-/post-). The results are plotted as means of n=2 independent translation experiments. |

| 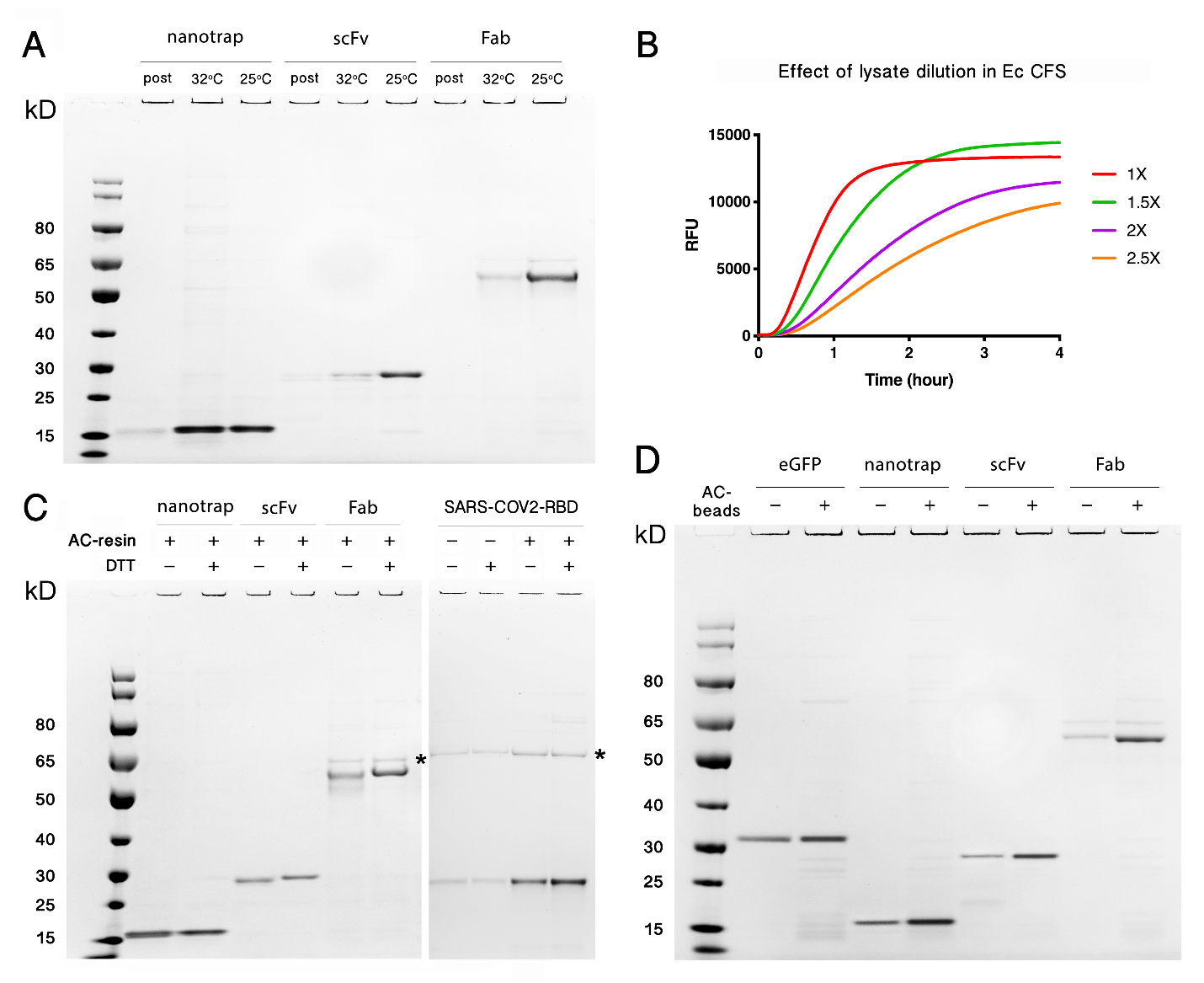 |
| --- |
| **Supplementary Fig. 20** Optimization of translation reaction parameters to improve the yield of soluble protein fraction in AC-assisted Ec CFS. (A) SDS-PAGE analysis of GFP nanobody (nanotrap), anti-HSA scFv and Fab produced at 32°C or 25°C in AC-assisted Ec CFS using pLTE-αGFP-Vhh-RGS, pLTE-αHSA([5fuo](about:blank))-scFv-RGS and pLTE-αHSA([5fuo](about:blank))-Fab-RGS, respectively (Table 3). Control reactions with AC-resin supplemented post-translationally (post) are also shown for each antibody fragment; (B) Effect of S30 extract dilution with the extract buffer on total yield and expression kinetics of eGFP; (C) SDS-PAGE analysis of GFP nanobody (nanotrap), αHSA scFv, αHSA Fab and SARS-COV2-RBD produced at 25°C in AC-assisted Ec CFS with (+) or without (-) 10 mM of extra DTT. Asterisks indicate RBD-binder co-purified from Ec CFS and identified as Hsp60. (D) SDS-PAGE analysis of eGFP (from pLTE-GFP-RGS), GFP nanobody (nanotrap), anti-HSA scFv and Fab produced in AC-assisted Ec CFS at optimal conditions: 25°C, 1.5-fold diluted lysate in the presence of extra 10 mM DTT. Gel images were captured using the ChemiDoc system (BioRad) following band staining with SimplyBlue dye. |

| 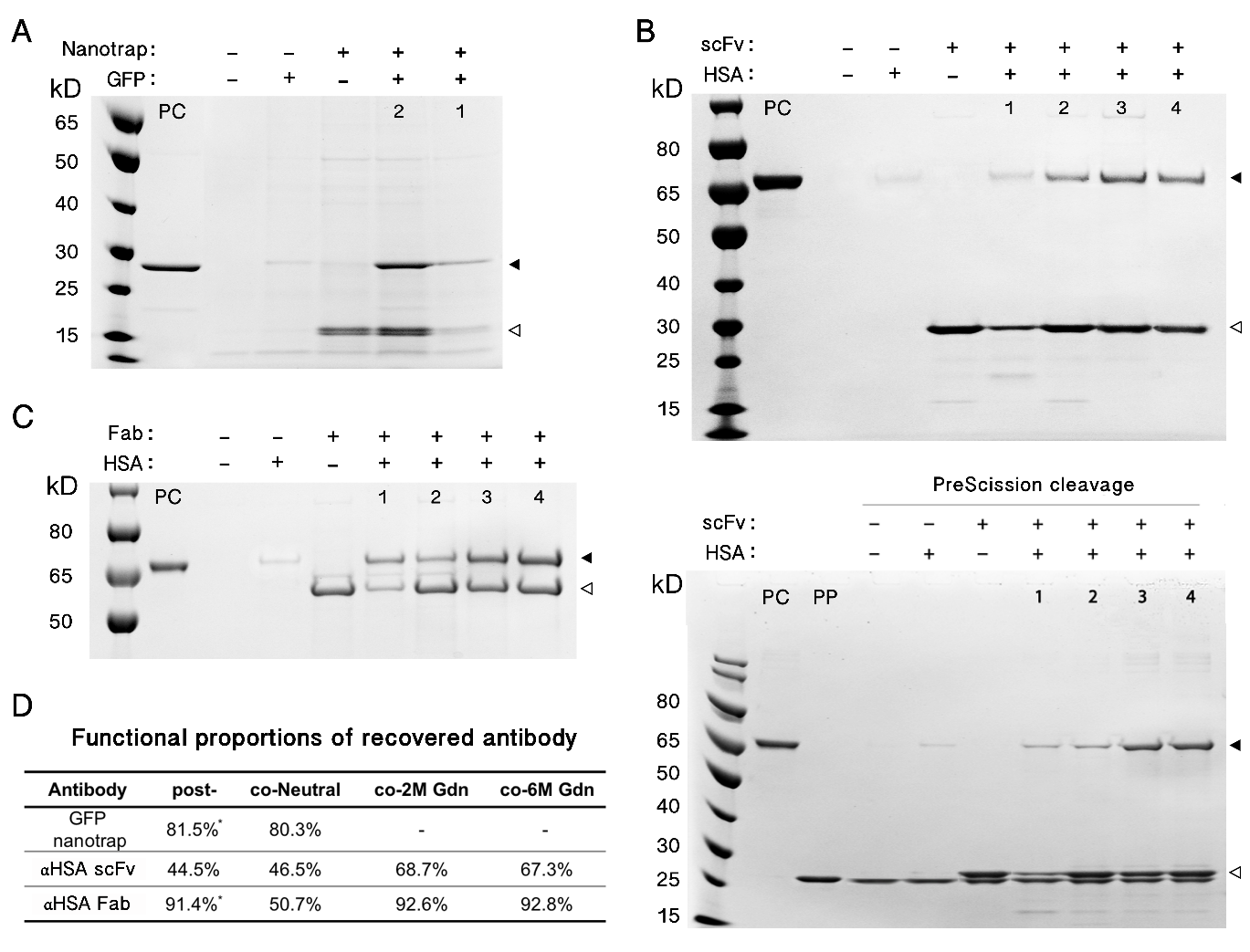 |
| --- |
| **Supplementary Fig. 21** Analysis of folded fractions for antibody fragments expressed in Ec CFS by pulldown assay. 1, post-translational capture; 2, 3, 4 – co-translational capture of translation products onto AC-resin and on-resin treatment with either neutral buffer, 2M GdnHCl or full denaturing/renaturing with 6 M GdnHCl, respectively. Pulldown of respective antigens with αGFP-nanotrap antibody (A), αHSA scFv (pdb:[5fuo](about:blank)) (B) and αHSA Fab (pdb:[5fuo](about:blank)) (C). Resin-bound protein complexes were either eluted via heat-denaturing (A, B top, C) or by Prescission protease (PP) cleavage upstream to RGS-tag (B, bottom); (D) Active protein fractions calculated as the size-adjusted ratios of the respective antigen and antibody intensities derived from non-saturated band integration. Unless specified the resin-bound protein fractions were eluted by heat-denaturation at 95°C for 5 min in 2xLDS buffer. PC stands either for recombinantly purified eGFP (A) or commercial pure HSA (Sigma-Aldrich). Antibody fragments and respective antigens are indicated with open and solid arrowheads, respectively. “PC” denotes the gel slots loaded with antigens only. |

| 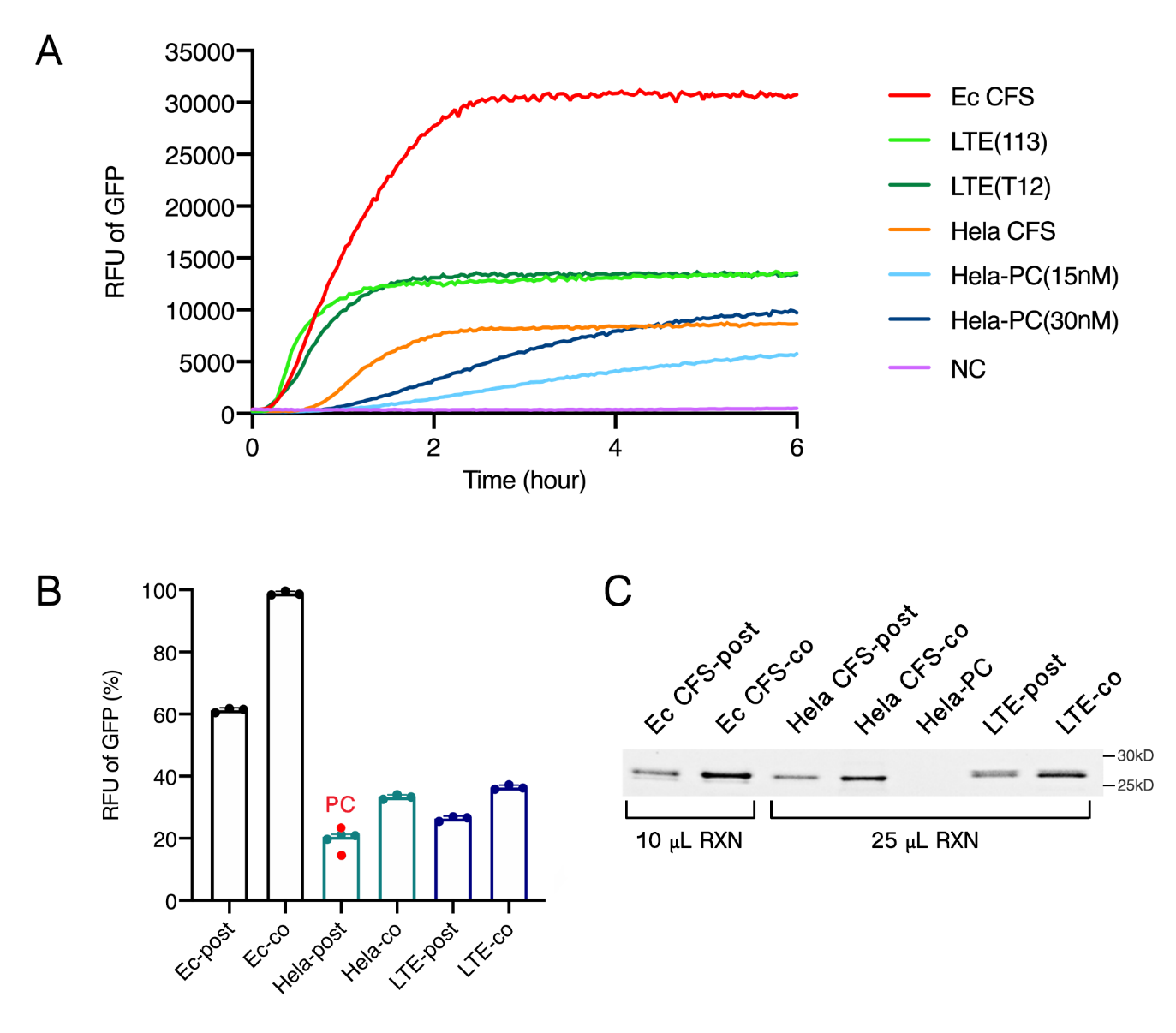 |
| --- |
| **Supplementary Fig. 22** Analysis of GFP expression in Ec, LTE and HeLa-based cell-free translation systems. (A) Fluorescence kinetics of GFP accumulation in 10 μL translation reactions incubated at 30°C for 6 h supplemented with pLTE-GFP-RGS at 30 nM final concentration. Two highly productive batches of LTE - 113 and T12 were selected for comparison. pCFE-GFP template provided by the manufacturer was used as a positive control for HeLa-based translation system performance (HeLa-PC) at indicated concentrations (nM). (B) Relative fluorescence yields for eGFP captured onto AC-resin either co- or post-translationally from 10 μL of the respective translation reactions. The graph represents the normalized fluorescence end-points as means ± s.d. of n=3 independent translation reactions. The end-points obtained from HeLa-PC reactions are overlayed as red dots. (C) Intensities of Coomassie stained eGFP bands from the indicated reaction volumes, eluted and resolved following either co- or post-translational immobilization as described above for Fig. 21. NC corresponds to reaction lacking the template. |

| 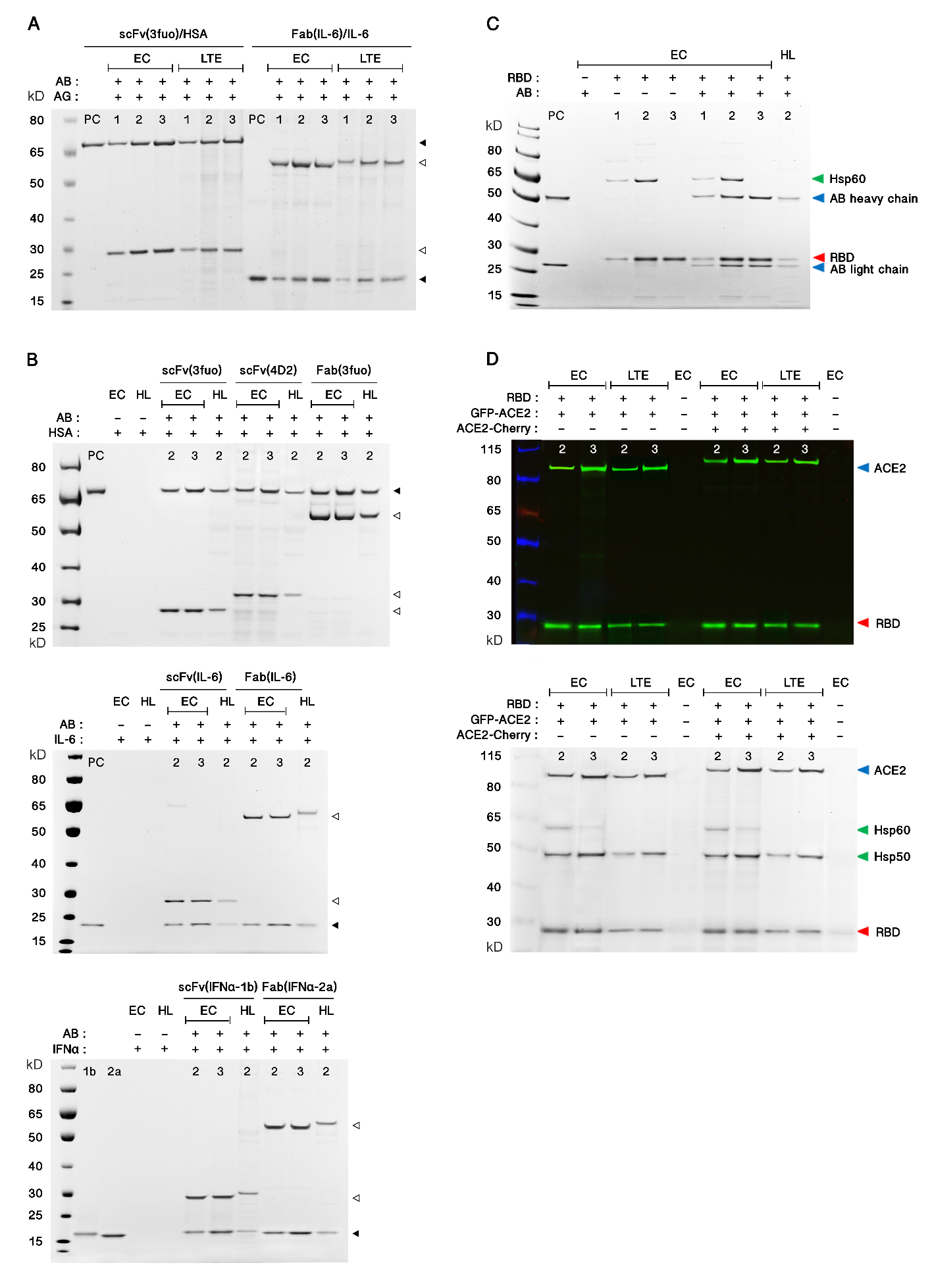 |
| --- |
| **Supplementary Fig. 23** Analysis of folded fractions for disulfide-rich proteins expressed in different cell-free systems. Proteins expressed in Ec CFS (EC), LTE and HeLa cell-free system (HL) were subjected to pulldown with respective antigens (marked by closed arrowheads) following their post- (“1”) or co-translational (“2”, “3”) capture onto AC-resin and either incubation with neutral buffer (“2”) or recycling of misfolding intermediates with 2M GdnHCl (“3”). Pulldown of respective antigens (marked by closed arrowheads) with αHSA scFv (pdb:[5fuo](about:blank)) and αIL-6 Fab (pdb:[4zs7](about:blank)) antibody fragments (AB) produced in EC and LTE (A); αHSA scFv (pdb:[5fuo](about:blank)), scFv(4D2, patent) and Fab(pdb:[5fuo](about:blank)), αIL-6 scFv(pdb:[4cni](about:blank)) and Fab(pdb:[4zs7](about:blank)), αIFNa-1b scFv(pdb:[3ux9](about:blank)) and αIFNa-2a Fab(pdb:[4ypg](about:blank)) produced in EC and HL (B); SARS-COV2 receptor binding domain (RBD) produced in EC and HL (C); pulldown of GFP-ACE2 or ACE2-mCherry produced in LTE with COV2-RBD produced in EC and HL in the presence of BodiPy-FL-conjugated Lysyl-tRNA (refer to Figure 5 legend) visualized either by fluorescent gel scanning (D) or by Coomassie gel staining (E). Green arrowheads indicate Hsp60 and unknown ACE2 binding protein (ACE2BP) pulled by RBD and ACE2 from Ec and LTE lysates, respectively. “PC” denotes the gel slot loaded with antigens only. |

| Fig. 14  Western blots | 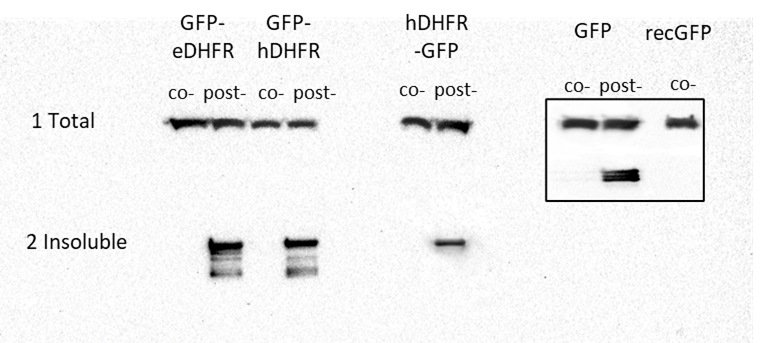 |
| --- | --- |
| Fig. 14  SDS-PAGE, Coomassie stained | 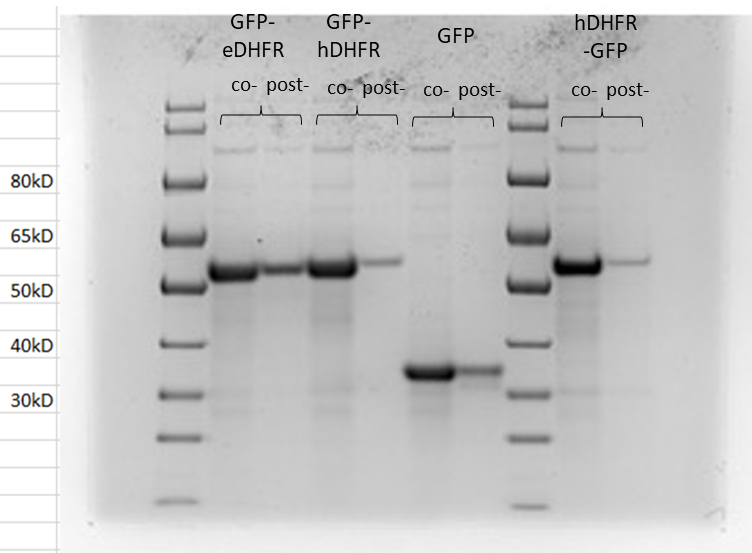 |
| Fig. S20C  SDS-PAGE, Coomassie stained | 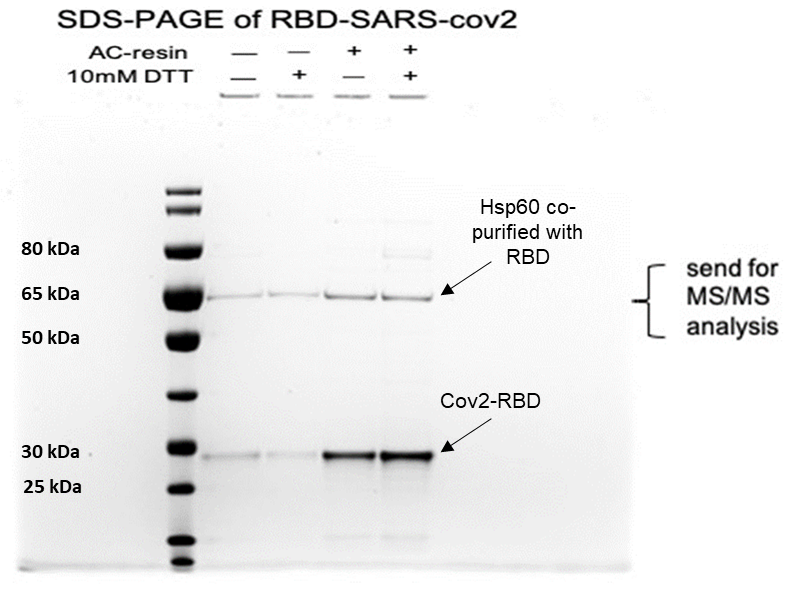 |
| Fig. 21A  SDS-PAGE, Coomassie stained | 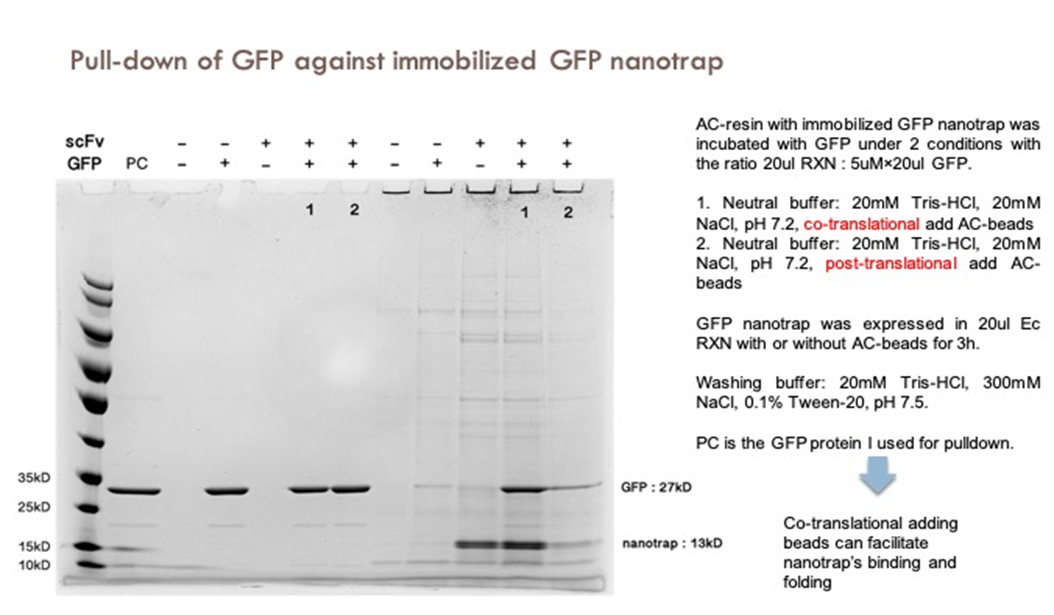 |
| Fig. 21B  SDS-PAGE, Coomassie stained | 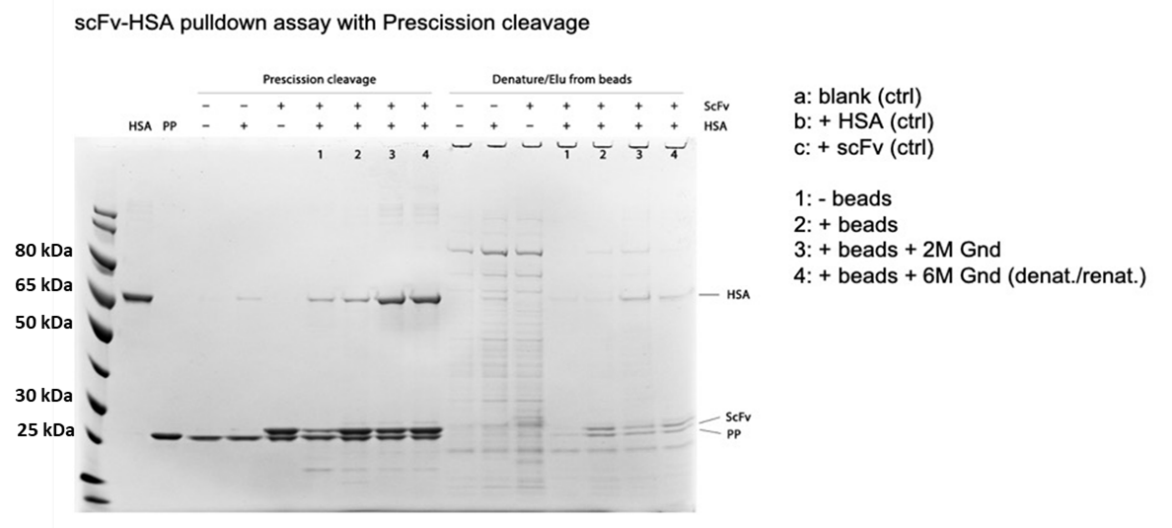 |
| Fig. 21C  SDS-PAGE, Coomassie stained | 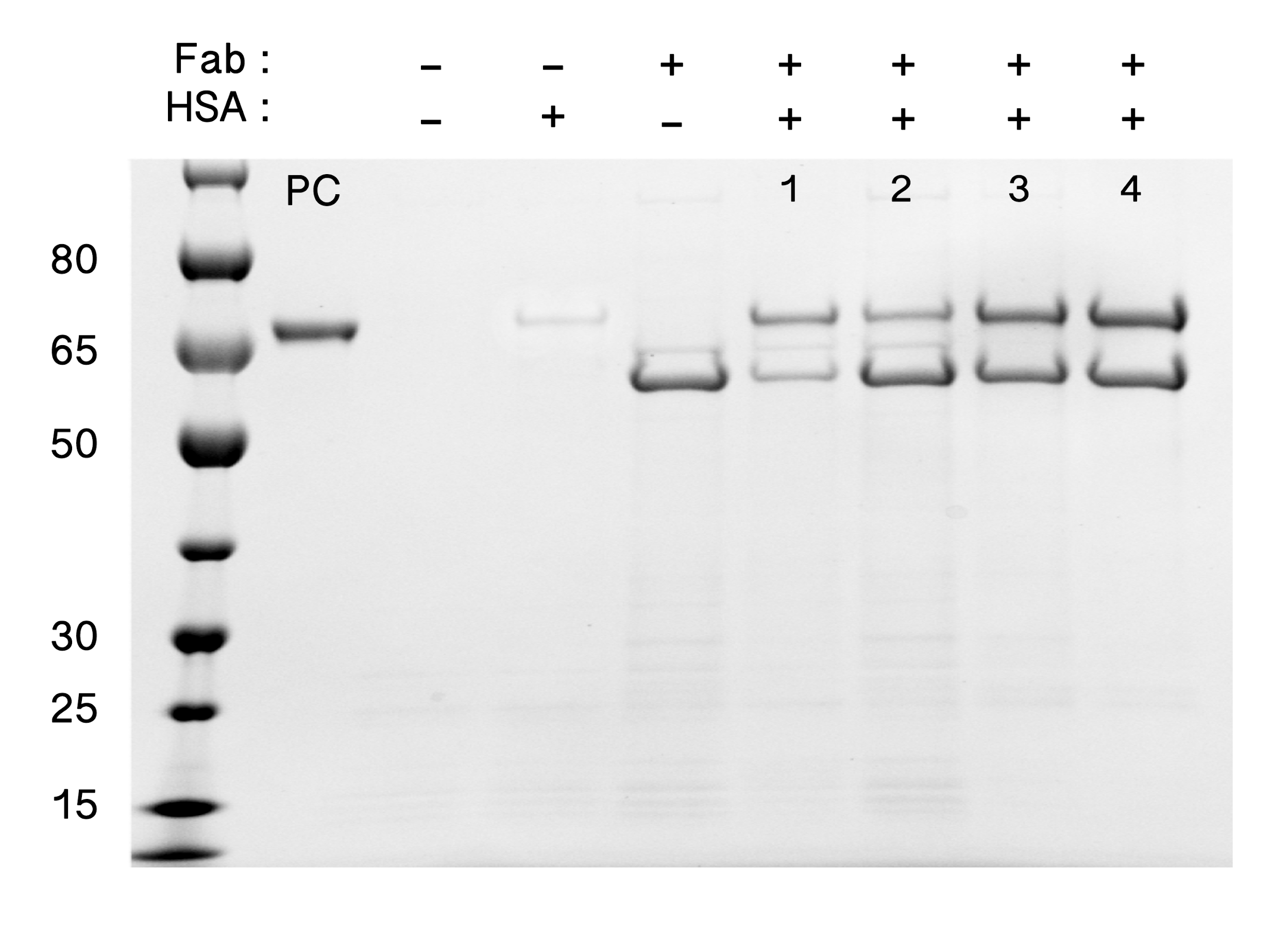 |
| Fig. 22c  SDS-PAGE, Coomassie stained | 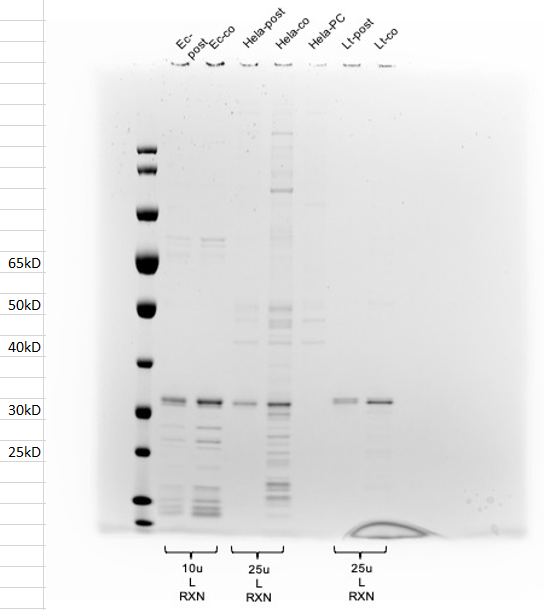 |
| **Supplementary Fig. 24** Uncropped and unprocessed scans of the gels and the western blot. | |

**Supplementary Table 1.** Relationship between size and molar yield for peptides translated in Ec CFS

| name of (poly)peptide | size^a^, aa (+leader) | yield, (μM) | A (size ratio)  peptide/GFP | B (molar ratio)  peptide/GFP | A x B |
| --- | --- | --- | --- | --- | --- |
| SFTI-RGS | 49 | 9.5 | 0.16 | 6.8 | 1.06 |
| KalataB1_RGS | 63 | 8.0 | 0.20 | 5.7 | 1.14 |
| McoTI_RGS | 69 | 6.4 | 0.22 | 4.6 | 1.01 |
| Pn3a_RGS | 72 | 5.0 | 0.23 | 3.6 | 0.81 |
| HT1_RGS | 85 | 7.6 | 0.27 | 5.4 | 1.46 |
| Dc1a_RGS | 106 | 8.9 | 0.34 | 6.4 | 2.16 |
| AA139_RGS | 55 | 4.9 | 0.18 | 3.5 | 0.61^c^ |
| SFTIx1^b^ | 49 | 6.5 | 0.16 | 4.7 | 0.73^c^ |
| SFTIx3^b^ | 77 | 5.2 | 0.25 | 3.7 | 0.91 |
| SFTIx5^b^ | 105 | 4.6 | 0.33 | 3.3 | 1.1 |
| GFP | 315 | 1.4 | 1.0 | 1.0 | 1.0 |

^a^Peptide size corresponds to initially translated sequence including the SITS-leader peptide.

^b^Molar yields for SFTIx1, x2, x3 were obtained based on quantification of monomeric linear SFTI, following their release through the trypsin cleavage of the respective precursors, using calibration of LC-MS signal intensity (cps) to the known amounts of synthetic peptide (Fig. 3B). The concentrations are represented as means of three independent trypsin reactions. For the rest of peptides, the quantification was done by the affinity-clamp assay using the calibration of initial rates of substrate cleavage vs known concentrations of RGS-peptide (Fig. 1a). The peptide concentrations represent the means derived from three AC-assays. The molar yield of GFP was calculated using the calibration of fluorescence intensity vs different protein concentrations (Fig. 16A).

^c^AA139_RGS and ^c^SFTIx1 are likely display underrepresented values due to the autoinhibition of translation reaction by the translated AA139_RGS or due to incomplete cleavage of the precursor SFTI through the trypsin inhibition by the product following RGS-cleavage (Supplementary note 2).

**Supplementary Table 2.** Relative proportions of active GFP and DHFR in GFP-DHFR fusions

|  | Translation in Ec CFS | | | | Translation in LTE | | | |
| --- | --- | --- | --- | --- | --- | --- | --- | --- |
|  | GFP-eDHFR | | GFP-hDHFR^a^ | | GFP-eDHFR | | GFP-hDHFR^a^ | |
|  | co- | post- | co- | post- | co- | post- | co- | post- |
| GFP ± SD (pmol/ml) | 688±96,8 | 331,8±66,2 | 239,7±50,1 | 142,9±34,8 | 411,6 | 389,4 | 116,7 | 41,3 |
| DHFR ± SD (pmol/ml) | 551,2±119,4 | 345,6±49,8 | 39,9±2,14 | 69,4±5,19 | 353,3 | 320,1 | 22,9 | 2,3 |
| GFP/DHFR | 1.2 | 1.0 | 6.0 | 2.0 | 1.2 | 1.2 | 5.1 | 18.6 |

^a^reaction rate for human DHFR (hDHFR) at saturating substrate concentration is limited by the slow product release with only 10% of enzyme fluxing through the reaction pathway^5^, thereby the average picomolar yields calculated for hDHFR using eDHFR calibration plot (Supplementary Fig. 15B) were increased by factor of 10. The table compiles the data from figures 5b, c and 6a, b showing the yields of active GFP and DHFR following translation in Ec CFS and LTE in co- and post-translational formats, respectively.

**Supplementary Table 3.** Summary of expression cassettes used in this study

Designations:

| SD | Shine-Dalgarno motif |
| --- | --- |
| T7 | T7-promoter sequence |
| 5'SITS | 5' untranslated region of species independent translation enhancer = 5'UTR |
| 5'SITS* | 5'SITS + insertion of restriction site |
| 3'SITS | 3' part of species independent translation enhancer encoding for a leader peptide |
| 3'SITS* | 3'SITS with AGT-codon replaced to adjust the codon biase |
| **SD** | Shine-Dalgarno sequence |
| RGS | RGS-tag coding sequence: RGSIDTWV |
| **twin-strep** | Twin-Strep affinity tag coding seuence |
| **POI ORF** | open reading frame of polypeptide of interest |
| start/stop | start and stop codons |
| * | denotes pLTE pasmid comprising altered SITS region |
| Th | Thrombin cleavage site |
| C3 | Prescission protease clevage site |
| TEV | Tobacco Etch Virus protease cleavage site |
| tvmv | tobacco vein mottling virus protease cleavage site |
| myc | myc-tag antigen |

**Constructs based on pLTE:**

**pLTE-backbone:**

| GACGTC**[expression_cassette]**gaattcgtaatcatggtcatagctgtttcctgtgtgaaattgttatccgctcacaattccacacaacatacgagccggaagcataaagtgtaaagcctggggtgcctaatgagtgagctaactcacattaattgcgttgcgctcactgcccgctttccagtcgggaaacctgtcgtgccagctgcattaatgaatcggccaacgcgcggggagaggcggtttgcgtattgggcgctcttccgcttcctcgctcactgactcgctgcgctcggtcgttcggctgcggcgagcggtatcagctcactcaaaggcggtaatacggttatccacagaatcaggggataacgcaggaaagaacatgtgagcaaaaggccagcaaaaggccaggaaccgtaaaaaggccgcgttgctggcgtttttccataggctccgcccccctgacgagcatcacaaaaatcgacgctcaagtcagaggtggcgaaacccgacaggactataaagataccaggcgtttccccctggaagctccctcgtgcgctctcctgttccgaccctgccgcttaccggatacctgtccgcctttctcccttcgggaagcgtggcgctttctcatagctcacgctgtaggtatctcagttcggtgtaggtcgttcgctccaagctgggctgtgtgcacgaaccccccgttcagcccgaccgctgcgccttatccggtaactatcgtcttgagtccaacccggtaagacacgacttatcgccactggcagcagccactggtaacaggattagcagagcgaggtatgtaggcggtgctacagagttcttgaagtggtggcctaactacggctacactagaagaacagtatttggtatctgcgctctgctgaagccagttaccttcggaaaaagagttggtagctcttgatccggcaaacaaaccaccgctggtagcggtggtttttttgtttgcaagcagcagattacgcgcagaaaaaaaggatctcaagaggatcctttgatcttttctacggggtctgacgctcagtggaacgaaaactcacgttaagggattttggtcatgagattatcaaaaaggatcttcacctagatccttttaaattaaaaatgaagttttaaatcaatctaaagtatatatgagtaaacttggtctgacagttaccaatgcttaatcagtgaggcacctatctcagcgatctgtctagttcgttcatccatagttgcctgactccccgtcgtgtagataactacgatacgggagggcttaccatctggccccagtgctgcaatgataccgcgagacccacgctcaccggctccagatttatcagcaataaaccagccagccggaagggccgagcgcagaagtggtcctgcaactttatccgcctccatccagtctattaattgttgccgggaagctagagtaagtagttcgccagttaatagtttgcgcaacgttgttgccattgctacaggcatcgtggtgtcacgctcgtcgtttggtatggcttcattcagctccggttcccaacgatcaaggcgagttacatgatcccccatgttgtgcaaaaaagcggttagctccttcggtcctccgatcgttgtcagaagtaagttggccgcagtgttatcactcatggttatggcagcactgcataattctcttactgtcatgccatccgtaagatgcttttctgtgactggtgagtactcaaccaagtcattctgagaatagtgtatgcggcgaccgagttgctcttgcccggcgtcaatacgggataataccgcgccacatagcagaactttaaaagtgctcatcattggaaaacgttcttcggggcgaaaactctcaaggatcttaccgctgttgagatccagttcgatgtaacccactcgtgcacccaactgatcttcagcatcttttactttcaccagcgtttctgggtgagcaaaaacaggaaggcaaaatgccgcaaaaaagggaataagggcgacacggaaatgttgaatactcatactcttcctttttcaatattattgaagcatttatcagggttattgtctcatgagcggatacatatttgaatgtatttagaaaaataaacaaataggggttccgcgcacatttccccgaaaagtgccacct |
| --- |

**pLTE-RGS**:

| expression cassette = [T7-5'SITS-*start*-3'SITS-tvmv-**RGS***stop-*3'utr-terminator] |
| --- |
| TAATACGACTCACTATAGGGACATCTTAAGTTTATTTTATTTTATTTTATTTTATTTTATTTTATTTTATTTTATTTTATTTTATTTTATTTAAcc*ATG*ACAGTAATGTATAAAGTCTGTAAAGACATTAAACACGTAAGTGAAaccatgGAAACAGTGCGTTTCCAG**CGTGGCTCCATCGACACCTGGGTC***TGATAA*gcggccgccctcctcctcctttcttgttcctttcacgtcgccttctcggttgtagctggcagacgacgagtcttacttttacgtgtacttctctatagatgatgtatgatctctctgcatgcgtgttcgtgcatgtgtccgtgtgttgggtacgcgtggtaccctgcaggaaggaagctgagttggctgctgccaccgctgagcaataactagtaattactagcataaccccttggggcctctaaacgggtcttgagggggttttttgctgaaaggaggacagctgatgattgtcatgcttgccatctgttttcttgcaaggtcagag |

**pLTE-(5'SITS)-RGS**:

| expression cassette = [T7-5'SITS-*start*-tvmv-**RGS***stop-*3'utr-terminator] |
| --- |
| TAATACGACTCACTATAGGGACATCTTAAGTTTATTTTATTTTATTTTATTTTATTTTATTTTATTTTATTTTATTTTATTTTATTTTATTTAAcc*ATG*GAAACAGTGCGTTTCCAG**CGTGGCTCCATCGACACCTGGGTC***TGATAA*gcggccgccctcctcctcctttcttgttcctttcacgtcgccttctcggttgtagctggcagacgacgagtcttacttttacgtgtacttctctatagatgatgtatgatctctctgcatgcgtgttcgtgcatgtgtccgtgtgttgggtacgcgtggtaccctgcaggaaggaagctgagttggctgctgccaccgctgagcaataactagtaattactagcataaccccttggggcctctaaacgggtcttgagggggttttttgctgaaaggaggacagctgatgattgtcatgcttgccatctgttttcttgcaaggtcagag |

**pLTE-(no tvmv)SFTI-RGS**:

| expression cassette = [T7-5'SITS-*start*-3'SITS-**SFTI**_RGS*stop-*3'utr-terminator] |
| --- |
| TAATACGACTCACTATAGGGACATCTTAAGTTTATTTTATTTTATTTTATTTTATTTTATTTTATTTTATTTTATTTTATTTTATTTTATTTAAcc*ATG*ACAGTAATGTATAAAGTCTGTAAAGACATTAAACACGTAAGTGAAaccatg**TCCATTCCTCCGATTTGTTTTCCTGATGGCCGTTGTACCAAA**ggcgcaggcCGTGGCTCCATCGACACCTGGGTC*TGATAA*gcggccgccctcctcctcctttcttgttcctttcacgtcgccttctcggttgtagctggcagacgacgagtcttacttttacgtgtacttctctatagatgatgtatgatctctctgcatgcgtgttcgtgcatgtgtccgtgtgttgggtacgcgtggtaccctgcaggaaggaagctgagttggctgctgccaccgctgagcaataactagtaattactagcataaccccttggggcctctaaacgggtcttgagggggttttttgctgaaaggaggacagctgatgattgtcatgcttgccatctgttttcttgcaaggtcagag |

**pLTE-SFTI-RGS**:

| expression cassette = [T7-5'SITS-*start*-3'SITS-tvmv-**SFTI**_RGS*stop-*3'utr-terminator] |
| --- |
| TAATACGACTCACTATAGGGACATCTTAAGTTTATTTTATTTTATTTTATTTTATTTTATTTTATTTTATTTTATTTTATTTTATTTTATTTAAcc*ATG*ACAGTAATGTATAAAGTCTGTAAAGACATTAAACACGTAAGTGAAaccatgGAAACAGTGCGTTTCCAG**TCCATTCCTCCGATTTGTTTTCCTGATGGCCGTTGTACCAAA**ggcgcaggcCGTGGCTCCATCGACACCTGGGTC*TGATAA*gcggccgccctcctcctcctttcttgttcctttcacgtcgccttctcggttgtagctggcagacgacgagtcttacttttacgtgtacttctctatagatgatgtatgatctctctgcatgcgtgttcgtgcatgtgtccgtgtgttgggtacgcgtggtaccctgcaggaaggaagctgagttggctgctgccaccgctgagcaataactagtaattactagcataaccccttggggcctctaaacgggtcttgagggggttttttgctgaaaggaggacagctgatgattgtcatgcttgccatctgttttcttgcaaggtcagag |

**pLTE-(5'SITS)-SFTI-RGS**:

| expression cassette = [T7-5'SITS-*start*-tvmv-**SFTI**_RGS*stop-*3'utr-terminator] |
| --- |
| TAATACGACTCACTATAGGGACATCTTAAGTTTATTTTATTTTATTTTATTTTATTTTATTTTATTTTATTTTATTTTATTTTATTTTATTTAAcc*ATG*GAAACAGTGCGTTTCCAG**TCCATTCCTCCGATTTGTTTTCCTGATGGCCGTTGTACCAAA**ggcgcaggcCGTGGCTCCATCGACACCTGGGTC*TGATAA*gcggccgccctcctcctcctttcttgttcctttcacgtcgccttctcggttgtagctggcagacgacgagtcttacttttacgtgtacttctctatagatgatgtatgatctctctgcatgcgtgttcgtgcatgtgtccgtgtgttgggtacgcgtggtaccctgcaggaaggaagctgagttggctgctgccaccgctgagcaataactagtaattactagcataaccccttggggcctctaaacgggtcttgagggggttttttgctgaaaggaggacagctgatgattgtcatgcttgccatctgttttcttgcaaggtcagag |

**pLTE-SFTI(R2Q)-RGS**:

| expression cassette = [T7-5'SITS-*start*-3'SITS-tvmv-**SFTI(R2Q)**_RGS*stop-*3'utr-terminator] |
| --- |
| TAATACGACTCACTATAGGGACATCTTAAGTTTATTTTATTTTATTTTATTTTATTTTATTTTATTTTATTTTATTTTATTTTATTTTATTTAAcc*ATG*ACAGTAATGTATAAAGTCTGTAAAGACATTAAACACGTAAGTGAAaccatgGAAACAGTGCGTTTCCAG**TCCATTCCTCCGATTTGTTTTCCTGATGGCCAGTGTACCAAA**ggcgcaggcCGTGGCTCCATCGACACCTGGGTC*TGATAA*gcggccgccctcctcctcctttcttgttcctttcacgtcgccttctcggttgtagctggcagacgacgagtcttacttttacgtgtacttctctatagatgatgtatgatctctctgcatgcgtgttcgtgcatgtgtccgtgtgttgggtacgcgtggtaccctgcaggaaggaagctgagttggctgctgccaccgctgagcaataactagtaattactagcataaccccttggggcctctaaacgggtcttgagggggttttttgctgaaaggaggacagctgatgattgtcatgcttgccatctgttttcttgcaaggtcagag |

**pLTE-SFTI(R2Q)3-RGS**:

| expression cassette = [T7-5'SITS-*start*-3'SITS-tvmv-(**SFTI(R2Q)**)_3__RGS*stop-*3'utr-terminator] |
| --- |
| TAATACGACTCACTATAGGGACATCTTAAGTTTATTTTATTTTATTTTATTTTATTTTATTTTATTTTATTTTATTTTATTTTATTTTATTTAAcc*ATG*ACAGTAATGTATAAAGTCTGTAAAGACATTAAACACGTAAGTGAAaccatgGAAACAGTGCGTTTCCAG**TCCATTCCTCCGATTTGTTTTCCTGATGGCCAGTGTACCAAAAGCATTCCGCCTATTTGCTTTCCGGATGGCCAGTGCACCAAAAGTATCCCACCGATCTGCTTCCCAGACGGTCAATGCACGAAA**ggcgcaggcCGTGGCTCCATCGACACCTGGGTC*TGATAA*gcggccgccctcctcctcctttcttgttcctttcacgtcgccttctcggttgtagctggcagacgacgagtcttacttttacgtgtacttctctatagatgatgtatgatctctctgcatgcgtgttcgtgcatgtgtccgtgtgttgggtacgcgtggtaccctgcaggaaggaagctgagttggctgctgccaccgctgagcaataactagtaattactagcataaccccttggggcctctaaacgggtcttgagggggttttttgctgaaaggaggacagctgatgattgtcatgcttgccatctgttttcttgcaaggtcagag |

**pLTE-SFTI(R2Q)5-RGS**:

| expression cassette = [T7-5'SITS-*start*-3'SITS-tvmv-(**SFTI(R2Q)**)_5__RGS*stop-*3'utr-terminator] |
| --- |
| TAATACGACTCACTATAGGGACATCTTAAGTTTATTTTATTTTATTTTATTTTATTTTATTTTATTTTATTTTATTTTATTTTATTTTATTTAAcc*ATG*ACAGTAATGTATAAAGTCTGTAAAGACATTAAACACGTAAGTGAAaccatgGAAACAGTGCGTTTCCAG**TCCATTCCTCCGATTTGTTTTCCTGATGGCCAGTGTACCAAAAGCATTCCGCCTATTTGCTTTCCGGATGGCCAGTGCACCAAAAGTATCCCACCGATCTGCTTCCCAGACGGTCAATGCACGAAAAGCATCCCGCCAATCTGTTTCCCGGACGGTCAATGTACGAAATCCATTCCGCCGATCTGCTTTCCGGATGGCCAGTGCACCAAA**ggcgcaggcCGTGGCTCCATCGACACCTGGGTC*TGATAA*gcggccgccctcctcctcctttcttgttcctttcacgtcgccttctcggttgtagctggcagacgacgagtcttacttttacgtgtacttctctatagatgatgtatgatctctctgcatgcgtgttcgtgcatgtgtccgtgtgttgggtacgcgtggtaccctgcaggaaggaagctgagttggctgctgccaccgctgagcaataactagtaattactagcataaccccttggggcctctaaacgggtcttgagggggttttttgctgaaaggaggacagctgatgattgtcatgcttgccatctgttttcttgcaaggtcagag |

**pLTE-atg-RGS-atg-GFP**:

| expression cassette = [T7promoter-5'SITS-*start*-3'SITS-rgsORF-stop/start-gfpORF-*stop-*3'utr-terminator] |
| --- |
| TAATACGACTCACTATAGGGACATCTTAAGTTTATTTTATTTTATTTTATTTTATTTTATTTTATTTTATTTTATTTTATTTTATTTTATTTAAcc*ATG*ACAGTAATGTATAAAGTCTGTAAAGACATTAAACACGTAAGTGAAaccatgcggggctccatcgacacctgggtc*TAATGATG*agcaagggcgaggagctgttcaccggggtggtgcccatcctggtcgagctggacggcgacgtaaacggccacaagttcagcgtgtccggcgagggcgagggcgatgccacctacggcaagctgaccctgaagttcatctgcaccaccggcaagctgcccgtgccctggcccaccctcgtgaccaccctgacctacggcgtgcagtgcttcagccgctaccccgaccacatgaagcagcacgacttcttcaagtccgccatgcccgaaggctacgtccaggagcgcaccatcttcttcaaggacgacggcaactacaagacccgcgccgaggtgaagttcgagggcgacaccctggtgaaccgcatcgagctgaagggcatcgacttcaaggaggacggcaacatcctggggcacaagctggagtacaactacaacagccacaacgtctatatcatggccgacaagcagaagaacggcatcaaggtgaacttcaagatccgccacaacatcgaggacggcagcgtgcagctcgccgaccactaccagcagaacacccccatcggcgacggccccgtgctgctgcccgacaaccactacctgagcacccagtccgccctgagcaaagaccccaacgagaagcgcgatcacatggtcctgctggagttcgtgaccgccgccgggatcactctcggcatggacgagctatacaaggagcagaagctgatctcggaggaggatctgcaagcttgtcgacctctagaggatccccggggc*TGATAA*gcggccgccctcctcctcctttcttgttcctttcacgtcgccttctcggttgtagctggcagacgacgagtcttacttttacgtgtacttctctatagatgatgtatgatctctctgcatgcgtgttcgtgcatgtgtccgtgtgttgggtacgcgtggtaccctgcaggaaggaagctgagttggctgctgccaccgctgagcaataactagtaattactagcataaccccttggggcctctaaacgggtcttgagggggttttttgctgaaaggaggacagctgatgattgtcatgcttgccatctgttttcttgcaaggtcagag |

**pLTE-ttt-RGS-atg-GFP**:

| expression cassette = [T7promoter-5'SITS-3'SITS-rgsORF-stop/start-gfpORF-*stop-*3'utr-terminator] |
| --- |
| TAATACGACTCACTATAGGGACATCTTAAGTTTATTTTATTTTATTTTATTTTATTTTATTTTATTTTATTTTATTTTATTTTATTTTATTTAAcc*TTT*ACAGTAATGTATAAAGTCTGTAAAGACATTAAACACGTAAGTGAAaccatgcggggctccatcgacacctgggtc*TAATGATG*agcaagggcgaggagctgttcaccggggtggtgcccatcctggtcgagctggacggcgacgtaaacggccacaagttcagcgtgtccggcgagggcgagggcgatgccacctacggcaagctgaccctgaagttcatctgcaccaccggcaagctgcccgtgccctggcccaccctcgtgaccaccctgacctacggcgtgcagtgcttcagccgctaccccgaccacatgaagcagcacgacttcttcaagtccgccatgcccgaaggctacgtccaggagcgcaccatcttcttcaaggacgacggcaactacaagacccgcgccgaggtgaagttcgagggcgacaccctggtgaaccgcatcgagctgaagggcatcgacttcaaggaggacggcaacatcctggggcacaagctggagtacaactacaacagccacaacgtctatatcatggccgacaagcagaagaacggcatcaaggtgaacttcaagatccgccacaacatcgaggacggcagcgtgcagctcgccgaccactaccagcagaacacccccatcggcgacggccccgtgctgctgcccgacaaccactacctgagcacccagtccgccctgagcaaagaccccaacgagaagcgcgatcacatggtcctgctggagttcgtgaccgccgccgggatcactctcggcatggacgagctatacaaggagcagaagctgatctcggaggaggatctgcaagcttgtcgacctctagaggatccccggggc*TGATAA*gcggccgccctcctcctcctttcttgttcctttcacgtcgccttctcggttgtagctggcagacgacgagtcttacttttacgtgtacttctctatagatgatgtatgatctctctgcatgcgtgttcgtgcatgtgtccgtgtgttgggtacgcgtggtaccctgcaggaaggaagctgagttggctgctgccaccgctgagcaataactagtaattactagcataaccccttggggcctctaaacgggtcttgagggggttttttgctgaaaggaggacagctgatgattgtcatgcttgccatctgttttcttgcaaggtcagag |

**pLTE-wtMcoTI-RGS**:

| expression cassette = [T7-5'SITS-*start*-3'SITS-tvmv-**wtMcoTI**_RGS*stop-*3'utr-terminator] |
| --- |
| TAATACGACTCACTATAGGGACATCTTAAGTTTATTTTATTTTATTTTATTTTATTTTATTTTATTTTATTTTATTTTATTTTATTTTATTTAAcc*ATG*ACAGTAATGTATAAAGTCTGTAAAGACATTAAACACGTAAGTGAAaccatgGAAACAGTGCGTTTCCAG**GGCGGTGTCTGTCCTAAAATTCTGAAAAAATGCCGGCGCGATAGCGATTGCCCGGGCGCGTGCATTTGCCGCGGCAACGGCTATTGCGGCAGCGGCAGCGAT**ggtctgcctCGTGGCTCCATCGACACCTGGGTC*TGATAA*gcggccgccctcctcctcctttcttgttcctttcacgtcgccttctcggttgtagctggcagacgacgagtcttacttttacgtgtacttctctatagatgatgtatgatctctctgcatgcgtgttcgtgcatgtgtccgtgtgttgggtacgcgtggtaccctgcaggaaggaagctgagttggctgctgccaccgctgagcaataactagtaattactagcataaccccttggggcctctaaacgggtcttgagggggttttttgctgaaaggaggacagctgatgattgtcatgcttgccatctgttttcttgcaaggtcagag |

**pLTE-prmMcoTI-RGS**:

| expression cassette = [T7-5'SITS-*start*-3'SITS-tvmv-**prmMcoTI**_RGS*stop-*3'utr-terminator] |
| --- |
| TAATACGACTCACTATAGGGACATCTTAAGTTTATTTTATTTTATTTTATTTTATTTTATTTTATTTTATTTTATTTTATTTTATTTTATTTAAcc*ATG*ACAGTAATGTATAAAGTCTGTAAAGACATTAAACACGTAAGTGAAaccatgGAAACAGTGCGTTTCCAG**ATTCTGAAAAAATGCCGGCGCGATAGCGATTGCCCGGGCGCGTGCATTTGCCGCGGCAACGGCTATTGCGGCAGCGGCAGCGATGGCGGTGTCTGTCCTAAA**ggcgcaggcCGTGGCTCCATCGACACCTGGGTC*TGATAA*gcggccgccctcctcctcctttcttgttcctttcacgtcgccttctcggttgtagctggcagacgacgagtcttacttttacgtgtacttctctatagatgatgtatgatctctctgcatgcgtgttcgtgcatgtgtccgtgtgttgggtacgcgtggtaccctgcaggaaggaagctgagttggctgctgccaccgctgagcaataactagtaattactagcataaccccttggggcctctaaacgggtcttgagggggttttttgctgaaaggaggacagctgatgattgtcatgcttgccatctgttttcttgcaaggtcagag |

**pLTE-kalataB1-RGS**:

| expression cassette = [T7-5'SITS-*start*-3'SITS-tvmv-**-kalataB1**_RGS*stop-*3'utr-terminator] |
| --- |
| TAATACGACTCACTATAGGGACATCTTAAGTTTATTTTATTTTATTTTATTTTATTTTATTTTATTTTATTTTATTTTATTTTATTTTATTTAAcc*ATG*ACAGTAATGTATAAAGTCTGTAAAGACATTAAACACGTAAGTGAAaccatgGAAACAGTGCGTTTCCAG**GGCCTGCCAGTGTGTGGTGAAACCTGCGTGGGTGGCACCTGCAACACCCCTGGCTGTACCTGCAGCTGGCCAGTGTGCACCCGTAAC**ggtctgCGTGGCTCCATCGACACCTGGGTC*TGATAA*gcggccgccctcctcctcctttcttgttcctttcacgtcgccttctcggttgtagctggcagacgacgagtcttacttttacgtgtacttctctatagatgatgtatgatctctctgcatgcgtgttcgtgcatgtgtccgtgtgttgggtacgcgtggtaccctgcaggaaggaagctgagttggctgctgccaccgctgagcaataactagtaattactagcataaccccttggggcctctaaacgggtcttgagggggttttttgctgaaaggaggacagctgatgattgtcatgcttgccatctgttttcttgcaaggtcagag |

**pLTE-AA139-RGS**:

| expression cassette = [T7-5'SITS-*start*-3'SITS-tvmv-**AA139**_RGS*stop-*3'utr-terminator] |
| --- |
| TAATACGACTCACTATAGGGACATCTTAAGTTTATTTTATTTTATTTTATTTTATTTTATTTTATTTTATTTTATTTTATTTTATTTTATTTAAcc*ATG*ACAGTAATGTATAAAGTCTGTAAAGACATTAAACACGTAAGTGAAaccatgGAAACAGTGCGTTTCCAG**GGCTTTTGCTGGTATGTGTGCGCGCGTCGCAATGGTGCGCGTGTGTGCTATCGTCGTTGCAAC**ggtctgCGTGGCTCCATCGACACCTGGGTC*TGATAA*gcggccgccctcctcctcctttcttgttcctttcacgtcgccttctcggttgtagctggcagacgacgagtcttacttttacgtgtacttctctatagatgatgtatgatctctctgcatgcgtgttcgtgcatgtgtccgtgtgttgggtacgcgtggtaccctgcaggaaggaagctgagttggctgctgccaccgctgagcaataactagtaattactagcataaccccttggggcctctaaacgggtcttgagggggttttttgctgaaaggaggacagctgatgattgtcatgcttgccatctgttttcttgcaaggtcagag |

**pLTE-HT1-RGS**:

| expression cassette = [T7-5'SITS-*start*-3'SITS-tvmv-**HT1**_RGS*stop-*3'utr-terminator] |
| --- |
| TAATACGACTCACTATAGGGACATCTTAAGTTTATTTTATTTTATTTTATTTTATTTTATTTTATTTTATTTTATTTTATTTTATTTTATTTAAcc*ATG*ACAGTAATGTATAAAGTCTGTAAAGACATTAAACACGTAAGTGAAaccatgGAAACAGTGCGTTTCCAG**AGCTGCACCAACCCGGGCAAAAAACGCTGCAACGCGAAATGCAGCACCCATTGCGATTGCAAAGATGGCCCGACCCATAACTTTGGCGCGGGCCCGGTGCAGTGCAAAAAATGCACCTATCAGTTTAAAGGCGAAGCGTATTGCAAACAG**ggcgcaggcCGTGGCTCCATCGACACCTGGGTC*TGATAA*gcggccgccctcctcctcctttcttgttcctttcacgtcgccttctcggttgtagctggcagacgacgagtcttacttttacgtgtacttctctatagatgatgtatgatctctctgcatgcgtgttcgtgcatgtgtccgtgtgttgggtacgcgtggtaccctgcaggaaggaagctgagttggctgctgccaccgctgagcaataactagtaattactagcataaccccttggggcctctaaacgggtcttgagggggttttttgctgaaaggaggacagctgatgattgtcatgcttgccatctgttttcttgcaaggtcagag |

**pLTE-Pn3a-RGS**:

| expression cassette = [T7-5'SITS-*start*-3'SITS-tvmv-**Pn3a**_RGS*stop-*3'utr-terminator] |
| --- |
| TAATACGACTCACTATAGGGACATCTTAAGTTTATTTTATTTTATTTTATTTTATTTTATTTTATTTTATTTTATTTTATTTTATTTTATTTAAcc*ATG*ACAGTAATGTATAAAGTCTGTAAAGACATTAAACACGTAAGTGAAaccatgGAAACAGTGCGTTTCCAG**GATTGCCGCTATATGTTTGGCGATTGCGAAAAAGATGAAGATTGCTGCAAACATCTGGGCTGCAAACGCAAAATGAAATATTGCGCGTGGGATTTTACCTTTACC**aacggtctgccaCGTGGCTCCATCGACACCTGGGTC*TGATAA*gcggccgccctcctcctcctttcttgttcctttcacgtcgccttctcggttgtagctggcagacgacgagtcttacttttacgtgtacttctctatagatgatgtatgatctctctgcatgcgtgttcgtgcatgtgtccgtgtgttgggtacgcgtggtaccctgcaggaaggaagctgagttggctgctgccaccgctgagcaataactagtaattactagcataaccccttggggcctctaaacgggtcttgagggggttttttgctgaaaggaggacagctgatgattgtcatgcttgccatctgttttcttgcaaggtcagag |

**pLTE*-Dc1a-RGS**:

| expression cassette = [T7-5'SITS*-*start*-3'SITS*-tvmv-**Dc1a**_TEV_RGS*stop-*3'utr-terminator] |
| --- |
| TAATACGACTCACTATAGGGACATCTTAAGTTTATTTTATTTTATTTTATTTTATTTTATTTTATTTTATTTTATTTTATTTTAattaaTTTTATTTAAcc*ATG*ACAGTAATGTATAAAGTCTGTAAAGACATTAAACACttagGTGAAaccatgGAAACAGTGCGTTTCCAG**TCGGCTAAAGATGGCGATGTGGAAGGACCCGCTGGTTGCAAGAAATATGATGTCGAGTGTGACTCAGGCGAATGTTGCCAGAAACAATACTTATGGTATAAGTGGCGTCCTTTGGATTGCCGCTGTTTGAAGTCAGGCTTCTTCTCGTCGAAATGTGTTTGCCGTGACGTA**ggtggcagtggcaccggtGAGAACCTGTACTTTCAGGGTtcgggctcaggtggcCGTGGCTCCATCGACACCTGGGTC*TGATAA*gcggccgccctcctcctcctttcttgttcctttcacgtcgccttctcggttgtagctggcagacgacgagtcttacttttacgtgtacttctctatagatgatgtatgatctctctgcatgcgtgttcgtgcatgtgtccgtgtgttgggtacgcgtggtaccctgcaggaaggaagctgagttggctgctgccaccgctgagcaataactagtaattactagcataaccccttggggcctctaaacgggtcttgagggggttttttgctgaaaggaggacagctgatgattgtcatgcttgccatctgttttcttgcaaggtcagag |

**pLTE-SFTI(agg,agt)-RGS**:

| expression cassette = [T7-5'SITS-*start*-3'SITS-tvmv-**SFTI(agg,agt)**_RGS*stop-*3'utr-terminator] |
| --- |
| TAATACGACTCACTATAGGGACATCTTAAGTTTATTTTATTTTATTTTATTTTATTTTATTTTATTTTATTTTATTTTATTTTAattaaTTTTATTTAAcc*ATG*ACAGTAATGTATAAAGTCTGTAAAGACATTAAACACttagGTGAAaccatgGAAACAGTGCGTTTCCAG**TCCATTCCTCCGATTAGGTTTCCTGATGGCCGTAGTACCAAA**ggtggcagtggcaccggtGAGAACCTGTACTTTCAGGGTtcgggctcaggtggcCGTGGCTCCATCGACACCTGGGTC*TGATAA*gcggccgccctcctcctcctttcttgttcctttcacgtcgccttctcggttgtagctggcagacgacgagtcttacttttacgtgtacttctctatagatgatgtatgatctctctgcatgcgtgttcgtgcatgtgtccgtgtgttgggtacgcgtggtaccctgcaggaaggaagctgagttggctgctgccaccgctgagcaataactagtaattactagcataaccccttggggcctctaaacgggtcttgagggggttttttgctgaaaggaggacagctgatgattgtcatgcttgccatctgttttcttgcaaggtcagag |

**pLTE*-strep-GFP-RGS**:

| expression cassette = [T7-5'SITS-*start*-3'SITS-tvmv-twin-strep_**GFP**_*3C*_RGS*stop-*3'utr-terminator] |
| --- |
| TAATACGACTCACTATAGGGACATCTTAAGTTTATTTTATTTTATTTTATTTTATTTTATTTTATTTTATTTTATTTTATTTTAattaaTTTTATTTAAcc*ATG*ACAGTAATGTATAAAGTCTGTAAAGACATTAAACACttagGTGAAaccatgGAAACAGTGCGTTTCCAGgcatcatggtcccatcctcaatttgaaaagggcggcggctctggtggtggctccggcgggtcggcctggagccatcctcaattcgagaaatcg**AGCAAGGGCGAGGAGCTGTTCACCGGGGTGGTGCCCATCCTGGTCGAGCTGGACGGCGACGTAAACGGCCACAAGTTCAGCGTGTCCGGCGAGGGCGAGGGCGATGCCACCTACGGCAAGCTGACCCTGAAGTTCATCTGCACCACCGGCAAGCTGCCCGTGCCCTGGCCCACCCTCGTGACCACCCTGACCTACGGCGTGCAGTGCTTCAGCCGCTACCCCGACCACATGAAGCAGCACGACTTCTTCAAGTCCGCCATGCCCGAAGGCTACGTCCAGGAGCGCACCATCTTCTTCAAGGACGACGGCAACTACAAGACCCGCGCCGAGGTGAAGTTCGAGGGCGACACCCTGGTGAACCGCATCGAGCTGAAGGGCATCGACTTCAAGGAGGACGGCAACATCCTGGGGCACAAGCTGGAGTACAACTACAACAGCCACAACGTCTATATCATGGCCGACAAGCAGAAGAACGGCATCAAGGTGAACTTCAAGATCCGCCACAACATCGAGGACGGCAGCGTGCAGCTCGCCGACCACTACCAGCAGAACACCCCCATCGGCGACGGCCCCGTGCTGCTGCCCGACAACCACTACCTGAGCACCCAGTCCGCCCTGAGCAAAGACCCCAACGAGAAGCGCGATCACATGGTCCTGCTGGAGTTCGTGACCGCCGCCGGGATCACTCTCGGCATGGACGAGCTATACAAG**ggtagtggt*TTGGAGGTCTTATTTCAGGGTCCT*ggcagtgggggcCGTGGCTCCATCGACACCTGGGTC*TGATAA*gcggccgccctcctcctcctttcttgttcctttcacgtcgccttctcggttgtagctggcagacgacgagtcttacttttacgtgtacttctctatagatgatgtatgatctctctgcatgcgtgttcgtgcatgtgtccgtgtgttgggtacgcgtggtaccctgcaggaaggaagctgagttggctgctgccaccgctgagcaataactagtaattactagcataaccccttggggcctctaaacgggtcttgagggggttttttgctgaaaggaggacagctgatgattgtcatgcttgccatctgttttcttgcaaggtcagag |

**pLTE*-strep-GFP-eDHFR-RGS**:

| expression cassette = [T7-5'SITS*-*start*-3'SITS*-twin-strep-tvmv-**GFP_***Th***_eDHFR_***3C***_**RGS*stop-*3'utr*-terminator*] |
| --- |
| TAATACGACTCACTATAGGGACATCTTAAGTTTATTTTATTTTATTTTATTTTATTTTATTTTATTTTATTTTATTTTATTTTAattaaTTTTATTTAAcc*ATG*ACAGTAATGTATAAAGTCTGTAAAGACATTAAACACttagGTGAAaccatggcatcaTGGTCACATCCTCAGTTCGAAAAGGGTGGTGGGGCACGTGGCGGTTCGGGCGGGGGTTCGTGGAGTCATCCCCAATTCGAGAAGggatccGAAACAGTGCGTTTCCAG**ATGGTGAGCAAGGGCGAGGAGCTGTTCACCGGGGTGGTGCCCATCCTGGTCGAGCTGGACGGCGACGTAAACGGCCACAAGTTCAGCGTGTCCGGCGAGGGCGAGGGCGATGCCACCTACGGCAAGCTGACCCTGAAGTTCATCTGCACCACCGGCAAGCTGCCCGTGCCCTGGCCCACCCTCGTGACCACCTTCACCTACGGCGTGCAGTGCTTCAGCCGCTACCCCGACCACATGAAGCAGCACGACTTCTTCAAGTCCGCCATGCCCGAAGGCTACGTCCAGGAGCGCACCATCTTCTTCAAGGACGACGGCAACTACAAGACCCGCGCCGAGGTGAAGTTCGAGGGCGACACCCTGGTGAACCGCATCGAGCTGAAGGGCATCGACTTCAAGGAGGACGGCAACATCCTGGGGCACAAGCTGGAGTACAACTACAACAGCCACAACGTCTATATCATGGCCGACAAGCAGAAGAACGGCATCAAGGTGAACTTCAAGATCCGCCACAACATCGAGGACGGCAGCGTGCAGCTCGCCGACCACTACCAGCAGAACACCCCCATCGGCGACGGCCCCGTGCTGCTGCCCGACAACCACTACCTGAGCACCCAGTCCGCCCTGAGCAAAGACCCCAACGAGAAGCGCGATCACATGGTCCTGCTGGAGTTCGTGACCGCCGCCGGGATCACTCTCGGCATGGACGAGCTGTACAAG**ggctccgcgggt*CTGGTGCCACGCGGTAGT***ATGGTTGGTTCGCTAAACTGCATCGTCGCTGTGTCCCAGAACATGGGCATCGGCAAGAACGGGGACCTGCCCTGGCCACCGCTCAGGAATGAATTCAGATATTTCCAGAGAATGACCACAACCTCTTCAGTAGAAGGTAAACAGAATCTGGTGATTATGGGTAAGAAGACCTGGTTCTCCATTCCTGAGAAGAATCGACCTTTAAAGGGTAGAATTAATTTAGTTCTCAGCAGAGAACTCAAGGAACCTCCACAAGGAGCTCATTTTCTTTCCAGAAGTCTAGATGATGCCTTAAAACTTACTGAACAACCAGAATTAGCAAATAAAGTAGACATGGTCTGGATAGTTGGTGGCAGTTCTGTTTATAAGGAAGCCATGAATCACCCAGGCCATCTTAAACTATTTGTGACAAGGATCATGCAAGACTTTGAAAGTGACACGTTTTTTCCAGAAATTGATTTGGAGAAATATAAACTTCTGCCAGAATACCCAGGTGTTCTCTCTGATGTCCAGGAGGAGAAAGGCATTAAGTACAAATTTGAAGTATATGAGAAGAATGAT**agtggt*TTGGAGGTCTTATTTCAGGGTCCT*ggcagtgggggcCGTGGCTCCATCGACACCTGGGTC*TGATAA*gcggccgccctcctcctcctttcttgttcctttcacgtcgccttctcggttgtagctggcagacgacgagtcttacttttacgtgtacttctctatagatgatgtatgatctctctgcatgcgtgttcgtgcatgtgtccgtgtgttgggtacgcgtggtaccctgcaggaaggaagctgagttggctgctgccaccgctgagcaataactagtaattactagcataaccccttggggcctctaaacgggtcttgagggggttttttgctgaaaggaggacagctgatgattgtcatgcttgccatctgttttcttgcaaggtcagag |

**pLTE*-strep-GFP-hDHFR-RGS**:

| expression cassette = [T7-5'SITS*-*start*-3'SITS*-twin-strep-tvmv-**GFP_***Th***_hDHFR_***3C***_**RGS*stop-*3'utr*-terminator*] |
| --- |
| TAATACGACTCACTATAGGGACATCTTAAGTTTATTTTATTTTATTTTATTTTATTTTATTTTATTTTATTTTATTTTATTTTAattaaTTTTATTTAAcc*ATG*ACAGTAATGTATAAAGTCTGTAAAGACATTAAACACttagGTGAAaccatggcatcaTGGTCACATCCTCAGTTCGAAAAGGGTGGTGGGGCACGTGGCGGTTCGGGCGGGGGTTCGTGGAGTCATCCCCAATTCGAGAAGggatccGAAACAGTGCGTTTCCAG**ATGGTGAGCAAGGGCGAGGAGCTGTTCACCGGGGTGGTGCCCATCCTGGTCGAGCTGGACGGCGACGTAAACGGCCACAAGTTCAGCGTGTCCGGCGAGGGCGAGGGCGATGCCACCTACGGCAAGCTGACCCTGAAGTTCATCTGCACCACCGGCAAGCTGCCCGTGCCCTGGCCCACCCTCGTGACCACCTTCACCTACGGCGTGCAGTGCTTCAGCCGCTACCCCGACCACATGAAGCAGCACGACTTCTTCAAGTCCGCCATGCCCGAAGGCTACGTCCAGGAGCGCACCATCTTCTTCAAGGACGACGGCAACTACAAGACCCGCGCCGAGGTGAAGTTCGAGGGCGACACCCTGGTGAACCGCATCGAGCTGAAGGGCATCGACTTCAAGGAGGACGGCAACATCCTGGGGCACAAGCTGGAGTACAACTACAACAGCCACAACGTCTATATCATGGCCGACAAGCAGAAGAACGGCATCAAGGTGAACTTCAAGATCCGCCACAACATCGAGGACGGCAGCGTGCAGCTCGCCGACCACTACCAGCAGAACACCCCCATCGGCGACGGCCCCGTGCTGCTGCCCGACAACCACTACCTGAGCACCCAGTCCGCCCTGAGCAAAGACCCCAACGAGAAGCGCGATCACATGGTCCTGCTGGAGTTCGTGACCGCCGCCGGGATCACTCTCGGCATGGACGAGCTGTACAAG**ggctccgcgggt*CTGGTGCCACGCGGTAGT***ATGGTTGGTTCGCTAAACTGCATCGTCGCTGTGTCCCAGAACATGGGCATCGGCAAGAACGGGGACCTGCCCTGGCCACCGCTCAGGAATGAATTCAGATATTTCCAGAGAATGACCACAACCTCTTCAGTAGAAGGTAAACAGAATCTGGTGATTATGGGTAAGAAGACCTGGTTCTCCATTCCTGAGAAGAATCGACCTTTAAAGGGTAGAATTAATTTAGTTCTCAGCAGAGAACTCAAGGAACCTCCACAAGGAGCTCATTTTCTTTCCAGAAGTCTAGATGATGCCTTAAAACTTACTGAACAACCAGAATTAGCAAATAAAGTAGACATGGTCTGGATAGTTGGTGGCAGTTCTGTTTATAAGGAAGCCATGAATCACCCAGGCCATCTTAAACTATTTGTGACAAGGATCATGCAAGACTTTGAAAGTGACACGTTTTTTCCAGAAATTGATTTGGAGAAATATAAACTTCTGCCAGAATACCCAGGTGTTCTCTCTGATGTCCAGGAGGAGAAAGGCATTAAGTACAAATTTGAAGTATATGAGAAGAATGAT**agtggt*TTGGAGGTCTTATTTCAGGGTCCT*ggcagtgggggcCGTGGCTCCATCGACACCTGGGTC*TGATAA*gcggccgccctcctcctcctttcttgttcctttcacgtcgccttctcggttgtagctggcagacgacgagtcttacttttacgtgtacttctctatagatgatgtatgatctctctgcatgcgtgttcgtgcatgtgtccgtgtgttgggtacgcgtggtaccctgcaggaaggaagctgagttggctgctgccaccgctgagcaataactagtaattactagcataaccccttggggcctctaaacgggtcttgagggggttttttgctgaaaggaggacagctgatgattgtcatgcttgccatctgttttcttgcaaggtcagag |

**pLTE*-strep-hDHFR-GFP-RGS**:

| expression cassette = [T7-5'SITS*-*start*-3'SITS*-twin-strep-tvmv-**hDHFR_***Th***_GFP_***3C***_**RGS*stop-*3'utr*-terminator*] |
| --- |
| TAATACGACTCACTATAGGGACATCTTAAGTTTATTTTATTTTATTTTATTTTATTTTATTTTATTTTATTTTATTTTATTTTAattaaTTTTATTTAAcc*ATG*ACAGTAATGTATAAAGTCTGTAAAGACATTAAACACttagGTGAAaccatggcatcaTGGTCACATCCTCAGTTCGAAAAGGGTGGTGGGGCACGTGGCGGTTCGGGCGGGGGTTCGTGGAGTCATCCCCAATTCGAGAAGggatccGAAACAGTGCGTTTCCAG**ATGGTTGGTTCGCTAAACTGCATCGTCGCTGTGTCCCAGAACATGGGCATCGGCAAGAACGGGGACCTGCCCTGGCCACCGCTCAGGAATGAATTCAGATATTTCCAGAGAATGACCACAACCTCTTCAGTAGAAGGTAAACAGAATCTGGTGATTATGGGTAAGAAGACCTGGTTCTCCATTCCTGAGAAGAATCGACCTTTAAAGGGTAGAATTAATTTAGTTCTCAGCAGAGAACTCAAGGAACCTCCACAAGGAGCTCATTTTCTTTCCAGAAGTCTAGATGATGCCTTAAAACTTACTGAACAACCAGAATTAGCAAATAAAGTAGACATGGTCTGGATAGTTGGTGGCAGTTCTGTTTATAAGGAAGCCATGAATCACCCAGGCCATCTTAAACTATTTGTGACAAGGATCATGCAAGACTTTGAAAGTGACACGTTTTTTCCAGAAATTGATTTGGAGAAATATAAACTTCTGCCAGAATACCCAGGTGTTCTCTCTGATGTCCAGGAGGAGAAAGGCATTAAGTACAAATTTGAAGTATATGAGAAGAATGAT**ggctccgcgggt*CTGGTGCCACGCGGTAGT***ATGGTGAGCAAGGGCGAGGAGCTGTTCACCGGGGTGGTGCCCATCCTGGTCGAGCTGGACGGCGACGTAAACGGCCACAAGTTCAGCGTGTCCGGCGAGGGCGAGGGCGATGCCACCTACGGCAAGCTGACCCTGAAGTTCATCTGCACCACCGGCAAGCTGCCCGTGCCCTGGCCCACCCTCGTGACCACCTTCACCTACGGCGTGCAGTGCTTCAGCCGCTACCCCGACCACATGAAGCAGCACGACTTCTTCAAGTCCGCCATGCCCGAAGGCTACGTCCAGGAGCGCACCATCTTCTTCAAGGACGACGGCAACTACAAGACCCGCGCCGAGGTGAAGTTCGAGGGCGACACCCTGGTGAACCGCATCGAGCTGAAGGGCATCGACTTCAAGGAGGACGGCAACATCCTGGGGCACAAGCTGGAGTACAACTACAACAGCCACAACGTCTATATCATGGCCGACAAGCAGAAGAACGGCATCAAGGTGAACTTCAAGATCCGCCACAACATCGAGGACGGCAGCGTGCAGCTCGCCGACCACTACCAGCAGAACACCCCCATCGGCGACGGCCCCGTGCTGCTGCCCGACAACCACTACCTGAGCACCCAGTCCGCCCTGAGCAAAGACCCCAACGAGAAGCGCGATCACATGGTCCTGCTGGAGTTCGTGACCGCCGCCGGGATCACTCTCGGCATGGACGAGCTGTACAAG**agtggt*TTGGAGGTCTTATTTCAGGGTCCT*ggcagtgggggcCGTGGCTCCATCGACACCTGGGTC*TGATAA*gcggccgccctcctcctcctttcttgttcctttcacgtcgccttctcggttgtagctggcagacgacgagtcttacttttacgtgtacttctctatagatgatgtatgatctctctgcatgcgtgttcgtgcatgtgtccgtgtgttgggtacgcgtggtaccctgcaggaaggaagctgagttggctgctgccaccgctgagcaataactagtaattactagcataaccccttggggcctctaaacgggtcttgagggggttttttgctgaaaggaggacagctgatgattgtcatgcttgccatctgttttcttgcaaggtcagag |

**pLTE*-strep-GFP-hDHFR(P66A)-RGS**:

| expression cassette = [T7-5'SITS*-*start*-3'SITS*-twin-strep-tvmv-**GFP_***Th***_hDHFR(P66A)_***3C***_**RGS*stop-*3'utr*-terminator*] |
| --- |
| TAATACGACTCACTATAGGGACATCTTAAGTTTATTTTATTTTATTTTATTTTATTTTATTTTATTTTATTTTATTTTATTTTAattaaTTTTATTTAAcc*ATG*ACAGTAATGTATAAAGTCTGTAAAGACATTAAACACttagGTGAAaccatggcatcaTGGTCACATCCTCAGTTCGAAAAGGGTGGTGGGGCACGTGGCGGTTCGGGCGGGGGTTCGTGGAGTCATCCCCAATTCGAGAAGggatccGAAACAGTGCGTTTCCAG**ATGGTGAGCAAGGGCGAGGAGCTGTTCACCGGGGTGGTGCCCATCCTGGTCGAGCTGGACGGCGACGTAAACGGCCACAAGTTCAGCGTGTCCGGCGAGGGCGAGGGCGATGCCACCTACGGCAAGCTGACCCTGAAGTTCATCTGCACCACCGGCAAGCTGCCCGTGCCCTGGCCCACCCTCGTGACCACCTTCACCTACGGCGTGCAGTGCTTCAGCCGCTACCCCGACCACATGAAGCAGCACGACTTCTTCAAGTCCGCCATGCCCGAAGGCTACGTCCAGGAGCGCACCATCTTCTTCAAGGACGACGGCAACTACAAGACCCGCGCCGAGGTGAAGTTCGAGGGCGACACCCTGGTGAACCGCATCGAGCTGAAGGGCATCGACTTCAAGGAGGACGGCAACATCCTGGGGCACAAGCTGGAGTACAACTACAACAGCCACAACGTCTATATCATGGCCGACAAGCAGAAGAACGGCATCAAGGTGAACTTCAAGATCCGCCACAACATCGAGGACGGCAGCGTGCAGCTCGCCGACCACTACCAGCAGAACACCCCCATCGGCGACGGCCCCGTGCTGCTGCCCGACAACCACTACCTGAGCACCCAGTCCGCCCTGAGCAAAGACCCCAACGAGAAGCGCGATCACATGGTCCTGCTGGAGTTCGTGACCGCCGCCGGGATCACTCTCGGCATGGACGAGCTGTACAAG**ggctccgcgggt*CTGGTGCCACGCGGTAGT***ATGGTTGGTTCGCTAAACTGCATCGTCGCTGTGTCCCAGAACATGGGCATCGGCAAGAACGGGGACCTGCCCTGGCCACCGCTCAGGAATGAATTCAGATATTTCCAGAGAATGACCACAACCTCTTCAGTAGAAGGTAAACAGAATCTGGTGATTATGGGTAAGAAGACCTGGTTCTCCATTCCTGAGAAGAATCGAGCTTTAAAGGGTAGAATTAATTTAGTTCTCAGCAGAGAACTCAAGGAACCTCCACAAGGAGCTCATTTTCTTTCCAGAAGTCTAGATGATGCCTTAAAACTTACTGAACAACCAGAATTAGCAAATAAAGTAGACATGGTCTGGATAGTTGGTGGCAGTTCTGTTTATAAGGAAGCCATGAATCACCCAGGCCATCTTAAACTATTTGTGACAAGGATCATGCAAGACTTTGAAAGTGACACGTTTTTTCCAGAAATTGATTTGGAGAAATATAAACTTCTGCCAGAATACCCAGGTGTTCTCTCTGATGTCCAGGAGGAGAAAGGCATTAAGTACAAATTTGAAGTATATGAGAAGAATGAT**agtggt*TTGGAGGTCTTATTTCAGGGTCCT*ggcagtgggggcCGTGGCTCCATCGACACCTGGGTC*TGATAA*gcggccgccctcctcctcctttcttgttcctttcacgtcgccttctcggttgtagctggcagacgacgagtcttacttttacgtgtacttctctatagatgatgtatgatctctctgcatgcgtgttcgtgcatgtgtccgtgtgttgggtacgcgtggtaccctgcaggaaggaagctgagttggctgctgccaccgctgagcaataactagtaattactagcataaccccttggggcctctaaacgggtcttgagggggttttttgctgaaaggaggacagctgatgattgtcatgcttgccatctgttttcttgcaaggtcagag |

**pLTE-GFP**:

| expression cassette = [T7-5'SITS-*start*-3'SITS-**GFP_myc**_MCS**-***stop-*3'utr-terminator] |
| --- |
| TAATACGACTCACTATAGGGACATCTTAAGTTTATTTTATTTTATTTTATTTTATTTTATTTTATTTTATTTTATTTTATTTTATTTTATTTAAcc*ATG*ACAGTAATGTATAAAGTCTGTAAAGACATTAAACACGTAAGTGAAaccatggagatctcg**AGCAAGGGCGAGGAGCTGTTCACCGGGGTGGTGCCCATCCTGGTCGAGCTGGACGGCGACGTAAACGGCCACAAGTTCAGCGTGTCCGGCGAGGGCGAGGGCGATGCCACCTACGGCAAGCTGACCCTGAAGTTCATCTGCACCACCGGCAAGCTGCCCGTGCCCTGGCCCACCCTCGTGACCACCCTGACCTACGGCGTGCAGTGCTTCAGCCGCTACCCCGACCACATGAAGCAGCACGACTTCTTCAAGTCCGCCATGCCCGAAGGCTACGTCCAGGAGCGCACCATCTTCTTCAAGGACGACGGCAACTACAAGACCCGCGCCGAGGTGAAGTTCGAGGGCGACACCCTGGTGAACCGCATCGAGCTGAAGGGCATCGACTTCAAGGAGGACGGCAACATCCTGGGGCACAAGCTGGAGTACAACTACAACAGCCACAACGTCTATATCATGGCCGACAAGCAGAAGAACGGCATCAAGGTGAACTTCAAGATCCGCCACAACATCGAGGACGGCAGCGTGCAGCTCGCCGACCACTACCAGCAGAACACCCCCATCGGCGACGGCCCCGTGCTGCTGCCCGACAACCACTACCTGAGCACCCAGTCCGCCCTGAGCAAAGACCCCAACGAGAAGCGCGATCACATGGTCCTGCTGGAGTTCGTGACCGCCGCCGGGATCACTCTCGGCATGGACGAGCTATACAAGgagcagaagctgatctcggaggaggatctg**CAAGCTTGTCGACCTCTAGAGGATCCCCGGGGC*TAA*gcggccgccctcctcctcctttcttgttcctttcacgtcgccttctcggttgtagctggcagacgacgagtcttacttttacgtgtacttctctatagatgatgtatgatctctctgcatgcgtgttcgtgcatgtgtccgtgtgttgggtacgcgtggtaccctgcaggaaggaagctgagttggctgctgccaccgctgagcaataactagtaattactagcataaccccttggggcctctaaacgggtcttgagggggttttttgctgaaaggaggacagctgatgattgtcatgcttgccatctgttttcttgcaaggtcagag |

**pLTE-GFP-RGS**:

| expression cassette = [T7-5'SITS-*start*-3'SITS-**GFP_myc**_*3C***-**RGS*stop-*3'utr-terminator] |
| --- |
| TAATACGACTCACTATAGGGACATCTTAAGTTTATTTTATTTTATTTTATTTTATTTTATTTTATTTTATTTTATTTTATTTTATTTTATTTAAcc*ATG*ACAGTAATGTATAAAGTCTGTAAAGACATTAAACACGTAAGTGAAaccatggagatctcg**AGCAAGGGCGAGGAGCTGTTCACCGGGGTGGTGCCCATCCTGGTCGAGCTGGACGGCGACGTAAACGGCCACAAGTTCAGCGTGTCCGGCGAGGGCGAGGGCGATGCCACCTACGGCAAGCTGACCCTGAAGTTCATCTGCACCACCGGCAAGCTGCCCGTGCCCTGGCCCACCCTCGTGACCACCCTGACCTACGGCGTGCAGTGCTTCAGCCGCTACCCCGACCACATGAAGCAGCACGACTTCTTCAAGTCCGCCATGCCCGAAGGCTACGTCCAGGAGCGCACCATCTTCTTCAAGGACGACGGCAACTACAAGACCCGCGCCGAGGTGAAGTTCGAGGGCGACACCCTGGTGAACCGCATCGAGCTGAAGGGCATCGACTTCAAGGAGGACGGCAACATCCTGGGGCACAAGCTGGAGTACAACTACAACAGCCACAACGTCTATATCATGGCCGACAAGCAGAAGAACGGCATCAAGGTGAACTTCAAGATCCGCCACAACATCGAGGACGGCAGCGTGCAGCTCGCCGACCACTACCAGCAGAACACCCCCATCGGCGACGGCCCCGTGCTGCTGCCCGACAACCACTACCTGAGCACCCAGTCCGCCCTGAGCAAAGACCCCAACGAGAAGCGCGATCACATGGTCCTGCTGGAGTTCGTGACCGCCGCCGGGATCACTCTCGGCATGGACGAGCTATACAAGgagcagaagctgatctcggaggaggatctg**accggt*CTGGAAGTGCTGTTTCAGGGTCCG*accggtactggcaccggtCGTGGCAGCATTGATACCTGGGTG*TAA*gcggccgccctcctcctcctttcttgttcctttcacgtcgccttctcggttgtagctggcagacgacgagtcttacttttacgtgtacttctctatagatgatgtatgatctctctgcatgcgtgttcgtgcatgtgtccgtgtgttgggtacgcgtggtaccctgcaggaaggaagctgagttggctgctgccaccgctgagcaataactagtaattactagcataaccccttggggcctctaaacgggtcttgagggggttttttgctgaaaggaggacagctgatgattgtcatgcttgccatctgttttcttgcaaggtcagag |

**pLTE-αGFP-Vhh-RGS**:

| expression cassette = [T7-5'SITS-*start*-3'SITS-tvmv-**VHH-RGS***stop-*3'utr-terminator] |
| --- |
| TAATACGACTCACTATAGGGACATCTTAAGTTTATTTTATTTTATTTTATTTTATTTTATTTTATTTTATTTTATTTTATTTTATTTTATTTAAcc*ATG*ACAGTAATGTATAAAGTCTGTAAAGACATTAAACACGTAAGTGAAaccatgGAAACAGTGCGTTTCCAG**ATGCAGGTTCAACTGGTGGAAAGCGGCGGTGCTCTGGTACAACCGGGCGGTAGTCTGCGCCTGAGCTGTGCCGCAAGCGGTTTCCCAGTCAACCGCTACTCTATGCGTTGGTATCGCCAGGCGCCTGGTAAAGAACGTGAATGGGTTGCCGGCATGAGCAGTGCGGGCGATCGTTCTAGTTACGAGGACTCTGTTAAAGGTCGTTTTACAATTAGCCGTGATGATGCGCGCAATACCGTGTATCTGCAAATGAACAGTCTGAAGCCGGAGGACACCGCAGTATATTATTGCAATGTCAACGTGGGGTTTGAATATTGGGGCCAGGGGACTCAGGTGACGGTGAGCTCT**ggcagcggcctggaagtgctgtttcagggcccgggcagcggcggt**CGTGGCTCCATCGACACCTGGGTC***TGATAA*gcggccgccctcctcctcctttcttgttcctttcacgtcgccttctcggttgtagctggcagacgacgagtcttacttttacgtgtacttctctatagatgatgtatgatctctctgcatgcgtgttcgtgcatgtgtccgtgtgttgggtacgcgtggtaccctgcaggaaggaagctgagttggctgctgccaccgctgagcaataactagtaattactagcataaccccttggggcctctaaacgggtcttgagggggttttttgctgaaaggaggacagctgatgattgtcatgcttgccatctgttttcttgcaaggtcagag |

**pLTE-αHSA(**[**5fuo**](about:blank)**)-scFv-RGS**:

| expression cassette = [T7-5'SITS-*start*-3'SITS-tvmv-**VH_VL_RGS***stop-*3'utr-terminator] |
| --- |
| TAATACGACTCACTATAGGGACATCTTAAGTTTATTTTATTTTATTTTATTTTATTTTATTTTATTTTATTTTATTTTATTTTATTTTATTTAAcc*ATG*ACAGTAATGTATAAAGTCTGTAAAGACATTAAACACGTAAGTGAAaccatgGAAACAGTGCGTTTCCAG**AGCACGAAAGGTCCTAGCGTCTTCCCCCTGGCACCAAGTAGCAAATCTACCAGTGGAGGCACTGCGGCGCTGGGATGTTTAGTGAAAGACTATTTCCCGGAGCCCGTAACTGTGTCCTGGAATTCAGGAGCTCTTACTTCTGGCGTACATACCTTTCCGGCTGTCCTTCAGTCCTCCGGTTTGTATAGTTTATCTAGCGTCGTAACCGTTCCGAGCAGTAGTCTGGGCACGCAGACGTACATTTGCAACGTGAATCATAAGCCGTCCAATACTAAGGTTGACAAAAAGGTAGAACCGAAGTCGTGTGACAAG**ggcggcagtggaggatcaggtagcggaagcggtggtagcggaggg**CGCACTGTTGCTGCGCCATCGGTATTTATTTTCCCTCCGAGCGATGAGCAGTTAAAGAGCGGTACTGCTTCAGTCGTCTGTCTGCTGAACAATTTCTATCCTCGTGAAGCAAAGGTCCAATGGAAAGTTGATAACGCTTTACAAAGCGGTAACAGCCAGGAATCAGTAACTGAACAGGACAGCAAAGACAGCACGTATAGCTTGTCAAGCACACTGACTTTGTCGAAAGCGGACTATGAGAAACACAAAGTCTACGCTTGCGAGGTAACACACCAAGGACTGTCCTCTCCAGTTACCAAGTCTTTCAACCGTGGAGAATGC**ggtagtggtttggaggtcttatttcagggtcctggcagtgggggc**CGTGGCTCCATCGACACCTGGGTC***TGATAA*gcggccgccctcctcctcctttcttgttcctttcacgtcgccttctcggttgtagctggcagacgacgagtcttacttttacgtgtacttctctatagatgatgtatgatctctctgcatgcgtgttcgtgcatgtgtccgtgtgttgggtacgcgtggtaccctgcaggaaggaagctgagttggctgctgccaccgctgagcaataactagtaattactagcataaccccttggggcctctaaacgggtcttgagggggttttttgctgaaaggaggacagctgatgattgtcatgcttgccatctgttttcttgcaaggtcagag |

**pLTE-αHSA(**[**5fuo**](about:blank)**)-Fab-RGS**:

| expression cassette = [T7-5'SITS-*start*-3'SITS-tvmv-**VHCH_VLCL_RGS***stop-*3'utr-terminator] |
| --- |
| TAATACGACTCACTATAGGGACATCTTAAGTTTATTTTATTTTATTTTATTTTATTTTATTTTATTTTATTTTATTTTATTTTATTTTATTTAAcc*ATG*ACAGTAATGTATAAAGTCTGTAAAGACATTAAACACGTAAGTGAAaccatgGAAACAGTGCGTTTCCAG**GAGGTCCAACTTTTGGAATCCGGAGGAGGGCTTGTTCAACCCGGAGGCTCCTTGCGCTTGTCGTGCGCCGTGAGTGGAATCGACTTATCTAATTACGCAATCAATTGGGTGCGTCAGGCCCCGGGGAAGGGTTTGGAATGGATCGGCATTATCTGGGCATCGGGAACCACCTTCTATGCTACCTGGGCCAAGGGGCGCTTCACCATTTCGCGTGATAACTCCAAGAACACTGTGTATTTGCAAATGAATTCGCTGCGCGCTGAAGATACGGCGGTTTATTATTGCGCGCGCACAGTTCCTGGCTACTCAACAGCGCCGTATTTCGACCTGTGGGGCCAGGGAACACTGGTTACCGTATCCTCAGCATCGACAAAGGGTCCGTCCGTCTTTCCCTTGGCACCCTCATCGAAGTCTACCTCCGGGGGCACTGCTGCACTGGGCTGTTTAGTTAAGGATTATTTTCCCGAACCTGTCACAGTCTCCTGGAACTCGGGCGCTTTAACGTCGGGAGTTCACACCTTCCCGGCCGTGTTACAGTCCTCGGGATTGTATTCTTTGTCATCGGTAGTAACAGTACCCTCAAGCTCACTGGGTACTCAAACTTACATTTGTAATGTGAACCATAAACCATCGAACACTAAAGTTGATAAGAAGGTCGAACCAAAAAGTTGCGACAAAACA**ggagggagtggcgggagttcgggtggatcaggaggttctggaggctcttcgggaggatcgggaagcggaagtggatctggttcgggtagcgggtccggaggcagtggagggtcaggtgga**GATATCCAAATGACCCAGTCTCCCAGTTCAGTTTCTGCGAGCGTGGGTGATCGTGTCACCATTACCTGTCAAAGTTCTCCCAGTGTGTGGTCCAATTTTCTTTCCTGGTATCAACAGAAACCCGGTAAGGCTCCAAAATTGTTGATCTATGAGGCTAGTAAATTAACGTCTGGCGTTCCTTCGCGCTTCAGCGGAAGTGGATCGGGCACCGATTTTACTTTAACCATCTCAAGCCTTCAACCGGAAGACTTCGCTACATATTACTGCGGAGGTGGCTATTCGTCGATCAGTGACACGACCTTTGGTGGTGGGACCAAAGTTGAAATTAAGCGTACTGTTGCTGCGCCCTCAGTATTTATCTTTCCCCCCTCAGACGAGCAGTTAAAATCAGGCACAGCTTCTGTGGTCTGTCTTCTTAATAATTTCTACCCCCGTGAGGCTAAGGTACAGTGGAAGGTGGACAATGCATTGCAGTCCGGTAACAGCCAGGAGAGTGTGACAGAGCAAGACTCGAAGGACTCTACCTACAGCCTTTCTAGCACATTGACGCTTAGCAAAGCAGATTATGAAAAACACAAAGTCTACGCATGTGAGGTGACTCACCAGGGATTAAGCTCTCCCGTCACGAAATCATTCAACCGTGGTGAGTGT**ggtagtggtttggaggtcttatttcagggtcctggcagtgggggc**CGTGGCTCCATCGACACCTGGGTC***TGATAA*gcggccgccctcctcctcctttcttgttcctttcacgtcgccttctcggttgtagctggcagacgacgagtcttacttttacgtgtacttctctatagatgatgtatgatctctctgcatgcgtgttcgtgcatgtgtccgtgtgttgggtacgcgtggtaccctgcaggaaggaagctgagttggctgctgccaccgctgagcaataactagtaattactagcataaccccttggggcctctaaacgggtcttgagggggttttttgctgaaaggaggacagctgatgattgtcatgcttgccatctgttttcttgcaaggtcagag |

**pLTE-αHSA(4D2 patent)-scFv-RGS**:

| expression cassette = [T7-5'SITS-*start*-3'SITS-tvmv-**VH_VL_RGS***stop-*3'utr-terminator] |
| --- |
| TAATACGACTCACTATAGGGACATCTTAAGTTTATTTTATTTTATTTTATTTTATTTTATTTTATTTTATTTTATTTTATTTTATTTTATTTAAcc*ATG*ACAGTAATGTATAAAGTCTGTAAAGACATTAAACACGTAAGTGAAaccatgGAAACAGTGCGTTTCCAG**GTGCAGCTGGTGCAGAGTGGTGCAGAAGTTAAAAAACCGGGCGCAAGTGTTAAAGTTAGTTGTAAAGCAAGCGGTTATACCTTTACCAGTTATGGCATTAGTTGGGTGCGTCAGGCACCGGGTCAGGGTCTGGAATGGATGGGCTGGATTAGTGCCTATAATGGTAATACCAATTACGCACAGAAATTTCAGGGTCGCGTTACCATGACCGAAGATACCAGTACCGATACCGCATATATGGAACTGAGCAGCCTGCGTAGCGAAGATACCGCCGTGTATTATTGTGCCACCGAAGGCTATAGTGGCTATGATCTGTATGCCTTTGATATTTGGGGTCAGGGCACCATGGTTACAGTGAGCGGTGGCAGCGGTGGTGGCAGTGGTGGCGGTAGTGGTGGTGGCAGCGGCGGTGGTGTTGTTATGACCCAGAGTCCGCTGTCACTGCCGGTTACACCGGGCGAACCGGCCAGTATTAGCTGCCGCAGTAGTCAGAGTCTGCTGCATAGTAATGGTTATAATTATCTGAACTGGTTCCAGCAGCGTCCGGGCCAGAGCCCGCGTAGACTGATCTATAAAGTGAGTAATCGTGATAGCGGCGTGCCGGATCGCTTTAGCGGCAGCGGTAGTGGTACAGATTTTACCCTGAAAATTAGCCGTGTGGAAGCAGAAGATGTTGGCGTTTATTATTGCATGCAGGGCACCCATTGGCCGCCGATTACCTTTGGTCAGGGTACACGTCTGGAAACC**ggtagtggtttggaggtcttatttcagggtcctggcagtgggggc**CGTGGCTCCATCGACACCTGGGTC***TGATAA*gcggccgccctcctcctcctttcttgttcctttcacgtcgccttctcggttgtagctggcagacgacgagtcttacttttacgtgtacttctctatagatgatgtatgatctctctgcatgcgtgttcgtgcatgtgtccgtgtgttgggtacgcgtggtaccctgcaggaaggaagctgagttggctgctgccaccgctgagcaataactagtaattactagcataaccccttggggcctctaaacgggtcttgagggggttttttgctgaaaggaggacagctgatgattgtcatgcttgccatctgttttcttgcaaggtcagag |

**pLTE-αIFNα-1b(**[**3ux9**](about:blank)**)-scFv-RGS**:

| expression cassette = [T7-5'SITS-*start*-3'SITS-tvmv-**VH_VL_RGS***stop-*3'utr-terminator] |
| --- |
| TAATACGACTCACTATAGGGACATCTTAAGTTTATTTTATTTTATTTTATTTTATTTTATTTTATTTTATTTTATTTTATTTTATTTTATTTAAcc*ATG*ACAGTAATGTATAAAGTCTGTAAAGACATTAAACACGTAAGTGAAaccatgGAAACAGTGCGTTTCCAG**GTTCAGCTGGTTGAAAGCGGTGGCGGTCTGGTTCAGCCGGGCGGTAGTCTGCGTCTGAGCTGTGCAGCCAGTGGCTTTACCTTTAGTAGTTATGCCATGAGTTGGGTGCGCCAGGCCCCTGGTAAAGGCCTGGAATGGGTGAGTGCAATTAGTGGTAGCGGTGGCAGCACCTATTATGCAGATAGTGTGAAAGGTCGCTTTACCATTAGCCGTGATAATAGTAAAAACACCCTGTATCTGCAGATGAATAGCCTGCGTGCAGAAGATACCGCAGTGTATTATTGCGCCCGTTATATTGATTTTGGCGATCATATGGATTTCTGGGGTCAGGGCACCCTGGTTACAGTGAGTGGTGGTAGCGGTGGTGGTAGCGGCGGTGGCAGTGGCGGTGGTAGCGGAGGTGGCATTGTTCTGACCCAGCCGCCGAGCGTTAGTGGTGCACCGGGCCAGCGCGTTACCATTAGTTGCAGCGGCAGTAGTAGTAATATTGGCAGCAATTATGTGAGCTGGTATCAGCAGCTGCCGGGTACAGCCCCGAAACTGCTGATCTATGATAATAATCAGCGCCCGAGCGGTGTTCCGGATCGCTTTAGCGGTAGCAAAAGTGGTACAAGCGCCAGCCTGGCAATTACCGGCCTGCAGAGCGAAGATGAAGCAGATTATTATTGTCAGGTTCGTGATAATAACGAAAATGAATGGGTTTTTGGTGGCGGTACAAAACTGACCACC**ggtagtggtttggaggtcttatttcagggtcctggcagtgggggc**CGTGGCTCCATCGACACCTGGGTC***TGATAA*gcggccgccctcctcctcctttcttgttcctttcacgtcgccttctcggttgtagctggcagacgacgagtcttacttttacgtgtacttctctatagatgatgtatgatctctctgcatgcgtgttcgtgcatgtgtccgtgtgttgggtacgcgtggtaccctgcaggaaggaagctgagttggctgctgccaccgctgagcaataactagtaattactagcataaccccttggggcctctaaacgggtcttgagggggttttttgctgaaaggaggacagctgatgattgtcatgcttgccatctgttttcttgcaaggtcagag |

**pLTE-αIFNα-2a(**[**4ypg**](about:blank)**)-Fab-RGS**:

| expression cassette = [T7-5'SITS-*start*-3'SITS-tvmv-**VHCH_VLCL_RGS***stop-*3'utr-terminator] |
| --- |
| TAATACGACTCACTATAGGGACATCTTAAGTTTATTTTATTTTATTTTATTTTATTTTATTTTATTTTATTTTATTTTATTTTATTTTATTTAAcc*ATG*ACAGTAATGTATAAAGTCTGTAAAGACATTAAACACGTAAGTGAAaccatgGAAACAGTGCGTTTCCAG**AGCCAAGTTCAACTgGTACAGTCAGGAGCTGAAGTAAAGAAACCGGGTGCGTCCGTTAAGGTCAGCTGTAAAGCCTCGGGCTACACCTTCACCTCCTATAGCATCTCATGGGTTCGCCAGGCCCCtGGTCAAGGTCTGGAGTGGATGGGTTGGATTAGCGTCTACAACGGTAATACCAATTATGCGCAGAAATTCCAGGGTCGTGTTACCATGACCACCGACACCAGCACCTCCACCGCGTATCTGGAGCTGCGTTCCCTGCGCAGCGATGATACCGCGGTGTACTACTGCGCTCGTGATCCGATTGCGGCGGGTTATTGGGGCCAAGGTACGTTGGTTACTGTCTCTTCTGCATCTACCAAGGGTCCaTCCGTCTTTCCGCTGGCGCCAAGCAGCAAGAGCACCTCCGGAGGAACGGCTGCCCTtGGCTGCCTGGTTAAAGACTACTTCCCaGAGCCaGTTACAGTTAGCTGGAACTCCGGCGCACTGACCTCAGGCGTTCATACCTTCCCaGCAGTGCTGCAGAGCAGCGGCCTGTATAGCCTTAGCTCCGTGGTTACTGTACCTTCcTCTTCcTTGGGTACCCAGACGTACATCTGTAATGTTAACCACAAGCCGAGCAACACCAAGGTGGACAAAAAGGTGGAGCCGAAAAGCTGCGGTGGCAGTGGTGGCTCTAGCGGCGGCTCCGGAGGCTCTGGCGGCAGCTCCGGTGGCAGTGGTTCCGGTTCCGGTTCCGGTTCAGGCTCCGGTAGCGGCGGTTCAGGCGGTAGCGGTGGCGAGATCGTGTTGACGCAGAGCCCAGGGACCcttAGCCTCAGCCCaGGCGAGCGCGCGACCCTGAGTTGCCGTGCTAGCCAATCTGTTAGCAGCACGTACCTGGCATGGTATCAGCAGAAACCtGGCCAAGCTCCtCGCCTGCTGATTTATGGCGCGTCGAGTCGTGCGACGGGTATCCCGGATCGTTTTAGCGGCTCGGGTTCTGGCACTGACTTCACCTTGACCATTAGCCGTTTGGAGCCaGAAGATTTCGCCGTTTACTACTGCCAGCAATATGGTAGCAGCCCGAGAACATTCGGTCAAGGTACCAAGGTCGAAATCAAACGTACCGTGGCGGCTCCGAGCGTGTTTATCTTTCCTCCGAGCGACGAACAGCTCAAATCCGGCACTGCCTCCGTGGTGTGCCTGCTGAATAACTTTTATCCGCGTGAAGCAAAAGTGCAGTGGAAAGTGGACAACGCGCTGCAAAGCGGCAACTCGCAAGAAAGCGTGACGGAACAAGATAGCAAGGACAGCACCTACAGCTTGTCTTCCACCCTGACCCTGTCAAAGGCGGATTACGAAAAACACAAAGTTTACGCATGTGAAGTTACCCATCAGGGTCTGTCTTCACCGGTGACGAAGTCCTTTAACCGCGGTGAGTGCACC**ggtagtggtttggaggtcttatttcagggtcctggcagtgggggc**CGTGGCTCCATCGACACCTGGGTC***TGATAA*gcggccgccctcctcctcctttcttgttcctttcacgtcgccttctcggttgtagctggcagacgacgagtcttacttttacgtgtacttctctatagatgatgtatgatctctctgcatgcgtgttcgtgcatgtgtccgtgtgttgggtacgcgtggtaccctgcaggaaggaagctgagttggctgctgccaccgctgagcaataactagtaattactagcataaccccttggggcctctaaacgggtcttgagggggttttttgctgaaaggaggacagctgatgattgtcatgcttgccatctgttttcttgcaaggtcagag |

**pLTE-αIL-6(**[**4cni**](about:blank)**) scFv-RGS**:

| expression cassette = [T7-5'SITS-*start*-3'SITS-tvmv-**VHCH_VLCL_RGS***stop-*3'utr-terminator] |
| --- |
| TAATACGACTCACTATAGGGACATCTTAAGTTTATTTTATTTTATTTTATTTTATTTTATTTTATTTTATTTTATTTTATTTTATTTTATTTAAcc*ATG*ACAGTAATGTATAAAGTCTGTAAAGACATTAAACACGTAAGTGAAaccatgGAAACAGTGCGTTTCCAG**AGCCAAGTTCAACTgGTACAGTCAGGAGCTGAAGTAAAGAAACCGGGTGCGTCCGTTAAGGTCAGCTGTAAAGCCTCGGGCTACACCTTCACCTCCTATAGCATCTCATGGGTTCGCCAGGCCCCtGGTCAAGGTCTGGAGTGGATGGGTTGGATTAGCGTCTACAACGGTAATACCAATTATGCGCAGAAATTCCAGGGTCGTGTTACCATGACCACCGACACCAGCACCTCCACCGCGTATCTGGAGCTGCGTTCCCTGCGCAGCGATGATACCGCGGTGTACTACTGCGCTCGTGATCCGATTGCGGCGGGTTATTGGGGCCAAGGTACGTTGGTTACTGTCTCTTCTGCATCTACCAAGGGTCCaTCCGTCTTTCCGCTGGCGCCAAGCAGCAAGAGCACCTCCGGAGGAACGGCTGCCCTtGGCTGCCTGGTTAAAGACTACTTCCCaGAGCCaGTTACAGTTAGCTGGAACTCCGGCGCACTGACCTCAGGCGTTCATACCTTCCCaGCAGTGCTGCAGAGCAGCGGCCTGTATAGCCTTAGCTCCGTGGTTACTGTACCTTCcTCTTCcTTGGGTACCCAGACGTACATCTGTAATGTTAACCACAAGCCGAGCAACACCAAGGTGGACAAAAAGGTGGAGCCGAAAAGCTGCGGTGGCAGTGGTGGCTCTAGCGGCGGCTCCGGAGGCTCTGGCGGCAGCTCCGGTGGCAGTGGTTCCGGTTCCGGTTCCGGTTCAGGCTCCGGTAGCGGCGGTTCAGGCGGTAGCGGTGGCGAGATCGTGTTGACGCAGAGCCCAGGGACCcttAGCCTCAGCCCaGGCGAGCGCGCGACCCTGAGTTGCCGTGCTAGCCAATCTGTTAGCAGCACGTACCTGGCATGGTATCAGCAGAAACCtGGCCAAGCTCCtCGCCTGCTGATTTATGGCGCGTCGAGTCGTGCGACGGGTATCCCGGATCGTTTTAGCGGCTCGGGTTCTGGCACTGACTTCACCTTGACCATTAGCCGTTTGGAGCCaGAAGATTTCGCCGTTTACTACTGCCAGCAATATGGTAGCAGCCCGAGAACATTCGGTCAAGGTACCAAGGTCGAAATCAAACGTACCGTGGCGGCTCCGAGCGTGTTTATCTTTCCTCCGAGCGACGAACAGCTCAAATCCGGCACTGCCTCCGTGGTGTGCCTGCTGAATAACTTTTATCCGCGTGAAGCAAAAGTGCAGTGGAAAGTGGACAACGCGCTGCAAAGCGGCAACTCGCAAGAAAGCGTGACGGAACAAGATAGCAAGGACAGCACCTACAGCTTGTCTTCCACCCTGACCCTGTCAAAGGCGGATTACGAAAAACACAAAGTTTACGCATGTGAAGTTACCCATCAGGGTCTGTCTTCACCGGTGACGAAGTCCTTTAACCGCGGTGAGTGCACC**ggtagtggtttggaggtcttatttcagggtcctggcagtgggggc**CGTGGCTCCATCGACACCTGGGTC***TGATAA*gcggccgccctcctcctcctttcttgttcctttcacgtcgccttctcggttgtagctggcagacgacgagtcttacttttacgtgtacttctctatagatgatgtatgatctctctgcatgcgtgttcgtgcatgtgtccgtgtgttgggtacgcgtggtaccctgcaggaaggaagctgagttggctgctgccaccgctgagcaataactagtaattactagcataaccccttggggcctctaaacgggtcttgagggggttttttgctgaaaggaggacagctgatgattgtcatgcttgccatctgttttcttgcaaggtcagag |

**pLTE-αIL-6(**[**4cni**](about:blank)**) scFv-RGS**:

| expression cassette = [T7-5'SITS-*start*-3'SITS-tvmv-**VH_VL_RGS***stop-*3'utr-terminator] |
| --- |
| TAATACGACTCACTATAGGGACATCTTAAGTTTATTTTATTTTATTTTATTTTATTTTATTTTATTTTATTTTATTTTATTTTATTTTATTTAAcc*ATG*ACAGTAATGTATAAAGTCTGTAAAGACATTAAACACGTAAGTGAAaccatgGAAACAGTGCGTTTCCAG**GTTCAGCTGGTTGAAAGTGGTGGTGGTCTGGTTCAGCCGGGTGGCAGCCTGCGCCTGAGTTGTGCAGCCAGTGGTTTTAATTTTAATGATTATTTCATGAACTGGGTGCGCCAGGCCCCTGGTAAAGGTCTGGAATGGGTTGCCCAGATGCGTAATAAGAATTATCAGTATGGTACATACTACGCCGAAAGCCTGGAAGGTCGCTTTACCATTAGTCGCGATGATAGTAAAAATAGTCTGTATCTGCAGATGAATAGTCTGAAAACCGAAGATACCGCCGTTTATTATTGTGCCCGCGAAAGCTATTATGGCTTTACCAGCTATTGGGGCCAGGGCACCCTGGTTACAGTTAGCGGTGGTAGCGGCGGCGGCAGCGGTGGTGGTAGTGGTGGTGGCAGTGGTGGTGGAATTCAGATGACCCAGAGCCCGAGTAGTCTGAGCGCAAGCGTGGGTGACAGAGTTACCATTACCTGTCAGGCAAGCCAGGATATTGGTATTAGTCTGAGTTGGTATCAGCAGAAACCGGGTAAAGCCCCGAAACTGCTGATCTATAATGCCAATAATCTGGCAGATGGCGTTCCGAGTCGCTTTAGTGGCAGCGGCAGCGGTACAGATTTTACCCTGACCATTAGTAGCCTGCAGCCGGAAGATTTTGCCACCTATTATTGTCTGCAGCATAATAGCGCACCGTATACCTTTGGCCAGGGTACAAAACTGGAAACC**ggtagtggtttggaggtcttatttcagggtcctggcagtgggggc**CGTGGCTCCATCGACACCTGGGTC***TGATAA*gcggccgccctcctcctcctttcttgttcctttcacgtcgccttctcggttgtagctggcagacgacgagtcttacttttacgtgtacttctctatagatgatgtatgatctctctgcatgcgtgttcgtgcatgtgtccgtgtgttgggtacgcgtggtaccctgcaggaaggaagctgagttggctgctgccaccgctgagcaataactagtaattactagcataaccccttggggcctctaaacgggtcttgagggggttttttgctgaaaggaggacagctgatgattgtcatgcttgccatctgttttcttgcaaggtcagag |

**pLTE-αIL-6(**[**4zs7**](about:blank)**) Fab-RGS**:

| expression cassette = [T7-5'SITS-*start*-3'SITS-tvmv-**VHCH_VLCL_RGS***stop-*3'utr-terminator] |
| --- |
| TAATACGACTCACTATAGGGACATCTTAAGTTTATTTTATTTTATTTTATTTTATTTTATTTTATTTTATTTTATTTTATTTTATTTTATTTAAcc*ATG*ACAGTAATGTATAAAGTCTGTAAAGACATTAAACACGTAAGTGAAaccatgGAAACAGTGCGTTTCCAG**AGCGAGGTTCAGCTCCAGGAGTCAGGCCCAGGCCTCGTAAAGCCTAGTCAAACGTTGTCTCTGACATGCACGGTATCAGGAGGGTCAATCACGACCCGCTACTACGCGTGGTCATGGATTCGGCAACCACCCGGTAAAGGTCTGGAATGGATGGGCGTGATTGATTATGACGGTGACACTTACTATAGTCCCAGTCTTAAATCACGcACATCTATATCTTGGGACACAAGTAAGAATCAGTTTTCCCTGCAATTGTCTTCCGTGACCCCAGAAGATACGGCGGTGTATTATTGTGCCCGtGATCCTGACGTGGTTACTGGGTTTCATTATGATTACTGGGGGCAAGGTACACAGGTCACAGTATCATCTGCCTCAACGAAAGGGCCCAGCGTTTTCCCGCTGGCACCAAGCAGTAAGAGCACGTCTGGAGGGACGGCAGCACTGGGATGCCTCGTTAAGGATTACTTCCCGGAACCTGTTACTGTGTCATGGAATTCAGGCGCGCTTACCTCAGGGGTGCACACATTCCCCGCGGTCCTGCAGTCATCCGGGCTTTACAGTCTGAGTTCAGTTGTCACAGTCCCTTCATCAAGCTTGGGCACACAAACATACATTTGTAACGTGAACCATAAACCCTCTAACACAAAAGTAGACAAAAAAGTTGAGCCGGGCGGATCTGGCGGTAGTTCAGGGGGCAGCGGCGGGTCTGGAGGCAGCAGTGGGGGTTCTGGCAGTGGGTCAGGTAGTGGAAGTGGGTCTGGTTCTGGAGGTAGCGGTGGCTCTGGGGGCCAAGCCGTTCTGACACAACCGCCATTGGTATCAGGCACTCCAGGTCAGACGGTTACCATATCTTGTGCCGGGGCCAATAACGATATCGGAACCTACGCCTATGTAAGCTGGTACCAACAGCTTCCGGGAACGGCGCCAAAACTTCTGATATATAAGGTCACGACTCGtGCCAGTGGTATTCCATCCAGGTTCTCAGGCAGCAAATCAGGGAACACAGCCAGCCTTACTATTTCCGGTTTGCAATCCGAAGACGAGGCAGACTACTACTGTGCCTCCTATCGcAACTTTAATAATGCGGTTTTCGGTAGAGGAACGCACCTGACAGTACTTGGACAACCGAAGGCAGCTCCAAGTGTAACCCTGTTTCCGCCATCTTCAGAAGAGTTGCAGGCCAATAAGGCTACCCTTGTGTGCTTGATATCTGATTTCTACCCAGGAGCTGTCACCGTGGCCTGGAAGGCGGACTCTAGCCCTGTAAAGGCAGGTGTTGAAACGACGACACCAAGCAAACAGTCTAACAATAAATATGCAGCAAGCAGCTACTTGAGTCTCACGCCTGAACAGTGGAAAAGTCATAGGTCTTACTCTTGCCAAGTCACTCATGAAGGCTCTACCGTTGAGAAGACAGTAGCCCCAACAGAATGCAGCACC**ggtagtggtttggaggtcttatttcagggtcctggcagtgggggc**CGTGGCTCCATCGACACCTGGGTC***TGATAA*gcggccgccctcctcctcctttcttgttcctttcacgtcgccttctcggttgtagctggcagacgacgagtcttacttttacgtgtacttctctatagatgatgtatgatctctctgcatgcgtgttcgtgcatgtgtccgtgtgttgggtacgcgtggtaccctgcaggaaggaagctgagttggctgctgccaccgctgagcaataactagtaattactagcataaccccttggggcctctaaacgggtcttgagggggttttttgctgaaaggaggacagctgatgattgtcatgcttgccatctgttttcttgcaaggtcagag |

**pLTE-SARS-COV2-RBD-RGS**:

| expression cassette = [T7-5'SITS-*start*-3'SITS-tvmv-**RBD_RGS***stop-*3'utr-terminator] |
| --- |
| TAATACGACTCACTATAGGGACATCTTAAGTTTATTTTATTTTATTTTATTTTATTTTATTTTATTTTATTTTATTTTATTTTATTTTATTTAAcc*ATG*ACAGTAATGTATAAAGTCTGTAAAGACATTAAACACGTAAGTGAAaccatgGAAACAGTGCGTTTCCAG**CGCGTTCAACCAACCGAGAGCATTGTGCGCTTTCCAAATATTACAAACCTTTGTCCATTTGGAGAGGTTTTCAATGCGACGCGCTTCGCAAGTGTCTATGCCTGGAACCGCAAAAGAATTTCCAACTGTGTTGCTGATTACTCTGTCCTGTATAACTCTGCAAGTTTCAGCACCTTTAAATGTTACGGTGTTTCCCCCACTAAACTTAACGATTTATGTTTTACTAACGTGTACGCTGACTCGTTCGTCATAAGAGGGGATGAGGTAAGACAAATCGCTCCTGGTCAGACTGGTAAAATTGCTGACTACAATTACAAGTTACCCGATGACTTTACCGGCTGTGTCATCGCTTGGAATTCCAACAACTTAGACAGCAAGGTGGGTGGCAACTATAATTATTTGTATCGTTTGTTCAGAAAGTCCAATCTTAAACCATTTGAACGGGACATCTCTACAGAGATATACCAGGCCGGTTCGACCCCTTGTAACGGGGTCGAAGGGTTCAATTGTTACTTTCCATTACAAAGCTACGGCTTTCAACCAACGAATGGAGTGGGGTATCAACCGTACAGAGTAGTAGTCTTAAGTTTCGAGTTACTTCATGCGCCTGCCACCGTATGCGGCCCCAAAAAAAGCACGAATTTAGTCAAGAACAAATGCGTCAATTTTACC**ggtagtggtttggaggtcttatttcagggtcctggcagtgggggc**CGTGGCTCCATCGACACCTGGGTC***TGATAA*gcggccgccctcctcctcctttcttgttcctttcacgtcgccttctcggttgtagctggcagacgacgagtcttacttttacgtgtacttctctatagatgatgtatgatctctctgcatgcgtgttcgtgcatgtgtccgtgtgttgggtacgcgtggtaccctgcaggaaggaagctgagttggctgctgccaccgctgagcaataactagtaattactagcataaccccttggggcctctaaacgggtcttgagggggttttttgctgaaaggaggacagctgatgattgtcatgcttgccatctgttttcttgcaaggtcagag |

**pLTE-GFP-ACE2-RGS**:

| expression cassette = [T7-5'SITS-*start*-3'SITS-tvmv-6x**His**_**GFP_ACE2***stop-*3'utr-terminator] |
| --- |
| TAATACGACTCACTATAGGGACATCTTAAGTTTATTTTATTTTATTTTATTTTATTTTATTTTATTTTATTTTATTTTATTTTATTTTATTTAAcc*ATG*ACAGTAATGTATAAAGTCTGTAAAGACATTAAACACGTAAGTGAAaccatgGAAACAGTGCGTTTCCAGatgggcacctctggt**caccaccatcaccaccatcaccac**ggcaca**AGCAAGGGCGAGGAGCTGTTCACCGGGGTGGTGCCCATCCTGGTCGAGCTGGACGGCGACGTAAACGGCCACAAGTTCAGCGTGTCCGGCGAGGGCGAGGGCGATGCCACCTACGGCAAGCTGACCCTGAAGTTCATCTGCACCACCGGCAAGCTGCCCGTGCCCTGGCCCACCCTCGTGACCACCCTGACCTACGGCGTGCAGTGCTTCAGCCGCTACCCCGACCACATGAAGCAGCACGACTTCTTCAAGTCCGCCATGCCCGAAGGCTACGTCCAGGAGCGCACCATCTTCTTCAAGGACGACGGCAACTACAAGACCCGCGCCGAGGTGAAGTTCGAGGGCGACACCCTGGTGAACCGCATCGAGCTGAAGGGCATCGACTTCAAGGAGGACGGCAACATCCTGGGGCACAAGCTGGAGTACAACTACAACAGCCACAACGTCTATATCATGGCCGACAAGCAGAAGAACGGCATCAAGGTGAACTTCAAGATCCGCCACAACATCGAGGACGGCAGCGTGCAGCTCGCCGACCACTACCAGCAGAACACCCCCATCGGCGACGGCCCCGTGCTGCTGCCCGACAACCACTACCTGAGCACCCAGTCCGCCCTGAGCAAAGACCCCAACGAGAAGCGCGATCACATGGTCCTGCTGGAGTTCGTGACCGCCGCCGGGATCACTCTCGGCATGGACGAGCTATACAAG**ctggaggtgctcttccagggtccg**AGCACAATTGAGGAACAGGCGAAAACATTCCTGGATAAATTTAATCATGAAGCAGAGGACTTGTTCTACCAATCTAGCTTGGCGTCATGGAATTACAATACCAATATCACGGAGGAAAACGTGCAAAATATGAATAACGCGGGAGACAAATGGTCAGCGTTTCTAAAAGAACAGAGTACACTTGCTCAGATGTATCCACTTCAGGAGATTCAAAACTTAACGGTGAAGCTGCAACTGCAAGCTCTTCAACAAAACGGGAGCTCTGTTCTGTCCGAGGATAAGTCTAAGAGGCTAAACACTATTTTGAACACAATGTCCACTATTTATTCCACAGGAAAGGTCTGTAACCCCGACAATCCTCAAGAGTGTCTGCTTCTAGAACCTGGCTTGAACGAAATAATGGCTAATTCACTGGATTATAACGAGCGTCTTTGGGCTTGGGAGTCCTGGAGAAGTGAAGTTGGTAAGCAGCTACGTCCTTTATACGAAGAATACGTGGTGTTAAAAAATGAGATGGCAAGAGCCAATCACTACGAGGACTACGGCGACTATTGGCGTGGAGATTACGAGGTCAACGGTGTAGATGGTTATGACTACTCCAGAGGCCAATTAATAGAAGATGTTGAGCACACGTTCGAAGAAATCAAGCCCTTGTATGAACACCTACACGCCTACGTTAGGGCCAAGTTAATGAATGCGTATCCTAGCTATATAAGCCCGATAGGATGCTTGCCCGCACACTTGCTTGGCGACATGTGGGGCCGTTTCTGGACGAACCTATACTCCTTAACAGTACCGTTCGGTCAGAAACCTAATATTGATGTGACAGACGCAATGGTCGATCAGGCGTGGGACGCCCAAAGGATTTTCAAGGAAGCGGAGAAATTCTTTGTCTCAGTGGGACTACCTAACATGACACAAGGATTCTGGGAGAACAGCATGCTGACTGACCCAGGCAACGTCCAGAAGGCAGTATGTCATCCCACGGCGTGGGACTTGGGTAAGGGGGATTTTAGAATACTAATGTGCACCAAGGTGACAATGGACGATTTTTTGACGGCACACCACGAGATGGGTCATATTCAGTACGACATGGCATACGCGGCACAACCGTTTTTGTTGAGGAATGGGGCTAACGAGGGCTTTCACGAAGCCGTTGGCGAGATAATGTCTTTATCTGCGGCTACGCCTAAGCATTTAAAATCCATAGGACTACTGAGCCCCGATTTTCAGGAGGATAATGAAACGGAGATTAATTTTCTATTGAAACAAGCTCTAACTATTGTTGGAACGTTACCGTTTACATATATGCTAGAGAAGTGGAGATGGATGGTTTTCAAGGGCGAGATACCTAAAGACCAATGGATGAAAAAGTGGTGGGAGATGAAGCGTGAAATCGTAGGAGTTGTTGAGCCAGTCCCTCACGATGAGACTTATTGTGACCCTGCAAGTTTGTTCCACGTATCAAATGATTATTCATTCATTAGGTACTACACGAGGACGCTATACCAGTTCCAATTCCAAGAAGCACTTTGCCAGGCGGCTAAACATGAGGGACCCTTGCACAAGTGCGATATAAGCAACTCAACAGAGGCCGGACAAAAATTATTCAACATGTTGCGTCTAGGTAAATCCGAACCTTGGACCCTTGCGCTTGAAAATGTCGTGGGCGCGAAGAACATGAATGTGCGTCCTCTGCTAAACTATTTCGAGCCCCTTTTTACCTGGCTTAAGGATCAGAATAAGAACAGTTTCGTCGGATGGAGTACCGACTGGTCCCCATACGCCGAT***TAA*gcggccgccctcctcctcctttcttgttcctttcacgtcgccttctcggttgtagctggcagacgacgagtcttacttttacgtgtacttctctatagatgatgtatgatctctctgcatgcgtgttcgtgcatgtgtccgtgtgttgggtacgcgtggtaccctgcaggaaggaagctgagttggctgctgccaccgctgagcaataactagtaattactagcataaccccttggggcctctaaacgggtcttgagggggttttttgctgaaaggaggacagctgatgattgtcatgcttgccatctgttttcttgcaaggtcagag |

**pLTE-ACE2-Cherry-RGS**:

| expression cassette = [T7-5'SITS-*start*-3'SITS-tvmv-**ACE2_Cherry_6xHis_MYC***stop-*3'utr-terminator] |
| --- |
| TAATACGACTCACTATAGGGACATCTTAAGTTTATTTTATTTTATTTTATTTTATTTTATTTTATTTTATTTTATTTTATTTTATTTTATTTAAcc*ATG*ACAGTAATGTATAAAGTCTGTAAAGACATTAAACACGTAAGTGAAaccatgGAAACAGTGCGTTTCCAG**ATGACAGTAATGTATAAAGTCTGTAAAGACATTAAACACGTAAGTGAAACCATGGGCACAAGTTTGTACAAAAAAGCAGGCTTAAGCACAATTGAGGAACAGGCGAAAACATTCCTGGATAAATTTAATCATGAAGCAGAGGACTTGTTCTACCAATCTAGCTTGGCGTCATGGAATTACAATACCAATATCACGGAGGAAAACGTGCAAAATATGAATAACGCGGGAGACAAATGGTCAGCGTTTCTAAAAGAACAGAGTACACTTGCTCAGATGTATCCACTTCAGGAGATTCAAAACTTAACGGTGAAGCTGCAACTGCAAGCTCTTCAACAAAACGGGAGCTCTGTTCTGTCCGAGGATAAGTCTAAGAGGCTAAACACTATTTTGAACACAATGTCCACTATTTATTCCACAGGAAAGGTCTGTAACCCCGACAATCCTCAAGAGTGTCTGCTTCTAGAACCTGGCTTGAACGAAATAATGGCTAATTCACTGGATTATAACGAGCGTCTTTGGGCTTGGGAGTCCTGGAGAAGTGAAGTTGGTAAGCAGCTACGTCCTTTATACGAAGAATACGTGGTGTTAAAAAATGAGATGGCAAGAGCCAATCACTACGAGGACTACGGCGACTATTGGCGTGGAGATTACGAGGTCAACGGTGTAGATGGTTATGACTACTCCAGAGGCCAATTAATAGAAGATGTTGAGCACACGTTCGAAGAAATCAAGCCCTTGTATGAACACCTACACGCCTACGTTAGGGCCAAGTTAATGAATGCGTATCCTAGCTATATAAGCCCGATAGGATGCTTGCCCGCACACTTGCTTGGCGACATGTGGGGCCGTTTCTGGACGAACCTATACTCCTTAACAGTACCGTTCGGTCAGAAACCTAATATTGATGTGACAGACGCAATGGTCGATCAGGCGTGGGACGCCCAAAGGATTTTCAAGGAAGCGGAGAAATTCTTTGTCTCAGTGGGACTACCTAACATGACACAAGGATTCTGGGAGAACAGCATGCTGACTGACCCAGGCAACGTCCAGAAGGCAGTATGTCATCCCACGGCGTGGGACTTGGGTAAGGGGGATTTTAGAATACTAATGTGCACCAAGGTGACAATGGACGATTTTTTGACGGCACACCACGAGATGGGTCATATTCAGTACGACATGGCATACGCGGCACAACCGTTTTTGTTGAGGAATGGGGCTAACGAGGGCTTTCACGAAGCCGTTGGCGAGATAATGTCTTTATCTGCGGCTACGCCTAAGCATTTAAAATCCATAGGACTACTGAGCCCCGATTTTCAGGAGGATAATGAAACGGAGATTAATTTTCTATTGAAACAAGCTCTAACTATTGTTGGAACGTTACCGTTTACATATATGCTAGAGAAGTGGAGATGGATGGTTTTCAAGGGCGAGATACCTAAAGACCAATGGATGAAAAAGTGGTGGGAGATGAAGCGTGAAATCGTAGGAGTTGTTGAGCCAGTCCCTCACGATGAGACTTATTGTGACCCTGCAAGTTTGTTCCACGTATCAAATGATTATTCATTCATTAGGTACTACACGAGGACGCTATACCAGTTCCAATTCCAAGAAGCACTTTGCCAGGCGGCTAAACATGAGGGACCCTTGCACAAGTGCGATATAAGCAACTCAACAGAGGCCGGACAAAAATTATTCAACATGTTGCGTCTAGGTAAATCCGAACCTTGGACCCTTGCGCTTGAAAATGTCGTGGGCGCGAAGAACATGAATGTGCGTCCTCTGCTAAACTATTTCGAGCCCCTTTTTACCTGGCTTAAGGATCAGAATAAGAACAGTTTCGTCGGATGGAGTACCGACTGGTCCCCATACGCCGAT**aacccagctttcttgtacaaagtggtgctggaggtgctcttccagggcccg**AAGGGCGAGGAGGATAACATGGCCATCATCAAGGAGTTCATGCGCTTCAAGGTGCACATGGAGGGCTCCGTGAACGGCCACGAGTTCGAGATCGAGGGCGAGGGCGAGGGCCGCCCCTACGAGGGCACCCAGACCGCCAAGCTGAAGGTGACCAAGGGTGGCCCCCTGCCCTTCGCCTGGGACATCCTGTCCCCTCAGTTCATGTACGGCTCCAAGGCCTACGTGAAGCACCCCGCCGACATCCCCGACTACTTGAAGCTGTCCTTCCCCGAGGGCTTCAAGTGGGAGCGCGTGATGAACTTCGAGGACGGCGGCGTGGTGACCGTGACCCAGGACTCCTCCCTGCAGGACGGCGAGTTCATCTACAAGGTGAAGCTGCGCGGCACCAACTTCCCCTCCGACGGCCCCGTAATGCAGAAGAAGACTATGGGCTGGGAGGCCTCCTCCGAGCGGATGTACCCCGAGGACGGCGCCCTGAAGGGCGAGATCAAGCAGAGGCTGAAGCTGAAGGACGGCGGCCACTACGACGCTGAGGTCAAGACCACCTACAAGGCCAAGAAGCCCGTGCAGCTGCCCGGCGCCTACAACGTCAACATCAAGTTGGACATCACCTCCCACAACGAGGACTACACCATCGTGGAACAGTACGAACGCGCCGAGGGCCGCCACTCCACCGGCGGCATGGACGAGCTGTACAAGACC**acc**caccaccatcaccaccatcaccacGAGCAGAAGCTGATCTCGGAGGAGGATCTGTAA***TAA*gcggccgccctcctcctcctttcttgttcctttcacgtcgccttctcggttgtagctggcagacgacgagtcttacttttacgtgtacttctctatagatgatgtatgatctctctgcatgcgtgttcgtgcatgtgtccgtgtgttgggtacgcgtggtaccctgcaggaaggaagctgagttggctgctgccaccgctgagcaataactagtaattactagcataaccccttggggcctctaaacgggtcttgagggggttttttgctgaaaggaggacagctgatgattgtcatgcttgccatctgttttcttgcaaggtcagag |

**Constructs based on pOPINE:**

**pOPINE-backbone:**

| cgaaat**[expression_cassette]**ctgaaagcatgcggaggaaattctccttgaagtttccctggtgttcaaagtaaaggagtttgcaccagacgcacctctgttcactggtccggcgtattaaaacacgatacattgttattagtacatttattaagcgctagattctgtgcgttgttgatttacagacaattgttgtacgtattttaataattcattaaatttataatctttagggtggtatgttagagcgaaaatcaaatgattttcagcgtctttatatctgaatttaaatattaaatcctcaatagatttgtaaaataggtttcgattagtttcaaacaagggttgtttttccgaaccgatggctggactatctaatggattttcgctcaacgccacaaaacttgccaaatcttgtagcagcaatctagctttgtcgatattcgtttgtgttttgttttgtaataaaggttcgacgtcgttcaaaatattatgcgcttttgtatttctttcatcactgtcgttagtgtacaattgactcgacgtaaacacgttaaatagagcttggacatatttaacatcgggcgtgttagctttattaggccgattatcgtcgtcgtcccaaccctcgtcgttagaagttgcttccgaagacgattttgccatagccacacgacgcctattaattgtgtcggctaacacgtccgcgatcaaatttgtagttgagctttttggaattgcgatcgcataacttcgtatagcatacattatacgaagttataagctcggaacgctgcgctcggtcgttcggctgcggcgagcggtatcagctcactcaaaggcggtaatacggttatccacagaatcaggggataacgcaggaaagaacatgtgagcaaaaggccagcaaaaggccaggaaccgtaaaaaggccgcgttgctggcgtttttccataggctccgccccctgacgagcatcacaaaaatcgacgctcaagtcagaggtggcgaaacccgacaggactataaagataccaggcgtttccccctggaagctccctcgtgcgctctcctgttccgaccctgccgcttaccggatacctgtccgcctttctcccttcgggaagcgtggcgctttctcaatgctcacgctgtaggtatctcagttcggtgtaggtcgttcgctccaagctgggctgtgtgcacgaaccccccgttcagcccgaccgctgcgccttatccggtaactatcgtcttgagtccaacccggtaagacacgacttatcgccactggcagcagccactggtaacaggattagcagagcgaggtatgtaggcggtgctacagagttcttgaagtggtggcctaactacggctacactagaagaacagtatttggtatctgcgctctgctgaagccagttaccttcggaaaaagagttggtagctcttgatccggcaaacaaaccaccgctggtagcggtggtttttttgtttgcaagcagcagattacgcgcagaaaaaaaggatctcaagaagatcctttgttaccaatgcttaatcagtgaggcacctatctcagcgatctgtctatttcgttcatccatagttgcctgactccccgtcgtgtagataactacgatacgggagggcttaccatctggccccagtgctgcaatgataccgcgagacccacgctcaccggctccagatttatcagcaataaaccagccagccggaagggccgagcgcagaagtggtcctgcaactttatccgcctccatccagtctattaattgttgccgggaagctagagtaagtagttcgccagttaatagtttgcgcaacgttgttgccattgctacaggcatcgtggtgtcacgctcgtcgtttggtatggcttcattcagctccggttcccaacgatcaaggcgagttacatgatcccccatgttgtgcaaaaaagcggttagctccttcggtcctccgatcgttgtcagaagtaagttggccgcagtgttatcactcatggttatggcagcactgcataattctcttactgtcatgccatccgtaagatgcttttctgtgactggtgagtactcaaccaagtcattctgagaatagtgtatgcggcgaccgagttgctcttgcccggcgtcaatacgggataataccgcgccacatagcagaactttaaaagtgctcatcattggaaaacgttcttcggggcgaaaactctcaaggatcttaccgctgttgagatccagttcgatgtaacccactcgtgcacccaactgatcttcagcatcttttactttcaccagcgtttctgggtgagcaaaaacaggaaggcaaaatgccgcaaaaaagggaataagggcgacacggaaatgttgaatactcatactcttcctttttcaatattattgaagcatttatcagggttattgtctcatgtccgcgcgttctctcctgcatcttttaatcaaatcccaagatgtgtataaacgcgccggtatgtacaggaagaggtttatactaaactgttacattgcaaacgtggtttcgtgtgccaagtgtgaaaaccgatgtttaatcaaggctctgacgcatttctacaaccacgactccaagtgtgtgggtgaagtcatgcatcttttaatcaaatcccaagatgtgtataaaccaccaaactgccaaaaaatgaaaactgtcgacaagctctgtccgtttgctggcaactgcaagggtctcaatcctatttgtaattattgaataataaaacaattataaatgtcaaatttgttttttattaacgatacaaaccaaacgcaacaagaacatttgtagtattatctataattgaaaacgcgtagttataatcgctgaggtaatatttaaaatcattttcaaatgattcacagttaatttgcgacaatataattttattttcacataaactagacgccttgtcgtcttcttcttcgtattccttctctttttcatttttctcttcataaaaattaacatagttattatcgtatccatatatgtatctatcgtatagagtaaattttttgttgtcataaatatatatgtcttttttaatggggtgtatagtaccgctgcgcatagtttttctgtaatttacaacagtgctattttctggtagttcttcggagtgtgttgctttaattattaaatttatataatcaatgaatttgggatcgtcggttttgtacaatatgttgccggcatagtacgcagcttcttctagttcaattacaccattttttagcagcaccggattaacataactttccaaaatgttgtacgaaccgttaaacaaaaacagttcacctcccttttctatactattgtctgcgagcagttgtttgttgttaaaaataacagccattgtaatgagacgcacaaactaatatcacaaactggaaatgtctatcaatatatagttgctgatggccggccgtaatgagacgcacaaactaatatcacaaactggaaatgtctatcaatatatagttgctctagttattaatagtaatcaattacggggtcattagttcatagcccatatatggagttccgcgttacataacttacggtaaatggcccgcctggctgaccgcccaacgacccccgcccattgacgtcaataatgacgtatgttcccatagtaacgccaatagggactttccattgacgtcaatgggtggagtatttacggtaaactgcccacttggcagtacatcaagtgtatcatatgccaagtacgccccctattgacgtcaatgacggtaaatggcccgcctggcattatgcccagtacatgaccttatgggactttcctacttggcagtacatctacgtattagtcatcgctattaccatgcatggtcgaggtgagccccacgttctgcttcactctccccatctcccccccctccccacccccaattttgtatttatttattttttaattattttgtgcagcgatgggggcggggggggggggggggcgcgcgccaggcggggcggggcggggcgaggggcggggcggggcgaggcggagaggtgcggcggcagccaatcagagcggcgcgctccgaaagtttccttttatggcgaggcggcggcggcggcggccctataaaaagcgaagcgcgcggcgggcgggagtcgctgcgcgctgccttcgccccgtgccccgctccgccgccgcctcgcgccgcccgccccggctctgactgaccgcgttactcccacaggtgagcgggcgggacggcccttctccttcgggctgtaattagcgcttggtttaatgacggcttgtttcttttctgtggctgcgtgaaagccttgaggggctccgggagggccctttgtgcggggggagcggctcggggctgtccgcggggggacggctgccttcgggggggacggggcagggcggggttcggcttctggcgtgtgaccggcggctctagagcctctgctaaccatgttcatgccttcttctttttcctacagctcctgggcaacgtgctggttattgtgctgtctcatcattttggcaaagaattggatcggac |
| --- |

**pOPINE-(3'SITS)-RGS**:

| expression cassette = [T7-5'utr(SD)-*start*-3'SITS-tvmv-**Dc1a**_RGS*stop-*3'utr-terminator] |
| --- |
| TAATACGACTCACTATAGGGgaattgtgagcggataacaattccccggagttaatccgggacctttaattcaacccaacacaatatattatagttaaataagaattattatcaaatcatttgtatattaattaaaatactatactgtaaattacattttatttacaatca**AAGGAG**atatacc*ATG*ACAGTAATGTATAAAGTCTGTAAAGACATTAAACACttagGTGAAACCATGGAAACAGTGCGTTTCCAGggtaccggtagtggtggcCGTGGCTCCATCGACACCTGGGTC*TGATAA*gcggccgccagctttctagaccagtttaaacatcaccatcaccatcactaagtgattaacctcaggtgcaggctgcctatcagaaggtggtggctggtgtggccaatgccctggctcacaaataccactgagatcgatctttttccctctgccaaaaattatggggacatcatgaagccccttgagcatctgacttctggctaataaaggaaatttattttcattgcaatagtgtgttggaattttttgtgtctctcactcggaaggacatatgggagggcaaatcatttaaaacatcagaatgagtatttggtttagagtttggcaacatatgcccatatgtaactagcataaccccttggggcctctaaacgggtcttgaggggttttttgctagcataaccccttggggcctctaaacgggtcttgaggggttttttg |

**pOPINE-(3'SITS)-SFTI-RGS**:

| expression cassette = [T7-5'utr(SD)-*start*-3'SITS-tvmv-**SFTI**_RGS*stop-*3'utr-terminator] |
| --- |
| TAATACGACTCACTATAGGGgaattgtgagcggataacaattccccggagttaatccgggacctttaattcaacccaacacaatatattatagttaaataagaattattatcaaatcatttgtatattaattaaaatactatactgtaaattacattttatttacaatca**AAGGAG**atatacc*ATG*ACAGTAATGTATAAAGTCTGTAAAGACATTAAACACttagGTGAAACCATGGAAACAGTGCGTTTCCAG**TCCATTCCTCCGATTTGTTTTCCTGATGGCCGTTGTACCAAA**ggtaccggtagtggtggcCGTGGCTCCATCGACACCTGGGTC*TGATAA*gcggccgccagctttctagaccagtttaaacatcaccatcaccatcactaagtgattaacctcaggtgcaggctgcctatcagaaggtggtggctggtgtggccaatgccctggctcacaaataccactgagatcgatctttttccctctgccaaaaattatggggacatcatgaagccccttgagcatctgacttctggctaataaaggaaatttattttcattgcaatagtgtgttggaattttttgtgtctctcactcggaaggacatatgggagggcaaatcatttaaaacatcagaatgagtatttggtttagagtttggcaacatatgcccatatgtaactagcataaccccttggggcctctaaacgggtcttgaggggttttttgctagcataaccccttggggcctctaaacgggtcttgaggggttttttg |

**pOPINE-(3'SITS)-Dc1a-RGS**:

| expression cassette = [T7-5'utr(SD)-*start*-3'SITS-tvmv-**Dc1a**_RGS*stop-*3'utr-terminator] |
| --- |
| TAATACGACTCACTATAGGGgaattgtgagcggataacaattccccggagttaatccgggacctttaattcaacccaacacaatatattatagttaaataagaattattatcaaatcatttgtatattaattaaaatactatactgtaaattacattttatttacaatca**AAGGAG**atatacc*ATG*ACAGTAATGTATAAAGTCTGTAAAGACATTAAACACttagGTGAAACCATGGAAACAGTGCGTTTCCAG**TCGGCTAAAGATGGCGATGTGGAAGGACCCGCTGGTTGCAAGAAATATGATGTCGAGTGTGACTCAGGCGAATGTTGCCAGAAACAATACTTATGGTATAAGTGGCGTCCTTTGGATTGCCGCTGTTTGAAGTCAGGCTTCTTCTCGTCGAAATGTGTTTGCCGTGACGTA**ggtaccggtagtggtggcCGTGGCTCCATCGACACCTGGGTC*TGATAA*gcggccgccagctttctagaccagtttaaacatcaccatcaccatcactaagtgattaacctcaggtgcaggctgcctatcagaaggtggtggctggtgtggccaatgccctggctcacaaataccactgagatcgatctttttccctctgccaaaaattatggggacatcatgaagccccttgagcatctgacttctggctaataaaggaaatttattttcattgcaatagtgtgttggaattttttgtgtctctcactcggaaggacatatgggagggcaaatcatttaaaacatcagaatgagtatttggtttagagtttggcaacatatgcccatatgtaactagcataaccccttggggcctctaaacgggtcttgaggggttttttgctagcataaccccttggggcctctaaacgggtcttgaggggttttttg |

**pOPINE-atg-RGS-atg-GFP**:

| expression cassette = [T7promoter-5'utr-*start*-rgsORF-stop/start-gfpORF-*stop-*3'utr-terminator] |
| --- |
| TAATACGACTCACTATAGGGgaattgtgagcggataacaattccccggagttaatccgggacctttaattcaacccaacacaatatattatagttaaataagaattattatcaaatcatttgtatattaattaaaatactatactgtaaattacattttatttacaatcaAAGGAGatatacc*ATG*cggggctccatcgacacctgggtc*TAATGATG*gcacaccatcaccaccatcacagcagcggtggatccgtgagcaagggcgaggagctgttcaccggggtggtgcccatcctggtcgagctggacggcgacgtaaacggccacaagttcagcgtgtccggcgagggcgagggcgatgccacctacggcaagctgaccctgaagttcatctgcaccaccggcaagctgcccgtgccctggcccaccctcgtgaccaccctgacctacggcgtgcagtgcttcagccgctaccccgaccacatgaagcagcacgacttcttcaagtccgccatgcccgaaggctacgtccaggagcgcaccatcttcttcaaggacgacggcaactacaagacccgcgccgaggtgaagttcgagggcgacaccctggtgaaccgcatcgagctgaagggcatcgacttcaaggaggacggcaacatcctggggcacaagctggagtacaactacaacagccacaacgtctatatcatggccgacaagcagaagaacggcatcaaggtgaacttcaagatccgccacaacatcgaggacggcagcgtgcagctcgccgaccactaccagcagaacacccccatcggcgacggccccgtgctgctgcccgacaaccactacctgagcacccagtccgccctgagcaaagaccccaacgagaagcgcgatcacatggtcctgctggagttcgtgaccgccgccgggatcactctcggcatggacgagctgtacaagtga*TGATAA*gcggccgccagctttctagaccagtttaaacatcaccatcaccatcactaagtgattaacctcaggtgcaggctgcctatcagaaggtggtggctggtgtggccaatgccctggctcacaaataccactgagatcgatctttttccctctgccaaaaattatggggacatcatgaagccccttgagcatctgacttctggctaataaaggaaatttattttcattgcaatagtgtgttggaattttttgtgtctctcactcggaaggacatatgggagggcaaatcatttaaaacatcagaatgagtatttggtttagagtttggcaacatatgcccatatgtaactagcataaccccttggggcctctaaacgggtcttgaggggttttttgctagcataaccccttggggcctctaaacgggtcttgaggggttttttg |

**pOPINE-ttt-RGS-atg-GFP**:

| expression cassette = [T7promoter-5'utr-rgsORF-stop/start-gfpORF-*stop-*3'utr-terminator] |
| --- |
| TAATACGACTCACTATAGGGgaattgtgagcggataacaattccccggagttaatccgggacctttaattcaacccaacacaatatattatagttaaataagaattattatcaaatcatttgtatattaattaaaatactatactgtaaattacattttatttacaatcaAAGGAGatatacc*TTT*cggggctccatcgacacctgggtc*TAATGATG*gcacaccatcaccaccatcacagcagcggtggatccgtgagcaagggcgaggagctgttcaccggggtggtgcccatcctggtcgagctggacggcgacgtaaacggccacaagttcagcgtgtccggcgagggcgagggcgatgccacctacggcaagctgaccctgaagttcatctgcaccaccggcaagctgcccgtgccctggcccaccctcgtgaccaccctgacctacggcgtgcagtgcttcagccgctaccccgaccacatgaagcagcacgacttcttcaagtccgccatgcccgaaggctacgtccaggagcgcaccatcttcttcaaggacgacggcaactacaagacccgcgccgaggtgaagttcgagggcgacaccctggtgaaccgcatcgagctgaagggcatcgacttcaaggaggacggcaacatcctggggcacaagctggagtacaactacaacagccacaacgtctatatcatggccgacaagcagaagaacggcatcaaggtgaacttcaagatccgccacaacatcgaggacggcagcgtgcagctcgccgaccactaccagcagaacacccccatcggcgacggccccgtgctgctgcccgacaaccactacctgagcacccagtccgccctgagcaaagaccccaacgagaagcgcgatcacatggtcctgctggagttcgtgaccgccgccgggatcactctcggcatggacgagctgtacaagtga*TGATAA*gcggccgccagctttctagaccagtttaaacatcaccatcaccatcactaagtgattaacctcaggtgcaggctgcctatcagaaggtggtggctggtgtggccaatgccctggctcacaaataccactgagatcgatctttttccctctgccaaaaattatggggacatcatgaagccccttgagcatctgacttctggctaataaaggaaatttattttcattgcaatagtgtgttggaattttttgtgtctctcactcggaaggacatatgggagggcaaatcatttaaaacatcagaatgagtatttggtttagagtttggcaacatatgcccatatgtaactagcataaccccttggggcctctaaacgggtcttgaggggttttttgctagcataaccccttggggcctctaaacgggtcttgaggggttttttg |

**pOPINE-6hisGFP**:

| expression cassette = [T7-5'utr-*start*-6x**His**_**GFP**-*stop-*3'utr-terminator] |
| --- |
| TAATACGACTCACTATAGGGgaattgtgagcggataacaattccccggagttaatccgggacctttaattcaacccaacacaatatattatagttaaataagaattattatcaaatcatttgtatattaattaaaatactatactgtaaattacattttatttacaatcaAAGGAGatatacc*ATG*gca**caccatcaccaccatcac**agcagcggtggatcc**GTGAGCAAGGGCGAGGAGCTGTTCACCGGGGTGGTGCCCATCCTGGTCGAGCTGGACGGCGACGTAAACGGCCACAAGTTCAGCGTGTCCGGCGAGGGCGAGGGCGATGCCACCTACGGCAAGCTGACCCTGAAGTTCATCTGCACCACCGGCAAGCTGCCCGTGCCCTGGCCCACCCTCGTGACCACCcTgACCTACGGCGTGCAGTGCTTCAGCCGCTACCCCGACCACATGAAGCAGCACGACTTCTTCAAGTCCGCCATGCCCGAAGGCTACGTCCAGGAGCGCACCATCTTCTTCAAGGACGACGGCAACTACAAGACCCGCGCCGAGGTGAAGTTCGAGGGCGACACCCTGGTGAACCGCATCGAGCTGAAGGGCATCGACTTCAAGGAGGACGGCAACATCCTGGGGCACAAGCTGGAGTACAACTACAACAGCCACAACGTCTATATCATGGCCGACAAGCAGAAGAACGGCATCAAGGTGAACTTCAAGATCCGCCACAACATCGAGGACGGCAGCGTGCAGCTCGCCGACCACTACCAGCAGAACACCCCCATCGGCGACGGCCCCGTGCTGCTGCCCGACAACCACTACCTGAGCACCCAGTCCGCCCTGAGCAAAGACCCCAACGAGAAGCGCGATCACATGGTCCTGCTGGAGTTCGTGACCGCCGCCGGGATCACTCTCGGCATGGACGAGCTGTACAAG***TGATAA*gcggccgccagctttctagaccagtttaaacatcaccatcaccatcactaagtgattaacctcaggtgcaggctgcctatcagaaggtggtggctggtgtggccaatgccctggctcacaaataccactgagatcgatctttttccctctgccaaaaattatggggacatcatgaagccccttgagcatctgacttctggctaataaaggaaatttattttcattgcaatagtgtgttggaattttttgtgtctctcactcggaaggacatatgggagggcaaatcatttaaaacatcagaatgagtatttggtttagagtttggcaacatatgcccatatgtaactagcataaccccttggggcctctaaacgggtcttgaggggttttttgctagcataaccccttggggcctctaaacgggtcttgaggggttttttg |

**Supplementary Table S4.** Antisense oligonucleotide sequences used in this study

| Target tRNA: | Name: | Sequence (5’-3’): |
| --- | --- | --- |
| tRNASerGCU | M5-1 | mCmUmUmUmUmGmAmCmCmGmCmAmUmAmCmUmCmCmCmUmUmAmGmC |
| tRNAArgCCU | R7(17-39) | mCmUmUmAmGmGmAmGmGmGmGmCmUmCmGmUmUmAmUmAmUmCmC |

**Supplementary Table S5.** Synthetic tRNA sequences

| Name: | Sequence (5’-3’): |
| --- | --- |
| tRNAAzF4CCU | CCGGCGGUAGUUCAGCAGGGCAGAACGGCGGACUccuAAUCCGCAUGGCAGGGGUUC  AAAUCCCCUCCGCCGGACCA |
| tRNAPylO2ACU | GGAAACGUGAUCAUGUAGAUCGAAUGGACUacuAAUCCGUUCAGUGGGGUUAGAUUC CCCACGUUUCCGCCA |

**Supplementary Table S6.** Accessory protein sequences used in this study

| Name: | Sequence: |
| --- | --- |
| AzFRS.2.t1 | MHHHHHHGSGDEFEMIKRNTSEIISEEELREVLKKDEKSALIGFEPSGKIHLGHYLQIK  KMIDLQNAGFDIIILLADLHAYLNQKGELDEIRKIGDYNKKVFEAMGLKAKYVYGSTYM  LDKDYTLNVYRLALKTTLKRARRSMELIAREDENPKVAEVIYPIMQVNGCHYRGVDVAV  GGMEQRKIHMLARELLPKKVVCIHNPVLTGLDGEGKMSSSKGNFIAVDDSPEEIRAKIK  KAYCPAGVVEGNPIMEIAKYFLEYPLTIKGPEKFGGDLTVNSYEELESLFKNKELHPMR  LKNAVAEELIKILEPIRKRL |
| ChPylRSAF | MAHHHHHHSSGLEVLFQGMDKKPLDVLISATGLWMSRTGTLHKIKHYEISRSKIYIEMAC GDHLVVNNSRSCRPARAFRYHKYRKTCKRCRVSDEDINNFLTRSTEGKTSVKVKVVSEPK VKKAMPKSVSRAPKPLENPVSAKASTDTSRSVPSPAKSTPNSPVPTSASAPALTKSQTDR LEVLLNPKDEISLNSGKPFRELESELLSRRKKDLQQIYAEERENYLGKLEREITRFFVDR GFLEIKSPILIPLEYIERMGIDNDTELSKQIFRVDKNFCLRPMLAPNLANYLRKLDRALP DPIKIFEIGPCYRKESDGKEHLEEFTMLNFCQMGSGCTRENLESIITDFLNHLGIDFKIV GDSCMVFGDTLDVMHGDLELSSAVVGPIPLDREWGIDKPWIGAGFGLERLLKVKHDFKNI KRAARSESYYNGISTNL |
| Affinity clamp (C-cys) | MAHHHHHHSSGTSPELGFSISGGVGGRGNPFRPDDDGIFVTRVQPEGPASKLLQPGDKII  QANGYSFINIEHGQAVSLLKTFQNTVELIIVREVGNGAKQEIRVRVEKDGGSGGVSSVPT  NLEVVAATPTSLLISWDAYRELPVSYYRITYGETGGNSPVQEFTVPGSKSTATISGLKPG  VDYTITVYAHYNYHYYSSPISINYRTSGC |
| strep-GFP-rgs | MTVMYKVCKDIKHLGETMETVRFQASWSHPQFEKGGGSGGGSGGSAWSHPQFEKSSKGEE  LFTGVVPILVELDGDVNGHKFSVSGEGEGDATYGKLTLKFICTTGKLPVPWPTLVTTLTY  GVQCFSRYPDHMKQHDFFKSAMPEGYVQERTIFFKDDGNYKTRAEVKFEGDTLVNRIELK  GIDFKEDGNILGHKLEYNYNSHNVYIMADKQKNGIKVNFKIRHNIEDGSVQLADHYQQNT  PIGDGPVLLPDNHYLSTQSALSKDPNEKRDHMVLLEFVTAAGITLGMDELYKGSGLEVLF  QGPGSGGRGSIDTWV |

**Supplementary Table S7.** Oligonucleotide sequences

Oligonucleotides for site-directed mutagenesis:

| Name: | Sequence (5’-3’): |
| --- | --- |
| SFTI-1AGG-F | TCCTCCGATTaggTTTCCTGATG |
| SFTI-1AGG-R | ATGGACTGGAAACGCACT |
| SFTI-2AGT-F | TGATGGCCGTagtACCAAAGGCG |
| SFTI-2AGT-R | GGAAAACAAATCGGAGGAATGGAC |
| αSL-oligo | CAATAAAGTACAGAAACTGATACTTATATAGCGTT |

Oligonucleotides for generation of tDNA templates:

| Name: | Sequence (5’-3’): |
| --- | --- |
| tRNA^AzF4^CCU-F | GTA ATA CGA CTC ACT ATA CCGGCGGTAGTTCAGCAGGGCAGAACGGCGG |
| tRNA^AzF4^CCU-R1 | GAACCCCTGCCATGCGGATTAGgAGTCCGCCGTTCTGCCCTGC |
| tRNA^AzF4^CCU-R2 | TGGTCCGGCGGAGGGGATTTGAACCCCTGCCATGCGGA |
| tRNA^AzF4^CCU-R3 | TGGTCCGGCGGAGGGGATTT |
|  |  |
| tRNA^PylO2^ACU-F | GTA ATA CGA CTC ACT ATA GGAAACGTGATCATGTAGATCGAA |
| tRNA^PylO2^ACU-R1 | CTAACCCCACTGAACGGATTAGTAGTCCATTCGATCTACA |
| tRNA^PylO2^ACU-R2 | TGGCGGAAACGTGGGGAATCTAACCCCACTGAACGG |
| tRNA^PylO2^ACU-R3 | TGGCGGAAACGTGGGGAAT |

**Supplementary Table S8**. List of abbreviations

| Abbreviation | Definition |
| --- | --- |
| AC-resin | Affinity clamp-coated resin |
| AEP | Asparagine endopeptidase |
| AzF | *p*-azido-L-phenylalanine |
| CFS | Cell free system |
| DHFR | Dihydrofolate reductase |
| Ec CFS | *E. coli* S30-based cell-free system |
| FT | Flow-through |
| GdnHCl | Guanidine hydrochloride |
| GSH | Reduced glutathione |
| HT-1 | Holocyclotoxin-1 |
| LC-MS | Liquid Chromatography-Mass Spectrometry |
| LTE | *Leishmania tarentolae* extract-based cell-free system |
| MALDI | Matrix-assisted laser desorption/ionization |
| McoTI | trypsin inhibitor from *Momordica cochinchinensis* |
| NB | Neutral buffer |
| ncAAs | non-canonical amino acids |
| ON | overnight incubation |
| ORF | Open reading frame |
| POI | Peptide of Interest |
| Prk | n-propargyl-L-lysine |
| prm | circularly permuted peptide backbone |
| PURE | Protein synthesis system Using Recombinant Elements |
| RBD | Receptor-binding domain |
| RES | Resin-bound protein fraction |
| RGS | RGSIDTWV peptide sequence |
| RT | Room temperature |
| SFTI | Sunflower trypsin inhibitor |
| SITS | Species Independent Translation initiation Sequence |
| TCEP | Tris(2-carboxyethyl)phosphine |
| TEV | tobacco etch virus |
| TVMV | tobacco vein mottling virus protease |

**Supplementary note 1** Comparative analysis of peptide yields in Ec CFS and fully reconstituted (PURE) translation system

Major factors limiting the productivity of cell-free translation system may include inefficient ribosomal recycling, degradation of some key component of translational machinery such as ribosomes or translation factors, fast consumption of resources, accumulation of catabolites as well as degradation of peptide product or short peptide-coding RNA template^6, 7^. Therefore, taking advantage of co-translational peptide protection from proteolysis in resin-assisted translation reactions we compared peptide yields in S30-based Ec CFS and PURE translation system which is essentially devoid of any degradation activity. To this end, the ORFs coding for the smallest (SFTI) and the largest (Dc1a) peptide-RGS fusions used in the current study were cloned under the control of either Shine-Dalgarno ribosomal binding sequence (SD-RBS) or species independent translation sequence (SITS) to account for possible difference in initiation preferences between the standard (S30-based) and the minimal (PURE) translation systems. The translation reactions were carried out at 32°C for 1 h and the expression yields of RGS-tagged peptides were monitored by AC-assay as described previously^8^(Fig. 1A). As shown on Fig. 2A, both systems produced similar amounts of RGS-fusion peptides regardless of the type of fusion and initiation motif used. In order to account for possible difference in ribosomal enrichment in two systems RT-qPCR was conducted to quantify 16S rRNA in Ec CFS and PURE-based reactions. Fig. 2B demonstrates 1.56 difference in threshold cycles for 16S rRNA fragment amplification between S30 and PURE systems indicating ~3-fold higher concentration of ribosomal particles in the latter. Similar amounts of RGS-fusion peptides produced in two systems suggest the stability of small peptide-coding RNA template is not a factor limiting peptide productivity in Ec CFS.

**Supplementary note 2** Polymeric open reading frame (ORF) arrangement does not improve the relative molar yield of monomeric SFTI

We next set to test whether polymeric arrangement of peptide-coding units in an ORF can improve the molar yield of peptide. To this end, in addition to monomeric SFTI (SFTIx1) we designed the constructs harboring three or five head-to-tail fused SFTI-coding fragments termed SFTIx3 and SFTIx5, respectively (Fig. 3A). An RGS-coding sequence was appended to the C-terminus of each assembly and R2Q mutation was introduced into each SFTI-coding unit to prevent undesired alternative trypsin cleavage of monomeric and, in particular, polymeric peptide precursors. We hypothesized that if cell-free system productivity is mostly limited by inefficient ribosome recycling the molar ratio of monomeric SFTI derived from mono-, tri- and pentameric ORF arrangements would be close to 1 : 3 : 5, respectively. Alternatively, translation cessation due to expiration of some key translation component(s) would result in a less pronounced difference in the yield of monomeric SFTI between the above constructs due to inverse proportionality between the length and molar yield of the translated precursors in that case. Therefore, at the next step peptide precursors expressed in resin-assisted Ec CFS were purified, eluted and incubated with a catalytic amount of immobilized trypsin. Following quantification of tryptic monomeric SFTI products by LC-MS using the synthetic peptide as a standard (Fig. 3B) we found ~ 1 : 2.4 : 3.5 ratio between the amounts of monomeric SFTI derived from mono-, tri- and pentameric peptide-coding ORFs, respectively. This ratio turned out to be slightly more pronounced than the expected 1 : 2.0 : 2.5 if degradation of key translation component(s) mostly limits the system’s performance (Table 1). On another hand, the calculated molar yields of tri- and pentameric peptide precursors displayed linear size-dependence relative to eGFP while monomeric precursors displayed lower relative yield than expected (Table 1) thereby explaining the difference in above ratio. This discrepancy can be explained by the partial trypsin inhibition by SFTI after rebinding of linear cleavage product where cleavage of polymeric SFTI precursors would proceed to greater degree due to higher local concentration of primary cleavage sites in vicinity to trypsin compared to cleavage of monomeric precursor (Supplementary note 4). Overall, these results together with the data on relative yields obtained for the broad panel of diverse peptides (Table 1) support the inverse linear relationship between molar yield and the length of translated sequence suggesting the expiration of key translation component(s) rather than inefficient ribosome recycling to be the major limitation for the translation efficiency in resin-assisted Ec CFS.

**Supplementary note 3** Overlapping ORF arrangement does not improve the efficiency of *in vitro* peptide synthesis

Degradation of ribosomes or ribosome-associated components was previously identified as a cause of translational cessation in PURE system^6, 7^. Since both, inability to disassemble termination complex or re-assemble the initiation complex, can contribute to the loss of active ribosomal fraction we next attempted to bypass these potential bottleneck stages and extend the productive phase of translation by harnessing the translation reinitiation at the downstream ORF as a mean of increasing the peptide yield. To this end we used overlapping ORF arrangement in the polycistron which is common to more than 25% of bacterial gene pairs^9^. In order to test the overlapping arrangement first, the RGS-coding ORF under control of SITS or SD-RBS was cloned to generate -1 overlap with the downstream eGFP-coding ORF (Fig. 3C). To account for reinitiation-independent translation initiation directly from the downstream start-codons we used the construct lacking the upstream ATG (Table 3). Only ~10% of fluorescence could be recovered in both cases due to reinitiation (Fig. 3D) which was consistent with the requirement of Shine-Dalgarno motif inside the upstream ORF previously reported to improve the reinitiation at downstream ORF by ~tenfold^10, 11^. Such ORF arrangement is likely not to find a common usage in peptide-encoding due to the rather narrow range of allowed sequence variability within the context of short peptide-coding ORF.

**Supplementary note 4** Control of cleavage and cyclization by adjustment of trypsin/peptide-substrate ratio

Both linear (wt N-/C-termini: SFTI[1,14]) and cyclic form of SFTI exercise their trypsin inhibitory activity primarily through the Thr-4 - Ile-7 loop presenting Lys-5 to the active site of trypsin ^12^. Double proline motif and intra-loop H-bonds ^13^ contribute entropically to the tight inhibitor binding ^12^ restricting the flexibility of the reactive loop. Therefore, cleavage of scissile Ser-6/Lys-5 bond proceeds slowly and eventually equilibrates with its resynthesis. As reported previously, the resynthesis reaction for SFTI is thermodynamically favoured ^14^ and if initiated at equal ratio of trypsin to circularly permuted SFTI[6,5] equilibrates within one hour at ~10-fold excess of cyclic SFTI over its linear form ^15^. Fig. 5A shows the trypsin-assisted cyclization of purified disulfide-bonded SFTI-RGS taken at equimolar ratio approaches the equilibrium after 2 h yielding 64% of cyclic SFTI, 16% of linear excision product (linear-SFTI[6,5]) and ~20% of double-cleaved specie with extra broken bond at Arg-2^14^ as judged by LC-MS/MS analysis (Fig. 3B). After additional 16 h incubation the fraction of cyclic SFTI increases only slightly to 66% while the amount of linear SFTI declines to 6% with concomitant increase in the double-cleaved SFTI fraction to 28% (Fig. 5A,B). If a ten-fold excess of fusion peptide over trypsin was used in the experiment it yielded mostly linear SFTI[6,5] after a 2 h incubation (Fig. 5B). This suggests that after the initial cleavage of SFTI-RGS both products dissociate from trypsin prior to rebinding of linear SFTI[6.5] to form a productive enzyme-inhibitor complex where the resynthesis of scissile bond can take place. Since its productive rebinding according to “Laskowski mechanism”^16^ is preceded by the rapidly equilibrating “loose” complex step the excess of initial RGS-fusion precursor delays the accumulation of inhibitory complex by competing with lin-SFTI[6,5] for rebinding.

To avoid double-cleavage of SFTI, we replaced Arg-2 with Gln based on the previous study^17^ demonstrating high tolerance of this position to various substitutions. Indeed, the resulting R2Q mutant had similar *K*_i_ for trypsin as the native counterpart (Fig. 6B). Interestingly, while affecting trypsin efficiency very moderately, R2Q mutation shifted the equilibrium within the inhibitory complex away from the bond resynthesis resulting in a decreased proportion of cyclized product (Fig. 5A,B). Chemically synthesized cyclic SFTI turned out to be the most potent trypsin inhibitor featuring *K*_i_ ~ 66 pM most likely due to a faster onset of equilibrium and higher resistance of the constrained cyclic backbone to secondary cleavage (Fig. 6A,B).

**Supplementary note 5** AC-assisted expression, oxidative folding and cyclization of macrocyclic trypsin inhibitor MCoTI

Synthetic chemically cyclized McoTI was shown to undergo efficient oxidative folding ^18, 19^ forming the on-path native-like intermediate harboring two disulfide bonds where the third disulfide bond closes the cyclotide’s embedded ring yielding the final native product (Fig. 3f). Native backbone cyclization proceeds via the formation of loop-6 in transpeptidation reaction mediated by asparagine endopeptidase (AEP)^20^ similar to SFTI and some other cyclotides. Additionally, similar to SFTI, circularly permuted MCoTI opened at scissile Lys6-Ile7 bond (Fig. 3f) could be cyclized by trypsin with ~9-10-fold excess of cyclic over linear form at equilibrium^21^. Following co-translational capture on AC-coated resin, washing and complete on-resin reduction (Fig. 8A), on-resin oxidative folding was performed for 12 h in the buffer containing 10 mM GSH. Acquisition of three disulfide bridges by predominant peptide fraction as well as the presence of two disulfide-bridged intermediate^18^ was confirmed by LC-MS (Fig. 8B). In order to confirm the formation of native structure, wild type and circularly permuted McoTI-RGS fusion peptides were pre-incubated with trypsin and assayed for trypsin inhibitory activity. This allowed RGS-tag removal from prmMcoTI-RGS followed by its subsequent rebinding with trypsin and equilibrium resynthesis of the scissile bond.

**Supplementary note 6** Cyclization, oxidative folding and structural characterization of kalata B1 produced in AC-assisted Ec CFS

Native processing and cyclization of kalata B1 is mediated by asparaginyl endopeptidase (AEP) via primary cleavage at N↓GL, release of C-terminal part followed by docking of N-terminal GL-motif and transpeptidation reaction^22, 23^. To cyclize *in vitro* produced kalata B1, we introduced a Gly-Leu-linker between kalata B1 and RGS-peptide to mediate C-terminal processing by a recombinantly expressed asparaginyl endopeptidase from *Oldenlandia affinis* (OaAEP1b)^24^ while compatible N-terminus was generated as a result of co-translational cleavage of the translation leader by TVMV (Fig. 9A,B). Kalata B1-RGS was expressed in AC-assisted Ec CFS, fully reduced with 50 mM DTT in 0.1 M NH_4_HCO_3_, pH 8.5 ^25^ and subjected to RGS-tag cleavage and cyclization by incubation with 1/10 resin volume of OaAEP1b at 12 µg/mL in 50 mM NaCOOCH_3_, 50 mM NaCl, 1 mM EDTA, pH 5.0 for 22 h ^26^ (Fig. 8C,D). Prior to the above procedure, comparative quantitative analysis of kalata B1 samples eluted from the resin before or after reduction suggested the importance of reduction step for resolving of covalent aggregates (Fig. 7B). Cyclized kalata B1 was submitted to oxidative refolding with 5 mM GSH ^25^ in 50% isopropyl alcohol (v/v), 0.1 M NH_4_HCO_3_, pH 8.5 at room temperature overnight. Oxidized *in vitro* translated peptide and synthetic matured kalata B1 showed overlapping reverse phase HPLC elution profiles confirming the acquisition of native structure by the former peptide (Fig. 9C).

**Supplementary note 7** Structural and functional characterization of Arenicin-3 derivative AA139 produced in AC-assisted *E. coli* *in vitro* translation reaction

Antimicrobial peptide Arenicin-3 isolated from the lugworm *Arenicola marina* is a 21-residue amphipathic β-hairpin stabilized by two disulfide bridges^27^. A fusion of Arenicin-3 variant - AA139 with RGS sequence was expressed in AC-assisted Ec CFS and fully reduced on-resin followed by treatment with OaAEP1b (Supplementary note 6). Due to low efficiency of tag removal by OaAEP1b we resorted to chemical cleavage of the fusion precursor at Asn-Gly bond with hydroxylamine^28^. To this end, on-resin reduced AA139-RGS was subjected to oxidative folding in the presence of 10 mM GSH followed by incubation of resin-immobilized peptide with 10 resin volumes of 2 M hydroxylamine at 45°C for 4 h in the buffer containing 15 mM Tris buffered with LiOH to pH 9.3^29^ (Fig. 10A). Interestingly, the second Asn-Gly bond in the context of disulfide-constrained peptide’s core was resistant to chemical cleavage. The correct mass of oxidized peptide cleaved off the beads by hydroxylamine was confirmed by LC-MS (Fig. 10B). Arenicin-3 is known to negatively affect bacterial translation machinery. Indeed, we found the molar yield of AA139-RGS translated in S30 extract was significantly lower compared to other peptides (Table 1). Intriguingly, translation in a minimal fully reconstituted PURE system was unaffected. To increase the yield of AA139 we performed its translation in the Continuous Exchange (CECFS) format (Fig. 4). AC-coated resin was also introduced into the feeding solution compartment at 1/5^th^ of its reaction volume to capture peptides crossing the dialysis membrane. After completion of translation AC-resins from both compartments were combined and peptide expression yield was quantified by AC-assay. The total yield was 48 μg/mL exceeding the yield of the batch reaction by ~4-5-fold. The resulting product was tested for its antibacterial activity^30^ in a Minimal Inhibitory Concentration assay^31^. The activity of in vitro translated peptide was found to be close to the activity of the synthetic analogs confirming formation of the native structure (Fig. 10C).

**Supplementary note 8** Structural and functional characterization of Holocyclotoxin-1 (HT-1) RGS-fusion produced in AC-assisted Ec CFP

Holocyclotoxins isolated from *Ixodes holocyclus* trigger host paralysis through interference with neurotransmission ^32^ via a yet uncharacterized mechanism. HT-1 does not share structural homology with any known cysteine-rich peptides, featuring four disulfide bonds (Fig. 11A). Three of them contribute to maintenance of the inhibitory cysteine knot structure while the fourth disulfide bond staples the N-terminus to one of the loops increasing the overall peptide’s rigidity^33^. HT-1 chemical synthesis is challenging as it requires solid-phase synthesis of two segments followed by their native chemical ligation^33^. Heterologous production in the prokaryotic system was unsuccessful while HT-1 fusion protein produced in yeast required an additional refolding step^34^. Based on the notion that physiological effect of holocyclotoxins was believed to be exerted through modulation of calcium channel activity^32^, we tested the effect of the available synthetic holocyclotoxins on the previously designed DHFR-calmodulin chimeric biosensor^35^. The DHFR activity of this biosensor is enhanced >200 fold by addition of M13 peptide derived from calmodulin-binding domain of skeletal muscle myosin light chain kinase. Synthetic HT-1 effectively prevented activation of DHFR-calmodulin chimera by the calmodulin-binding peptide most likely by targeting the calmodulin part of the biosensor (data not shown). We used this assay to assess the activity of *in vitro* expressed HT-1. HT-1-RGS produced in resin-assisted Ec CFS was reduced on-resin and subjected to oxidative folding either with 13 µM GSH for 96 h at 4°C accordingly to previous report ^33^ or in the presence of 10 mM GSH for 16 h at 25°C in 0.1 M NH_4_HCO_3_, pH 8.2. LC-MS analysis of peptide eluted from the resin with 0.2% TFA in the latter case revealed the presence of homogeneous products with 4 disulfide bonds (Fig. 11B). Colorimetric DHFR activity assay was performed as described before^35^ by monitoring the decrease in absorbance of NADPH at 340 nm at 25ºC in 1 mL of 20 mM NaCl, 20 mM KH_2_PO_4_ pH 7.5 buffer containing 10 nM of chimeric DHFR-calmodulin sensor, 0.2 µM of M13 calmodulin binding peptide, 80 µM NADPH, 67 µM dihydrofolic acid, 100 µM CaCl_2_ and 2 µM of either reduced or oxidized HT-1-RGS fusion. The changes in absorbance were recorded on Cary 50 UV-Vis spectrophotometer (Varian Inc.). Only oxidized *in vitro* translated HT-1 inhibited the biosensor confirming the installation of native disulfide bridges critical for HT-1 functionality (Fig. 11C).

**Supplementary note 9** Structural and functional characterization of *in vitro* produced Pn3a and Dc1a toxins

Isolated from venoms of the South American tarantula *Pamphobeteus nigricolor* and the desert bush spider *Diguetia canities*, respectively, µ-theraphotoxin-Pn3a and µ-diguetoxin-Dc1a (hereafter Pn3a and Dc1a) are potent and selective inhibitors of mammalian or insect voltage-gated sodium channels, respectively. Both Pn3a and Dc1a have been extensively studied as models for the development of analgesics^36^ or bio-insecticides^37^, respectively. Pn3a contains three disulfide bridges arranged into classical inhibitory cystine knot. Dc1a displays marked difference from all other knottins as it possesses an additional disulfide staple in the selectivity-determining hairpin loop^38^. While Dc1a could be produced in active form in *E. coli* periplasm^37^, production of correctly folded Pn3a is challenging using both recombinant and solid-phase synthesis (I. Vetter, personal communication 2019). Following expression in AC-assisted Ec CFS, full on-bead reduction and oxidative folding, Pn3a and Dc1a were eluted from the resin followed by tag-removal via Carboxypeptidase Y or Thrombin cleavage, respectively (Fig. 12B,E). LC-MS analysis confirmed the accumulation of the major oxidized Pn3a product lacking three native C-terminal amino acids (Fig. 12C). The latter was inactive in electrophysiological assay, as well as was the synthetic native analogue subjected to the same treatment with Carboxypeptidase Y in parallel (Fig. 12C). However, the overlapping co-elution profiles of the truncated variants of cell-free produced and synthetic peptides (Fig. 12D) confirmed the formation of native structure. Noteworthy, the critical importance of native C-terminus for Pn3a functionality was confirmed in the recent study^39^.

As for Dc1a, its recently published complex with the channel receptor^40^ suggested an outward projection of peptide’s C-terminus with the last critical contact to the receptor made by the penultimate aspartate. Therefore, we designed a Dc1a expression construct featuring a longer spacer separating Dc1a from RGS-tag (Fig. 12E). Following thrombin cleavage, Dc1a harboring GTGSGGR sequence on its C-terminus demonstrated insecticidal activity similar to the synthetic wild-type toxin, thereby confirming formation of the native structure (Fig. 12G).

**Supplementary note 10** Generic approach to removal of C-terminal RGS-tag

Based on a broad literature review and a search on MEROPS peptidase database (https://www.ebi.ac.uk/merops/), thrombin and carboxypeptidase A were identified as the best suitable proteases for the removal of C-terminal tag. Thrombin is a trypsin-like serine proteinase which preferentially cleaves at Arg-Gly bonds^41^ or Gly-Arg-||-Gly^42^ perfectly matching the sequence motif adjacent to RGS-tag. Critical importance of the flexibility in the cleavage region for the effective cleavage by thrombin and other proteases^43^ should result in effective cleavage of RGS-tag while secondary cleavage sites within the constrained core of the disulfide-bonded peptide would be expected to be essentially resistant to the cleavage. Thrombin cleavage reaction conditions were optimized using the synthetic SFTI-RGS fusion as a substrate. To this end the cleavage reaction was performed at different substrate to thrombin ratios at 25°C or 37°C for various length of time after which the cleavage products were purified, concentrated, desalted using Pierce™ C18 Spin Tips (ThermoFisher) and submitted to MALDI. As a result, the best cleavage was achieved at 10 pmol peptide/unit (equally 0.324 µg) of thrombin incubated at 37°C for at least 6 h (Fig. 13C).

Carboxypeptidase A (CPDA) was identified as the second protease candidate for RGS-tag removal. Unlike carboxypeptidase Y (CPDY), which indiscriminately removes C-terminal residues including proline^43, 44^, CPDA preferentially removes aromatic and bulky aliphatic side-chains with the exception of Arg, Lys or Pro^44, 45^. Therefore, RGS-tag comprises the native stopper for CPDA. Accordingly, using synthetic SFTI-RGS as a substrate peptide we optimized the cleavage conditions and identified three major truncation products corresponding to cleavage reaction stopped before Arg, Gly and Ser residues of RGS-tag, respectively, with the second being the most abundant cleavage product (Fig. 13B).

**Supplementary note 11** Reassignment of arginine and serine sense codons to p-azido-L-phenylalanine (AzF) and n-propargyl-L-lysine (PrK)

Previously we demonstrated that appropriately designed 2’-O-methylated antisense oligonucleotides can selectively sequester tRNAs decoding split-codon boxes of arginine and serine^46^. Here we screened an additional set of antisense oligonucleotides against the former and identified the novel oligonucleotide candidate termed R7 characterized by higher selectivity and sequestration efficiency towards tRNA^Arg^CCU compared to the previously developed R2^46^. We also demonstrated that newly identified R7 could be combined with previously selected M5-1 oligonucleotide that targets tRNA^Ser^GCU (Table 4). The oligonucleotide combination can simultaneously sequester both tRNAs directly in S30 *E. coli* lysate thereby liberating AGG- and AGT-codons. The orthogonal azidophenylalanine tRNA synthetase (AzFRS.2.t1) ^47^ and chimeric pyrrolysine tRNA synthetase (chPylRS)^48^ (Table 6) were expressed in *E. coli* and purified by Ni^2+^ affinity chromatography and gel filtration. We constructed both ACU and CCU anticodon versions for several tRNA substrate candidates of each enzyme, previously selected from a pool of efficient orthogonal amber-codon suppressors^49, 50^. All respective tRNA-anticodon/synthase combinations were evaluated for orthogonality and incorporation efficiency towards AzF or PrK in codon-reassigned Ec CFS (data not shown). We identified the two most efficient tRNA-anticodon/RS pairs: **tRNA**^AzF4^CCU/AzFRS.2.t1 and **tRNA**^PylO2^ACU/chPylRSAF (Table 5). These tRNAs were co-translationally charged with AzF and PrK that enabled incorporation of AzF and PrK replacing cysteine 3 and 11, respectively, in SFTI-RGS peptide. The modified peptide containing two click amino acid moieties was subjected to macrocyclization through the copper-catalyzed azide-alkyne cycloaddition performed either on AC resin or in solution following peptide elution. The azide-alkyne cycloaddition conditions were optimized using 3-azide-7-hydroxycoumarine (Jena Bioscience) as a fluorogenic reporter of the click-reaction. BTTP (Albert Einstein College of Medicine) was selected as the optimal Cu^+1^ chelating agent.

**Supplementary methods** Assembly of resin-assisted translation, conditions for refolding

**A. Preparation of affinity-clamp(AC)-coated resin**

1. The affinity clamp protein harboring C-terminal cysteine (Table 6) is adjusted to 10 mg/ml in a coupling buffer (50 mM Tris-HCl, 5 mM EDTA, TCEP*, pH 8.5) and added to saturation to UltraLink® iodoacetyl resin (ThermoFisher, #53155) pre-washed with the coupling buffer.

* pH of TCEP is to be adjusted to 8.0 with KOH prior to buffer formulation

2. Following 1 h incubation at room temperature with mild agitation the resin is settled, washed with a coupling buffer and blocked with 50 mM L-cysteine (pH 8.0) for 30 min.

3. The AC-coated resin is washed with 1 M NaCl and PBS and stored as 50% (v/v) suspension in PBS containing 0.1 mg/ml BSA and 2 mM NaN_3_ at 4°C.

**B. Preparation of *E. coli* S30 cell extract (from Schwarz et al.^51^ with minor modifications):**

| **S30 extract buffers**: | S30A*  (~2 L) | S30B*  (~0.1L) | | S30C*  (~8-10L) |
| --- | --- | --- | --- | --- |
| Components: |  |  |  |  |
| Tris-acetate pH8.2 (mM) | 10 | 10 | | 10 |
| Mg(OAc)_2_ (mM) | 14 | 14 | | 14 |
| KCl (mM) | 0,6 | 0,6 | | - |
| KOAc (mM) | - | - | | 0,6 |
| 2-mercaptoethanol (mM) | 6 | - | | - |
| DTT (mM) | - | 1 | | 0,5 |
| PMSF (mM) | - | 0,1 | | - |
| * prepare x50 stocks for S30AB and S30C (Tris/Mg/K) | | | | |
| **cultivation media** **TBGG*** (1 L) | | |  |  |
| Trypton | 12 g | |  |  |
| Yeast Extract | 24 g | |  |  |
| Glycerol | 8 ml | |  |  |
| Glucose | 1 g | |  |  |
| KH2PO4 | 2,31 g | |  |  |
| K2HPO4 | 12,54 g | |  |  |
| * prepare x2 concentrate and filter through 0.22 μm by passing 1V of x2 TBGG and 1V of water to obtain x1 sterile TBGG | | |  |  |

1. Overnight culture of BL21(DE3) *E. coli* is inoculated into 5 L TBGG media at 1:100 dilution, distributed over 6 conical 5L flasks (~850ml per each) and grown at 37°C with agitation.

2. In ~3-4 h, after reaching the log phase, cell culture is pooled and rapidly cooled down with 5x200 ml -80-pre-frozen packs of LB broth to yield 6 L of final culture volume.

3. Following complete dissolution of frozen LB packs, cells are pelleted and washed twice with 1L of pre-chilled S30A buffer (every time cells were spun for 15 min at 2.5kg at 4°C).

4. The cell pellet after the final wash is resuspended in 2V (vol/wt) of S30B buffer, and cells are disrupted by fluidic continuous flow disruption (Constant Systems CF1) with 20 kpsi at 4°C, followed by two consecutive centrifugation steps of the cell homogenate for 30 min at 30kg at 4°C while collecting top 3/4^th^ of supernatant every time.

5. Final supernatant is pooled and adjusted to 0.4 M with 5 M NaCl followed by 45 min incubation at 42° in a water bath.

6. Following incubation the cell homogenate is transferred to dialysis tubing with 12-14 kDa cutoff (Spectrum^TM^ Labs) and dialyzed for 2 h against 4 L of cold S30C buffer at 4°C followed by ON dialysis against the same volume of fresh buffer.

7. Following ON dialysis the extract is centrifuged for 30 min at 30kg at 4°C, top ¾ of supernatant is collected, aliquoted, frozen in LN_2_ and stored at -80°.

**C. Assembly of AC-resin-assisted Ec CFS reaction**

C-1 Preparation of affinity-resin for translation reaction

1. The resin amounts corresponding to 20 μl and 40 μl of 50% (vl/vl) suspension of Strep-Tactin(ST)-and Affinity-clamp(AC)-coated resins are used to setup 100 μl of resin-assisted translation reaction.

Ligand binding capacities of AC- and ST-coated resins and the resin amount per reaction:

| Affinity resin: | Binding capacity (nmol of ligand) per 1 ml of settled resin (2 ml of 50%suspension) | V (μl of 50% suspension) per 100 μl of translation reaction |
| --- | --- | --- |
| AC-coated resin | ~27 | 40 |
| ST-coated resin | ~50 | 20 |

2. The required volume of 50% resin suspension is transferred into a filter bottom tube, followed with a quick 1 kg spin to drain the liquid and with 6 continuous washes of water. Following the final spin at 2.5 kg the resin is equilibrated with 1V of reaction solution mixed of 35% (vl/vl) of extract buffer and 40% (vl/vl) of x2.5 Feeding solution (refer to a Table below) in two consecutive loadings: following centrifugation at 2.5 kg for 1 min after the first loading, the resin is stored with a second portion of reaction solution at RT while transcription-translation mixture is prepared.

C-2 Assembly of transcription-translation reaction

1. The reaction constituents are mixed on ice as indicated in a Table below:

(For translation of disulfide-constrained proteins DTT concentration in translation reaction is adjusted to 10 mM by addition of extra 7.5 mM DTT)

Standard protocol for the assembly of S30 extract-based transcription-translation reaction:

| Major constituents: | Components: | Concentration: | Reaction assembly: |
| --- | --- | --- | --- |
| S30 extract | Tris-acetate pH 8.2 | 10 mM | 35 % (v/v) |
|  | Mg(OAc)2 | 14 mM |  |
|  | KOAc | 0.6 mM |  |
|  | DTT | 0.5 |  |
| x 2.5 Feeding Solution | Hepes-KOH pH7.6 | 236 mM | 40 % (v/v) |
|  | Mg(OAc)2 | 12.5 mM |  |
|  | KOAc | 375 mM |  |
|  | PEG 8000 | 5 % |  |
|  | NaN3 | 5 mM |  |
|  | Tween 20 | 0.015% |  |
|  | DTT | 5 mM |  |
|  | cOmplete™ EDTA-free, Protease inhibitors (Roche) | x 2.5 |  |
|  | Folinic acid | 0.25 mg/ml |  |
|  | rNTPs each | 2 mM (each) |  |
|  | ATP (extra) | 1 mM |  |
|  | Acetyl phosphate | 38 mM |  |
|  | Creatine Phosphate | 68 mM |  |
|  | 20 amino acids | 1.25 mM (each) |  |
|  | R,C,W,D,G (extra) | 2.5 mM (each) |  |
| Enzymes | T7 polymerase | stock | 0.05 mg/ml |
|  | Creatine phosphokinase | stock | 45 U/ml |
|  | TVMV-protease | stock | 0.05 mg/ml |
| Template | Plasmid DNA | 0.2-0.4 μM | 20-40 nM |

2. Following the assembly of reaction mixture the affinity resin is drained from the equilibration buffer by centrifugation for 1 min at 2.5 kg and semi-dried resin is directly emptied into the reaction mixture.

3. Resin-assisted reaction mixtures are incubated in round-bottom tubes in a thermomixer (Eppendorf) for 4 h at 1050 rpm at 32°C and 28(25)°C for peptide and protein translation, respectively.

4. After a completion of translation reactions, the reaction mixtures are transferred to filter-bottom tubes, flow-throughs are drained and resins are washed by six alternating sessions of either water and WB (refer to Table below) for immobilized peptides or WB and NB for immobilized proteins, followed by three continuous final washes with water or NB, respectively.

**D. Refolding Procedure:**

Buffer compositions:

| label: | buffer name: | buffer composition: |
| --- | --- | --- |
| WB | wash buffer (AC-resin) | 50 mM TrisHCl, 500 mM NaCl, 0.1% Tween 20, pH 7.5 |
| WB1 | wash buffer (ST-resin) | 20 mM Tris-HCl, 100 mM NaCl, 0.05% Tween 20, pH 7.5 |
| NB | neutral buffer | 20 mM TrisHCl, 20 mM NaCl, pH 7.5 |

For refolding procedure 40 μl of respective buffer containing denaturant was used per resin amount corresponding to 6 μl and 12.5 μl of 50% (v/v) suspensions of Strep-Tactin(ST)- and Affiniy-clamp(AC)-coated resins, respectively.

Translation and refolding of disulfide-constrained peptides in AC-assisted Ec CFS

| Process: | Conditions: | Time | Agitation  (rpm) | T°C |
| --- | --- | --- | --- | --- |
| Translation | AC-assisted CFS, standard | 3 h | 1200 | 32 |
| Washing x6 | WB/NB/WB/NB/WB/NB | na | na | RT |
| Washing x3 | AQ | na | na |  |
| Full reduction | 50 mM DTT, 0.1 M NH_4_HCO_3_, pH 8.5 | 30 min | 1400 |  |
| Washing x3 | 0.1 M NH_4_HCO_3_, pH 8.5 | na | na |  |
| Oxidative folding | 10 mM GSH, 0.1M NH_4_HCO_3_, pH 8.5 | 12-48 h | 1400 |  |
| Washing x3 | 0.1 M NH4HCO3, pH 8.5 | na | na |  |
| Washing x3 | AQ | na | na |  |
| Elution x3 | 0.2% TFA | na | 1400 |  |

Translation and refolding of disulfide-constrained proteins in AC-assisted Ec CFS:

| Process: | Conditions: | Time | Agitation  (rpm) | T°C |
| --- | --- | --- | --- | --- |
| Translation | AC-assisted CFS, + 7.5 mM DTT,  50% S30 extract + 50% S30C buffer | 4 | 1200 | 25 |
| Washing x6 | WB/NB/WB/NB/WB/NB | na | na | RT |
| Washing x3 | NB | na | na |  |
| Refolding  step 1 | 2 M Gdn, 100 mM DTT, 50 mM TrisHCl, 1M NaCl, pH7.5 | 2 h | 1400 |  |
| Refolding  step 2 | 1 M Gdn, 10 mM GSH, 50 mM TrisHCl, 500 mM NaCl, pH7.5 | 16 h | 1400 |  |
| Dilution 1:2 | NB | 1 h | 1400 |  |
| Dilution 1:2 | NB | 1 h | 1400 |  |
| Washing x6 | NB | na | na |  |

Translation and refolding of proteins lacking disulfide bonds in AC- or ST-assisted Ec CFS:

| Process: | Conditions: | Incub.  time | Agitation  (rpm) | T°C |
| --- | --- | --- | --- | --- |
| Translation | ST-/AC- assisted CFS  50% S30 extract + 50% S30C buffer | 3 h | 1200 | 28 |
| Washing x6  (ST-resin) | WB1/NB/WB1/NB/WB1/NB | na | na | RT |
| Washing x6  (AC-resin) | WB/NB/WB/NB/WB/NB | na | na |  |
| Washing x3 | NB | na | na |  |
| Refolding  (ST-resin) | 1.6 M Gdn, 20 mM TrisHCl, 2.5 mM DTT, 20 mM NaCl, 0,125% Tween 20, pH7.5 | 2 h | 1400 |  |
| Refolding  (AC-resin) | 2 M Gdn, 20 mM TrisHCl, 2.5 mM DTT, 1 M NaCl, 0,125% Tween 20, pH7.5 | 6 h | 1400 |  |
| Dilution 1:2 | NB | 1 h | 1400 |  |
| Dilution 1:2 | NB | 1 h | 1400 |  |
| Washing x6 | NB | na | na |  |
| Elution x2  (ST-resin) | 50 mM biotin, 100 mM Tris-HCl pH 8.5, 150 mM NaCl, 0.25 mM EDTA | 15 min | 1400 | 30 |

References

1. Avrutina O*, et al.* Trypsin inhibition by macrocyclic and open-chain variants of the squash inhibitor MCoTI-II. *Biol Chem* **386**, 1301-1306 (2005).

2. Stanger K, Maurer T, Kaluarachchi H, Coons M, Franke Y, Hannoush RN. Backbone cyclization of a recombinant cystine-knot peptide by engineered Sortase A. *Febs Lett* **588**, 4487-4496 (2014).

3. Guo S, Herzig V, King GF. Dipteran toxicity assays for determining the oral insecticidal activity of venoms and toxins. *Toxicon* **150**, 297-303 (2018).

4. Herzig V, Hodgson WC. Neurotoxic and insecticidal properties of venom from the Australian theraphosid spider Selenotholus foelschei. *Neurotoxicology* **29**, 471-475 (2008).

5. Appleman JR, Beard WA, Delcamp TJ, Prendergast NJ, Freisheim JH, Blakley RL. Unusual transient- and steady-state kinetic behavior is predicted by the kinetic scheme operational for recombinant human dihydrofolate reductase. *J Biol Chem* **265**, 2740-2748 (1990).

6. Li J*, et al.* Dissecting limiting factors of the Protein synthesis Using Recombinant Elements (PURE) system. *Translation (Austin)* **5**, e1327006 (2017).

7. Stogbauer T, Windhager L, Zimmer R, Radler JO. Experiment and mathematical modeling of gene expression dynamics in a cell-free system. *Integr Biol (Camb)* **4**, 494-501 (2012).

8. Cui Z, Stein V, Tnimov Z, Mureev S, Alexandrov K. Semisynthetic tRNA complement mediates in vitro protein synthesis. *Journal of the American Chemical Society* **137**, 4404-4413 (2015).

9. Salgado H, Moreno-Hagelsieb G, Smith TF, Collado-Vides J. Operons in Escherichia coli: genomic analyses and predictions. *Proc Natl Acad Sci U S A* **97**, 6652-6657 (2000).

10. Osterman IA, Evfratov SA, Sergiev PV, Dontsova OA. Comparison of mRNA features affecting translation initiation and reinitiation. *Nucleic Acids Res* **41**, 474-486 (2013).

11. Spanjaard RA, van Duin J. Translational reinitiation in the presence and absence of a Shine and Dalgarno sequence. *Nucleic Acids Res* **17**, 5501-5507 (1989).

12. Luckett S*, et al.* High-resolution structure of a potent, cyclic proteinase inhibitor from sunflower seeds. *J Mol Biol* **290**, 525-533 (1999).

13. de Veer SJ*, et al.* Engineered protease inhibitors based on sunflower trypsin inhibitor-1 (SFTI-1) provide insights into the role of sequence and conformation in Laskowski mechanism inhibition. *Biochem J* **469**, 243-253 (2015).

14. Colgrave ML, Korsinczky MJ, Clark RJ, Foley F, Craik DJ. Sunflower trypsin inhibitor-1, proteolytic studies on a trypsin inhibitor peptide and its analogs. *Biopolymers* **94**, 665-672 (2010).

15. Marx UC*, et al.* Enzymatic cyclization of a potent bowman-birk protease inhibitor, sunflower trypsin inhibitor-1, and solution structure of an acyclic precursor peptide. *J Biol Chem* **278**, 21782-21789 (2003).

16. Luthy JA, Praissman M, Finkenstadt WR, Laskowski M, Jr. Detailed mechanism of interaction of bovine -trypsin with soybean trypsin inhibitor (Kunitz). I. Stopped flow measurements. *J Biol Chem* **248**, 1760-1771 (1973).

17. Hilpert K, Hansen G, Wessner H, Volkmer-Engert R, Hohne W. Complete substitutional analysis of a sunflower trypsin inhibitor with different serine proteases. *J Biochem* **138**, 383-390 (2005).

18. Cemazar M, Daly NL, Haggblad S, Lo KP, Yulyaningsih E, Craik DJ. Knots in rings. The circular knotted protein Momordica cochinchinensis trypsin inhibitor-II folds via a stable two-disulfide intermediate. *J Biol Chem* **281**, 8224-8232 (2006).

19. Thongyoo P, Tate EW, Leatherbarrow RJ. Total synthesis of the macrocyclic cysteine knot microprotein MCoTI-II. *Chem Commun (Camb)*, 2848-2850 (2006).

20. Mylne JS*, et al.* Cyclic peptides arising by evolutionary parallelism via asparaginyl-endopeptidase-mediated biosynthesis. *Plant Cell* **24**, 2765-2778 (2012).

21. Thongyoo P, Roque-Rosell N, Leatherbarrow RJ, Tate EW. Chemical and biomimetic total syntheses of natural and engineered MCoTI cyclotides. *Org Biomol Chem* **6**, 1462-1470 (2008).

22. Bernath-Levin K*, et al.* Peptide macrocyclization by a bifunctional endoprotease. *Chem Biol* **22**, 571-582 (2015).

23. Gillon AD, Saska I, Jennings CV, Guarino RF, Craik DJ, Anderson MA. Biosynthesis of circular proteins in plants. *Plant J* **53**, 505-515 (2008).

24. Conlan BF, Colgrave ML, Gillon AD, Guarino R, Craik DJ, Anderson MA. Insights into processing and cyclization events associated with biosynthesis of the cyclic Peptide kalata B1. *J Biol Chem* **287**, 28037-28046 (2012).

25. Daly NL, Clark RJ, Craik DJ. Disulfide folding pathways of cystine knot proteins. Tying the knot within the circular backbone of the cyclotides. *J Biol Chem* **278**, 6314-6322 (2003).

26. Harris KS*, et al.* Efficient backbone cyclization of linear peptides by a recombinant asparaginyl endopeptidase. *Nat Commun* **6**, 10199 (2015).

27. Rabanal F, Cajal Y. Therapeutic Potential of Antimicrobial Peptides. In: *New Weapons to Control Bacterial Growth* (eds Villa TG, Vinas M). Springer International Publishing (2016).

28. Bornstein P, Balian G. [14] Cleavage at AsnGly bonds with hydroxylamine. In: *Methods in Enzymology*). Academic Press (1977).

29. Simpson RJ. Cleavage of asn-gly bonds by hydroxylamine. *CSH Protoc* **2007**, pdb prot4697 (2007).

30. Wiegand I, Hilpert K, Hancock RE. Agar and broth dilution methods to determine the minimal inhibitory concentration (MIC) of antimicrobial substances. *Nat Protoc* **3**, 163-175 (2008).

31. Wikler MA. Methods for dilution antimicrobial susceptibility tests for bacteria that grow aerobically : approved standard. *CLSI (NCCLS)* **26**, M7-A7 (2006).

32. Chand KK*, et al.* Tick holocyclotoxins trigger host paralysis by presynaptic inhibition. *Sci Rep* **6**, 29446 (2016).

33. Vink S, Daly NL, Steen N, Craik DJ, Alewood PF. Holocyclotoxin-1, a cystine knot toxin from Ixodes holocyclus. *Toxicon* **90**, 308-317 (2014).

34. Karbanowicz T, Dover E, Mu X, Tabor A, Rodriguez-Valle M. Extracellular expression of the HT1 neurotoxin from the Australian paralysis tick in two Saccharomyces cerevisiae strains. *Toxicon* **140**, 1-10 (2017).

35. Guo Z*, et al.* Generalizable Protein Biosensors Based on Synthetic Switch Modules. *J Am Chem Soc*, (2019).

36. Deuis JR*, et al.* Pharmacological characterisation of the highly NaV1.7 selective spider venom peptide Pn3a. *Sci Rep* **7**, 40883 (2017).

37. Bende NS*, et al.* A distinct sodium channel voltage-sensor locus determines insect selectivity of the spider toxin Dc1a. *Nat Commun* **5**, 4350 (2014).

38. Bende NS*, et al.* The insecticidal spider toxin SFI1 is a knottin peptide that blocks the pore of insect voltage-gated sodium channels via a large beta-hairpin loop. *FEBS J* **282**, 904-920 (2015).

39. Sharma G, Deuis JR, Jia X, Mueller A, Vetter I, Mobli M. Recombinant production, bioconjugation and membrane binding studies ofPn3a, a selective NaV1.7 inhibitor. *Biochem Pharmacol* **181**, 114148 (2020).

40. Shen H*, et al.* Structural basis for the modulation of voltage-gated sodium channels by animal toxins. *Science* **362**, (2018).

41. Lundblad RL, Kingdon HS, Mann KG. [14] Thrombin. In: *Methods in Enzymology*). Academic Press (1976).

42. Chang JY. Thrombin specificity. Requirement for apolar amino acids adjacent to the thrombin cleavage site of polypeptide substrate. *Eur J Biochem* **151**, 217-224 (1985).

43. Robertson AL*, et al.* Protein unfolding is essential for cleavage within the alpha-helix of a model protein substrate by the serine protease, thrombin. *Biochimie* **122**, 227-234 (2016).

44. Breddam K. Serine carboxypeptidases. A review. *Carlsberg Research Communications* **51**, 83 (1986).

45. Tanco S*, et al.* Proteome-derived peptide libraries to study the substrate specificity profiles of carboxypeptidases. *Mol Cell Proteomics* **12**, 2096-2110 (2013).

46. Cui Z, Wu Y, Mureev S, Alexandrov K. Oligonucleotide-mediated tRNA sequestration enables one-pot sense codon reassignment in vitro. *Nucleic Acids Res* **46**, 6387-6400 (2018).

47. Amiram M*, et al.* Evolution of translation machinery in recoded bacteria enables multi-site incorporation of nonstandard amino acids. *Nat Biotechnol* **33**, 1272-1279 (2015).

48. Bryson DI, Fan C, Guo LT, Miller C, Soll D, Liu DR. Continuous directed evolution of aminoacyl-tRNA synthetases. *Nat Chem Biol* **13**, 1253-1260 (2017).

49. Cui Z*, et al.* Combining Sense and Nonsense Codon Reassignment for Site-Selective Protein Modification with Unnatural Amino Acids. *ACS Synth Biol* **6**, 535-544 (2017).

50. Fan C, Xiong H, Reynolds NM, Soll D. Rationally evolving tRNAPyl for efficient incorporation of noncanonical amino acids. *Nucleic Acids Res* **43**, e156 (2015).

51. Schwarz D*, et al.* Preparative scale expression of membrane proteins in Escherichia coli-based continuous exchange cell-free systems. *Nat Protoc* **2**, 2945-2957 (2007).
