# Supplementary material for: Towards a generic prototyping approach for therapeutically-relevant peptides and proteins in a cell-free translation system
Source: Nat Commun. 2022 Jan 11;13:260. doi: 10.1038/s41467-021-27854-9 (PMC8752827; doi:10.1038/s41467-021-27854-9)
Supplement: Supplementary file 2 — Reporting Summary [file 41467_2021_27854_MOESM2_ESM.pdf]

nature portfolio

Corresponding author(s): Sergey Mureev, Kirill Alexandrov

Last updated by author(s): Nov 4, 2021

# Reporting Summary

Nature Portfolio wishes to improve the reproducibility of the work that we publish. This form provides structure for consistency and transparency in reporting. For further information on Nature Portfolio policies, see our Editorial Policies and the Editorial Policy Checklist.

## Statistics

For all statistical analyses, confirm that the following items are present in the figure legend, table legend, main text, or Methods section.

| n/a | Confirmed | |
|---|---|---|
| [ ] | [x] | The exact sample size (*n*) for each experimental group/condition, given as a discrete number and unit of measurement |
| [ ] | [x] | A statement on whether measurements were taken from distinct samples or whether the same sample was measured repeatedly |
| [x] | [ ] | The statistical test(s) used AND whether they are one- or two-sided<br>*Only common tests should be described solely by name; describe more complex techniques in the Methods section.* |
| [x] | [ ] | A description of all covariates tested |
| [x] | [ ] | A description of any assumptions or corrections, such as tests of normality and adjustment for multiple comparisons |
| [ ] | [x] | A full description of the statistical parameters including central tendency (e.g. means) or other basic estimates (e.g. regression coefficient) AND variation (e.g. standard deviation) or associated estimates of uncertainty (e.g. confidence intervals) |
| [x] | [ ] | For null hypothesis testing, the test statistic (e.g. *F*, *t*, *r*) with confidence intervals, effect sizes, degrees of freedom and *P* value noted<br>*Give P values as exact values whenever suitable.* |
| [x] | [ ] | For Bayesian analysis, information on the choice of priors and Markov chain Monte Carlo settings |
| [x] | [ ] | For hierarchical and complex designs, identification of the appropriate level for tests and full reporting of outcomes |
| [x] | [ ] | Estimates of effect sizes (e.g. Cohen's *d*, Pearson's *r*), indicating how they were calculated |

*Our web collection on statistics for biologists contains articles on many of the points above.*

## Software and code

Policy information about availability of computer code

| | |
|---|---|
| Data collection | Fluorescence-based kinetic assays (Affinity-Clamp assay, Trypsin inhibitory assay) - Tecan Infinite M1000 Pro plate reader software; Fluorescence end-point, fluorescence DHFR activity assay - BioTek Synergy 4 Multi-Mode Microplate reader software; DHFR-calmodulin biosensor colorimetric assay - Cary 50 UV-Vis spectrophotometer (Varian Inc.) Software; peptide peak intencity quantification - AB Sciex TripleTOF 5600, Analyst TF 1.6 software; 5800 MALDI-TOF/TOF Mass Spectrometer Data Explorer software; Applied Biosystems® ViiA™ 7 Real-Time PCR System default program |
| Data analysis | GraphPad Prism 8, Excel 2016 (version 2110), Image J 1.53k, Analyst TF 1.6 software (ABSCIEX, Canada), Data Explorer software (5800 MALDI-TOF/TOF Mass Spectrometer), |

For manuscripts utilizing custom algorithms or software that are central to the research but not yet described in published literature, software must be made available to editors and reviewers. We strongly encourage code deposition in a community repository (e.g. GitHub). See the Nature Portfolio guidelines for submitting code & software for further information.

## Data

Policy information about availability of data

All manuscripts must include a data availability statement. This statement should provide the following information, where applicable:

- Accession codes, unique identifiers, or web links for publicly available datasets
- A description of any restrictions on data availability
- For clinical datasets or third party data, please ensure that the statement adheres to our policy

The authors declare that the data supporting the findings of this study are available in this paper and its supplementary information files. Source data are provided with this paper.

A sequence of the affinity clamp (PDZ-fibronectin fusion protein) was extracted from the PDB file 3CH8. Sequences of the anti-HSA scFv and Fab antibody fragments were derived from the PDB file 5FUO. Sequences of the anti-IL-6 scFv, anti-IL-6 Fab, anti-IFNα-1b scFv and anti-IFNα-2a Fab were derived from the following PDB files: PDB:4CNI, PDB:4ZS7, PDB:3UX9, PDB:4YPG.

# Field-specific reporting

Please select the one below that is the best fit for your research. If you are not sure, read the appropriate sections before making your selection.

☒ Life sciences ☐ Behavioural & social sciences ☐ Ecological, evolutionary & environmental sciences

For a reference copy of the document with all sections, see nature.com/documents/nr-reporting-summary-flat.pdf

# Life sciences study design

All studies must disclose on these points even when the disclosure is negative.

**Sample size**

The sample size applies to representative sets of disulfide-constrained polypeptides used to demonstrate the broad applicability of the method for various peptide classes and for its benchmarking against commercial cell-free translation systems.  Seven peptides were selected to cover a range of molecular sizes and various disulfide connectivities. On another hand, the selection was based on high commercial value for some of them and/or on availabiliy of the methods for their activity assay. A set of 10 antibody fragments of different sizes and structural complexities as well as RBD of the SARS-CoV2 were selected based on availability of the respective ligands for pulldown functionality assay.

We did not do a particular estimation of sample size as this work is mostly about hypothesis verification at the first stage rather than correlation analysis which would demand the proper statistical measures.

We analyze whether the changes in two system parameters (one at a time) affect the protein solubility and activity in general rather than measuring the degree of this effect. We monitor different activities such as fluorescence and enzymatic activity within the same fusion molecule. We chose the protein fusion candidates that are expected to possess higher or lower sensitive to such changes based on the previous study. On another hand for purely technical reasons the range of suitable reporters is limited with the availability of convenient and fast assays. This is why we take advantage of fluorescence readout and DHFR activity which are analyzed simultaneously to optimize the method. We further validate this method on a larger subset of complex peptides and proteins for which the functionality estimation is either not trivial or time-consuming. However, we admit that it is difficult in the end to say whether the set of proteins and peptides tested is enough to claim generalization as one can always ask for more tests.

**Data exclusions**

no data was excluded from the analysis

**Replication**

Average values for the initial rates of DHFR-catalyzed NADPH-oxidation reaction and GFP fluorescence yields were derived using biological replicates of two or three independent translation experiments as specified in the figure legends. 90% of experiments were successful. Peptide quantification by Affinity-Clamp assay was performed in technical triplicates. All experiments were successful.

We used different batches of translation lysates and confirmed the observed effects. However, in one of three batches used for S30 lysate the refolding efficacy was distinctly lower than in the other two – we mention this observation in the manuscript and provide the rational for that based on some conclusions drawn in the course of study.

We chose the folding reporters to include the fast folding and slow folding reporter based on the previous study. We monitored the effect in pro- and eukaryotic translation system.

We monitored two independent activities within the same fusion molecule.

In the refolding experiment, when the activity of resin-immobilized protein is compared with the activity of the protein in denaturant-free buffer, refolding has to work against the slow protein dissociation process caused by denaturant thus providing stronger justification for the gain in protein activity.

The mentioned percent (10%) of unsuccessful experiments was accounted by some technical issues such as batch-to-batch variation in the quality of the translation extract used to constitute the translation reaction as well as in the batch-to-batch variation in the coupling efficiency between the affinity clamp and iodoacetyl sepharose. These either resulted in a poor general product yield or in a poor absolute protein capture. The protein reporters from unproductive translation sets were not analyzed further.

**Randomization**

N/A,  There are no different treatment groups of individuals. Control and experiment samples are identical by the origin.

**Blinding**

N/A, Parallel treatment procedures cannot be blindly assigned due to differences in appearance, color shading or viscosity

# Reporting for specific materials, systems and methods

We require information from authors about some types of materials, experimental systems and methods used in many studies. Here, indicate whether each material, system or method listed is relevant to your study. If you are not sure if a list item applies to your research, read the appropriate section before selecting a response.

## Materials & experimental systems

| n/a | Involved in the study | |
|---|---|---|
| ☐ | ☒ | Antibodies |
| ☒ | ☐ | Eukaryotic cell lines |
| ☒ | ☐ | Palaeontology and archaeology |
| ☐ | ☒ | Animals and other organisms |
| ☒ | ☐ | Human research participants |
| ☒ | ☐ | Clinical data |
| ☒ | ☐ | Dual use research of concern |

## Methods

| n/a | Involved in the study | |
|---|---|---|
| ☒ | ☐ | ChIP-seq |
| ☒ | ☐ | Flow cytometry |
| ☒ | ☐ | MRI-based neuroimaging |

# Antibodies

| | |
|---|---|
| Antibodies used | Primary mouse anti-GFP antibodies (dilution WB 1:1000) (#11814460001, Roche), secondary goat anti-mouse IgG (H+L) conjugated with HRP (dilution WB 1:5000) (Cat. No. G-21040, Life Technologies); Commercial anti SARS-COV2-RBD antibody (Sanyou Biopharmaceuticals, #AHA004) was used for pulldown assay as a ligand against immobilized SARS-CoV2-RBD - 50 µL of 10 µM solution was incubated with immobilized SARS-CoV2-RBD. |
| Validation | Anti-GFP antibody (11814460001, Roche) is tested for functionality and purity relative to a reference standard to confirm the quality of each new reagent preparation. (https://www.sigmaaldrich.com/US/en/product/roche/11814460001)<br>The antibody has been evaluated in the two component biosensor by competing with anti-RBD VHH part of the biosensor for binding the RBD.<br>anti-SARS-CoV2-RBD antibody: To analyze the binding of monoclonal anti-RBD antibody to RBM peptides in CuMVTT-DF, half-well Corning plate was coated with anti-CuMVTT antibody overnight at 4 °C. Then plate was blocked with PBS-0.15% Casein for 2 h at room temperature, after which CuMVTT-DF (CuMVTT as a control) vaccine was added to plate and serially diluted. After incubation at room temperature for an hour, human anti-RBD antibody (Sanyou biopharmaceuticals, catalog AHA004, Shanghai, China) was added and incubated for one hour. (https://doi.org/10.3390/vaccines9111287) |

# Animals and other organisms

Policy information about studies involving animals; ARRIVE guidelines recommended for reporting animal research

| | |
|---|---|
| Laboratory animals | female Drosophila melanogaster |
| Wild animals | the study did not involve wild anymals |
| Field-collected samples | the study did not involve samples collected from the field |
| Ethics oversight | no ethical approval or guidance was required since there are no ethical issues assumed for their use in research |

Note that full information on the approval of the study protocol must also be provided in the manuscript.
